# Mössbauer and Nuclear Resonance Vibrational Spectroscopy Studies of Iron Species Involved in N–N Bond Cleavage

**DOI:** 10.1021/acs.inorgchem.3c02594

**Published:** 2023-10-30

**Authors:** Aleksandra Wandzilak, Katarzyna Grubel, Kazimer L. Skubi, Sean F. McWilliams, Dimitrios Bessas, Atanu Rana, Stefan Hugenbruch, Abhishek Dey, Patrick L. Holland, Serena DeBeer

**Affiliations:** † Max Planck Institute for Chemical Energy Conversion, Mülheim an der Ruhr 45470, Germany; ‡ Faculty of Physics and Applied Computer Science, AGH University of Science and Technology, Krakow 30-059, Poland; § Department of Chemistry, Yale University, New Haven, Connecticut 06520, United States; ∥ Department of Chemistry, Carleton College, Northfield, Minnesota 55057, United States; ⊥ European Synchrotron Radiation Facility, Grenoble F-38043, France; # School of Chemical Science, Indian Association for the Cultivation of Science, Kolkata 700032, India

## Abstract

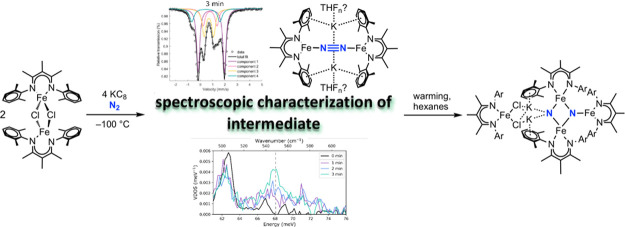

Diketiminate-supported iron complexes are capable of cleaving the strong triple bond of N_2_ to give a tetra-iron complex with two nitrides (Rodriguez et al.*, Science*, **2011**, 334, 780–783). The mechanism of this reaction has been difficult to determine, but a transient green species was observed during the reaction that corresponds to a potential intermediate. Here, we describe studies aiming to identify the characteristics of this intermediate, using a range of spectroscopic techniques, including Mössbauer spectroscopy, electronic absorption spectroscopy, Raman spectroscopy, nuclear magnetic resonance (NMR) spectroscopy, and nuclear resonance vibrational spectroscopy (NRVS) complemented by density functional theory (DFT) calculations. We successfully elucidated the nature of the starting iron(II) species and the bis(nitride) species in THF solution, and in each case, THF breaks up the multiiron species. Various observations on the green intermediate species indicate that it has one N_2_ per two Fe atoms, has THF associated with it, and has NRVS features indicative of bridging N_2_. Computational models with a formally diiron(0)–N_2_ core are most consistent with the accumulated data, and on this basis, a mechanism for N_2_ splitting is suggested. This work shows the power of combining NRVS, Mössbauer, NMR, and vibrational spectroscopies with computations for revealing the nature of transient iron species during N_2_ cleavage.

## Abstract 2

Mössbauer and NRVS spectroscopies, combined with density functional theory (DFT) calculations, are utilized to identify the key spectroscopic signature for iron−N_2_ complexes with weakened and with cleaved N−N bonds. Having established the spectroscopic signatures for well-characterized solid-state samples, this combined approach is then utilized to assess solution-state structures and to narrow down possible structures for an intermediate species formed during a N−N bond-cleaving reaction.

## Introduction

Nitrogen fixation (the conversion of inert N_2_ to more useful compounds) is one of the fundamental chemical reactions that supports biological systems.^1^ The vast majority of the nitrogen atoms in the environment are in the form of gaseous N_2_, which must be transformed into more accessible forms for incorporation into amino acids, nucleic acids, and other biomolecules.^2,3^ Chemists have shown that iron species are advantageous for both synthetic N_2_ fixation (through the Haber–Bosch process) and biological N_2_ fixation (through nitrogenase enzymes) to form ammonia.^4^ These catalysts use different mechanisms and different multiiron structures, but in both cases, the mysteries surrounding the atomic-scale mechanisms motivate chemists to better understand the interactions of iron with N_2_.

**Chart 1:**
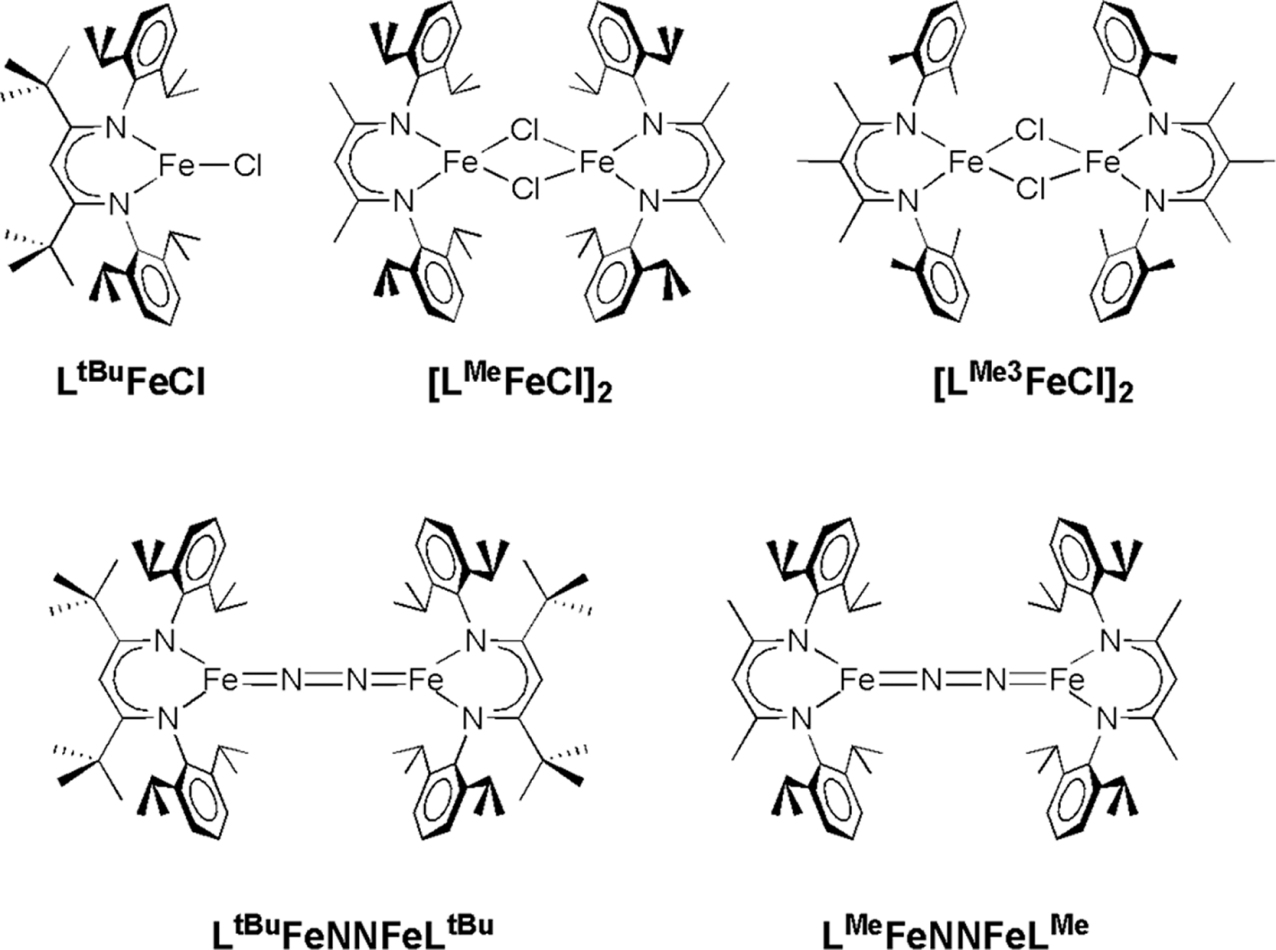
Definitions of Compounds and Ligands Used in this Study

One way to gain the necessary understanding is by studying synthetic iron–N_2_ coordination complexes, which demonstrate the capabilities of iron in different environments.^5−12^ In this context, complexes supported by the bidentate β-diketiminate ligands (Chart 1) have played an important role in assessing the impact of coordination number and multimetallic cooperation on the interactions of N_2_ with iron centers.^13,14^ Our studies in this area start with relatively simple iron(II) chloride species [L^R^FeCl]_*n*_ (L^R^ = β-diketiminates shown in Chart 1; *n* = 1, 2), which react with alkali metal reductants (e.g., potassium graphite, KC_8_) to give reduction of the iron site along with halide abstraction.^13,15−17^ Using L^*t*Bu^, KC_8_ reduction of the iron(II) chloride complex under N_2_ gives a diiron complex L^*t*Bu^FeNNFeL^*t*Bu^, in which N_2_ is symmetrically bridged with end-on interactions to each iron. In this binding mode, the N–N bond is weakened, as judged by the substantial lengthening of the bond (from 1.11 to 1.18 Å). The same behavior is observed for the smaller diketiminate ligand L^Me^, which also has *ortho*-isopropyl groups on the aryl rings, but the backbone has methyl groups rather than *tert*-butyl groups.

**Scheme 1:**
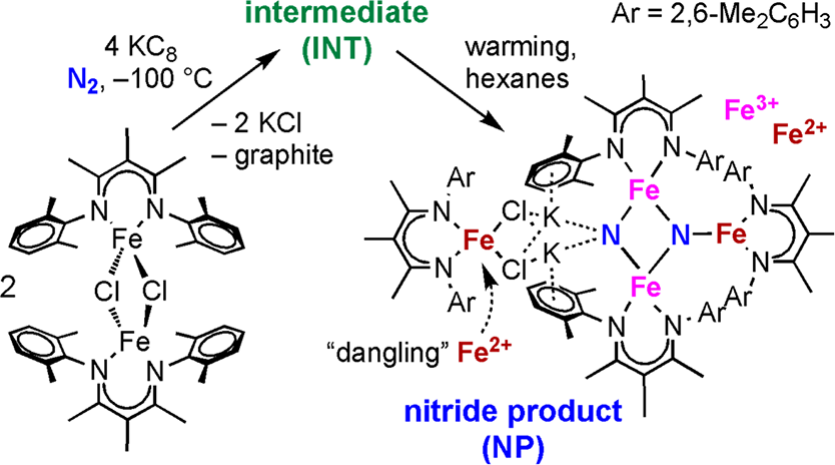
Cleavage of N–N Bonds by an Iron System Supported by L^Me3^

In this series of molecular iron complexes, we have tested the influences on N–N bond weakening by modulating the oxidation state, alkali metal, and bulk on the supporting ligand.^18^ One observation is that the reduction of the diiron core of isolable L^*t*Bu^FeNNFeL^*t*Bu^ and L^Me^FeNNFeL^Me^ with KC_8_ gives complexes that have formal oxidation states of iron(0). This reduction of the core weakens the N–N bond because of increased back-bonding from higher-energy d orbitals on the iron site. The core of the molecule incorporates the alkali metal cations, which heighten the back-bonding because the positive charge withdraws charge into the N_2_ unit, and the amount of additional weakening is similar for Na^+^, K^+^, Rb^+^, and Cs^+^.^16,17^ Most dramatically, decreasing the size of the supporting ligand to L^Me3^ and performing the same reduction with KC_8_ under N_2_ gives an iron/alkali metal cluster with two N_2_-derived nitrides in the core (nitride product or “NP,” Scheme 1).^18,19^ This complex transformation involves not only the cleavage of the N–N triple bond but also numerous redox steps with the cleavage of Fe–Cl bonds. Mössbauer spectroscopy of NP shows localized valences, with two identical iron(III) sites (shown in pink) and two iron(II) sites (shown in red-brown). One of the iron(II) sites (in the left of the diagram) is connected only by potassium ions (“dangling”) and can be removed using crown ethers or acids.^18,20−22^ The other iron(II) site (on the right of the diagram) is three-coordinate.

This series of steps is especially interesting because of the potential for insights into the mechanism of N–N bond cleavage in multiiron centers,^13,14^ which has particular relevance to the surface of the heterogeneous iron catalyst for the Haber–Bosch process. Namely, extensive studies on both model iron(0) surfaces and the “technical” catalyst indicate that the active catalyst has iron at an oxidation level between 0 and 1 and is the most active at surface features that are atomically rough.^23^ Though detailed kinetic models are available for N_2_ hydrogenation at these surfaces, details on the atomic-level structures of intermediates and transition states have been elusive.^24^ Computational studies have suggested possible structures for surface N_2_,^25^ but synthetic complexes, which are amenable to solution studies, are a promising route to characterize feasible pathways for N_2_ cleavage at multiiron sites.^10^

One interesting aspect of the reaction pathway in Scheme 1 is that the initial addition of potassium graphite (KC_8_) to a THF solution of [L^Me3^FeCl]_2_ at a low temperature generates a green mixture that is visually different from red NP.^18,19^ During the synthesis of NP, the THF mixture is dried under vacuum and redissolved in hexanes to give isolable NP. The nature of INT has remained unknown, despite the potential that its identification holds for insights into the mechanism of N_2_ cleavage.

In cases like this, when crystallography is not possible for transient intermediates, spectroscopic tools are key for identifying them, and vibrational spectroscopy is commonly used. However, traditional methods like infrared (IR) and Raman spectroscopy have limitations: both are limited by their respective selection rules, which render many normal modes invisible in the spectra, and resonance Raman spectroscopy also requires the presence of a suitably strong chromophore that is coupled to the relevant vibration. For this reason, we have sought additional tools to diversify and enrich the information on iron–N_2_ intermediates. One of these tools is ^57^Fe Mössbauer spectroscopy, which probes the electron density and electric field gradient at the iron nuclei in the compound of interest.^26^ Another is X-ray emission spectroscopy (XES),^27−29^ which queries the energies of valence-shell electrons as they relax to core–shell orbitals. Notably, we were able to identify a band in the valence-to-core (VtC) XES spectra of Fe–N_2_ species that correlates with the extent of N–N weakening and cleavage.^30^ However, overlap with other ligand contributions and relatively low experimental resolution (due to the short 1s core hole lifetime) can limit the broad applicability of this method.

In this paper, we complement these techniques with nuclear resonance vibrational spectroscopy (NRVS),^31^ which is specific for Mössbauer active nuclei like ^57^Fe. A major advantage over other vibrational spectra (IR absorption, Raman) is that NRVS has no symmetry-based selection rules that prohibit the observation of certain bands, and it shows all bands that involve the motion of the iron nuclei, with an intensity that corresponds to the Fe displacement in the vibration.^32−36^ Since NRVS shows only vibrations involving Fe motion, ligand-based normal modes are minimized, and the use of labeled ^15^N_2_ in the synthesis allows us to identify N-sensitive modes that involve N_2_. The ability to calculate NRVS and parallel Mössbauer spectra with DFT enables computational models to be spectroscopically validated. Here, we complement NRVS in a limited set of complexes with resonance Raman data for a more rigorous evaluation of the observed vibrational modes.

Overall, this article utilizes a combined spectroscopic and computational approach. First, we study well-characterized molecular iron–N_2_ complexes in the solid state in order to establish the validity of the method and provide spectroscopic fingerprints for iron–N_2_ complexes with weakened and cleaved N–N bonds. Having established these fingerprints for solid-state samples, we then extend our approach to query the changes in the structures of these compounds in THF solution. The combined spectroscopic and computational approach is finally used in an attempt to narrow the possible structures for the green intermediate INT, formed during the N–N bond-cleaving reaction. In this way, we highlight the benefits and limitations of the approach while providing insights into the complex solution reactivity of iron β-diketiminate complexes.

## Experimental Section

### Synthesis

L^*t*Bu^FeCl,^37^ L^*t*Bu^FeNNFeL^*t*Bu^,^15^ K_2_L^*t*Bu^FeNNFeL^*t*Bu^,^15^ L^Me^FeNNFeL^Me^,^16^ [LFeCl]_2_,^19^ and NP^19^ were prepared using published methods. The purity of the compounds was verified by ^1^H NMR spectroscopy and Mössbauer spectroscopy at Yale prior to shipping to the synchrotron beamlines for analysis. All syntheses were performed in an MBraun glovebox under a N_2_ atmosphere maintained at or below 1 ppm of O_2_. All glassware were oven-dried at 150 °C for at least 12 h before use. Hexanes, diethyl ether, and benzene were purified by passage through activated alumina and Q5 columns. Tetrahydrofuran (THF) was dried by distilling from Na/benzophenone. All solvents were stored over activated 3 Å molecular sieves and passed through a plug of activated alumina before use. Deuterated benzene was dried over activated alumina and then filtered before use. THF-*d*_8_ was dried over CaH_2_ and then over Na/benzophenone, and it was vacuum-transferred to a storage container before use. Graphite, Celite, and 3 Å molecular sieves were dried at 300 °C under vacuum for >12 h. Potassium graphite (KC_8_) was prepared by heating stoichiometric amounts of potassium and graphite to 145 °C under an argon atmosphere. KC_8_ ignites on contact with air and moisture. Therefore, extreme care must be taken when synthesizing and handling it. ^1^H NMR spectra were recorded on either an Avance 400, Avance 500, or Agilent 500 spectrometer and are referenced to residual C_6_D_5_H at δ 7.16 ppm. UV–vis spectra were recorded on a Cary 50 spectrometer using Schlenk-adapted quartz cuvettes with a path length of 1 mm.

^15^N-labeled samples were prepared by adding KC_8_ to the appropriate iron precursor under an atmosphere of ^15^N_2_ (Cambridge Isotope Laboratories). This was accomplished by filling a bulb (20–60 mL volume) with ^15^N_2_ and bringing it into an Ar-filled glovebox and attaching it to a three-necked flask with the iron precursor in an appropriate solvent and the other necks having a vacuum adapter and a solid addition bulb containing KC_8_. The headspace was evacuated and refilled with ^15^N_2_, and the sample was stirred for several minutes at room temperature to dissolve ^15^N_2_. Then, the mixture was frozen in a cold well (77 K). KC_8_ was added to the frozen solution, which was then slowly thawed with stirring to give the intermediate (INT). For UV–vis and NMR experiments, INT samples were kept below −50 °C, and aliquots were filtered through a pipette filter precooled in the cold well.

### Nuclear Resonance Vibrational Spectroscopy

Solid samples of L^*t*Bu^FeNNFeL^*t*Bu^, K_2_L^*t*Bu^FeNNFeL^*t*Bu^, NP, and [L^Me3^FeCl]_2_ were ground powders utilized without any dilution. Solution samples of NP and INT were measured on samples with uniform ^57^Fe labeling. For the solution samples in THF, the concentrations were as follows: ^14^N LFeNNFeL, 45 mM in ^57^Fe; ^15^N LFeNNFeL, 56 mM in ^57^Fe; NP, both ^14^N and ^15^N, 50 mM in ^57^Fe; [L^Me3^FeCl]_2_ solution, 116 mM in ^57^Fe; INT: ^14^N time points of the reaction, 48 mM in ^57^Fe; and ^15^N time points of the reaction, 49 mM in ^57^Fe. For NRVS measurements, the samples were transferred into Al holders with a sample compartment of 2 mm × 3 mm × 10 mm. Sample holders were sealed with a Kapton tape, and the samples were stored in liquid N_2_ upon preparation. All NRVS spectra except for solid K_2_L^*t*Bu^FeNNFeL^*t*Bu^ and solid NP were recorded at the ESRF. Solid NP and K_2_L^*t*Bu^FeNNFeL^*t*Bu^ were measured at PETRA III. At ESRF ID18 (ring operating in 16 bunch mode with 90 mA current), the radiation was monochromatized with a Si(111) double-crystal high heat load monochromator (HHLM), followed by a high-resolution monochromator (HRM) providing 14.412 keV photons with 0.6 meV resolution. The beam impinging on the sample was 1.5 mm (h) × 0.5 mm (v), and the delayed radiation was recorded with an avalanche photodiode. Simultaneously, the nuclear forward-scattering signal was measured to provide an instrumental function. The samples were cooled with a liquid He flow cryostat, with the typical temperature on the sample in the range of 20–30 K. Partial vibrational density of states (VDOS) were extracted from NRVS data with the use of a graphical user interface based on the DOS software.^38^

At PETRA III P01 (ring operating in 40 bunch mode with 95 mA current), the radiation was monochromatized with a Si(111) double-crystal high heat load monochromator (HHLM), followed by Si(10 6 4) and Si(4 0 0) high-energy resolution monochromator (HRM) providing 14.412 keV photons with 1.0 meV resolution. The beam impinging on the sample was 2.5 mm (h) × 0.3 mm (v), and the delayed radiation was recorded with an avalanche photodiode. The samples were cooled down with the use of a liquid He flow cryostat, with the typical temperature on the sample around 8 K. Partial vibrational density of states (VDOS) were extracted from NRVS data with the use of a modified version of the DOS software.^38^ The area of extracted VDOS was normalized to unity. For each energy point in the VDOS, the relative error bar is equal to the relative error bar of the same point in the measured spectrum. The error bars in the measured spectra are given as the square root of the total counts measured in each channel.

### Mössbauer Spectroscopy

All ^57^Fe Mössbauer spectra were recorded on samples cooled to 80 K in conventional spectrometers with alternating constant acceleration, with a 0.07 T applied magnetic field. Isomer shifts were referenced to α-^57^Fe foil at 298–300 K. Mössbauer spectra were fitted using the programs MF (written by Eckhard Bill) or WMoss (SEECo) using Lorentzian line shapes, constrained to have the same width for both sides (Γ_L_ = Γ_R_).

### Resonance Raman spectroscopy

Resonance Raman samples of L^Me^FeNNFeL^Me^, L^Me^Fe^15^N^15^NFeL^Me^, K_2_L^Me^FeNNFeL^Me^, and K_2_ L^Me^Fe^15^N^15^NFeL^Me^ were 10 mM in pentane solution and were sealed in 5 mm OD NMR tubes. The resonance Raman (rR) experiment was performed by using a Coherent Sabre Kr ion laser and a Princeton Instruments' Trivista 555 triple monochromator spectrograph fitted with a Pixies Excelion CCD camera for all complexes. The sample was placed in standard 5 mm NMR borosilicate glass tubes, which were flame-sealed. Before each measurement, the spectrometer was calibrated with a naphthalene standard. All rR spectra were collected at 77 K (liquid N_2_), and the power was less than 50 mW to minimize photodamage. Furthermore, rR spectra on several random spots were recorded in order to check for the consistency of the spectrum.

### X-ray Spectroscopy (Fe K-Edge XAS and Fe VtC XES)

XAS and XES spectra were obtained for [L^Me3^FeCl]_2_ as a solid and in THF. For XAS measurements, the solid sample of [L^Me3^FeCl]_2_ was ground, diluted with solid boron nitride, transferred to 1 mm Al spacer, sealed with 38 μm Kapton tape, and immediately frozen in liquid N_2_. For XES measurements, the solid sample of [L^Me3^FeCl]_2_ was ground and transferred to 1 mm Al spacer, sealed with 38 μm Kapton tape, and immediately frozen in liquid N_2_. The solution sample of [L^Me3^FeCl]_2_ (37 mM in THF) was transferred into a 80 μL Delrin cup and sealed with 38 μm Kapton tape inside a glovebox and immediately frozen in liquid N_2_ immediately outside the glovebox. Samples were transported to the synchrotron in LN_2_ dry shipping dewars, loaded under LN_2_, and transferred directly to the beamline cryostats, where the samples were measured at temperatures of ∼10–20 K.

EXAFS data were collected at the ESRF beamline BM-23 (6 GeV, 200 mA). A Si(111) monochromator was used for energy selection. The incident energy was calibrated by setting the first inflection point of Fe at 7111.2 eV. Focusing mirrors were used to achieve a 0.5 mm (v) × 10 mm (h) beam spot at the sample. When necessary, to minimize the beam damage, Al filters were inserted before the sample to attenuate the incident beam. XAS scans were taken from 7056 to 7970 eV with a 1.4 eV step size and a 3 s exposition time per point. Data were collected in transmission mode (for the solid sample) and fluorescence mode (for the solution sample). The Demeter package was used for background subtraction and for the analysis of the EXAFS region.^39^

Fe Kα-detected XAS and Kβ XES data were collected at the ESRF beamline ID-26 (6 GeV, 200 mA). A Si(311) or Si(111) monochromator was used for energy selection of the incident beam for Kα and Kβ measurements, respectively. X-ray emission was detected by using a crystal spectrometer with five spherically bent crystal analyzers in combination with a silicon drift detector. Ge(220) and Ge(620) reflections were utilized for Kα and Kβ detection, respectively. The incident energy was calibrated by setting the first inflection point of an iron foil to 7112.2 eV. Focusing mirrors were used to achieve 0.3 (v) × 1 (h) mm^2^ beam spot at the sample. Aluminum filters were inserted before the sample to attenuate the incident beam. Fe Kα-detected XAS and Kβ XES spectra were measured utilizing previously described data collection and processing protocols.^40,41^

### DFT Calculations

All calculations were performed with the ORCA program package (version 4.2.1).^42,43^ Geometry optimization and numerical frequency calculations were performed using the BP86 functional^44,45^ with the relativistically recontracted ZORA-def2-TZVP basis set on Fe and N atoms and ZORA-def2-SVP basis set on the remaining atoms along with the SARC/J auxiliary basis set. Default geometry and SCF convergence settings were used for all of the geometry optimizations. Dispersion effects were accounted for using D3BJ.^46,47^ Calculations were performed with fine integration grids (grid 4). Calculations of Mössbauer parameters^48^ were perfomed using the B3LYP functional^49,50^ with the ZORA-def2-TZVP basis set and D3BJ dispersion corrections. Calculations were performed with fine integration grids (grid 4), but for Fe atoms, tighter grids and accuracy were used (SpecialGridIntAcc 7). Tight SCF settings were used for all single-point calculations. For the prediction of isomer shifts, a calibration based on β-diketiminate complexes was utilized.^51^ For solvation effects, the conductor-like polarizable continuum model (CPCM)^52^ with default settings for THF was used. NRVS spectra were simulated according to a procedure described in detail elsewhere.^53^ All calculated NRVS spectra were area-normalized.

## Results

### NRVS of Solid Reference Samples

**Figure 1:**
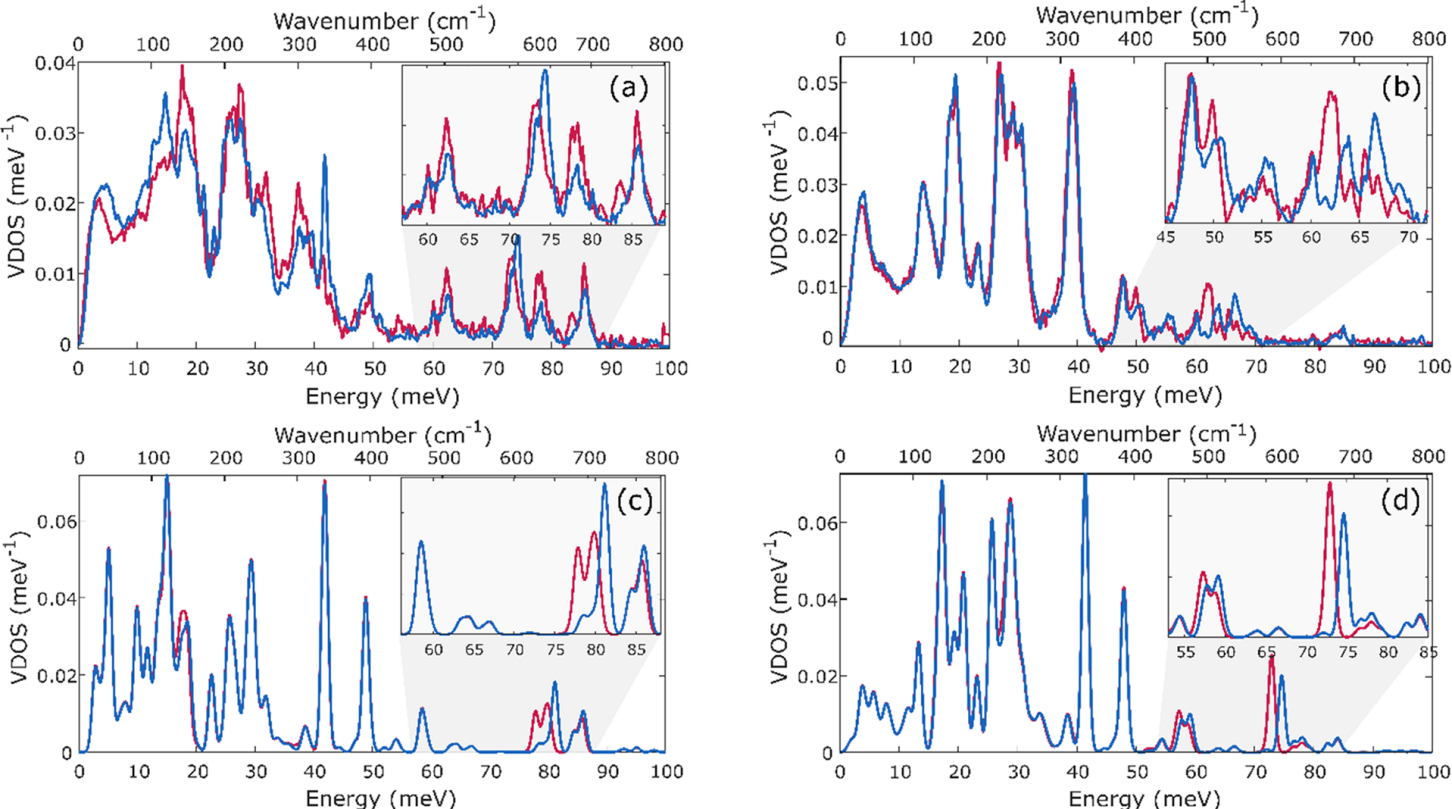
Experimental NRVS spectra of solid L^*t*Bu^FeNNFeL^*t*Bu^ (a) and K_2_L^*t*Bu^FeNNFeL^*t*Bu^ (b) along with the calculated spectra of L^*t*Bu^FeNNFeL^*t*Bu^ (c) and K_2_L^*t*Bu^FeNNFeL^*t*Bu^ (d). Spectra of compounds with bridging ^14^N_2_ are shown in blue and those with bridging ^15^N_2_ are shown in red. Versions of the experimental NRVS spectra (a, b) showing error bars are provided in Figure S1.

Figures 1a,b shows the experimental NRVS spectra of the formally diiron(I) complex L^*t*Bu^FeNNFeL^*t*Bu^ and the formally diiron(0) K_2_L^*t*Bu^FeNNFeL^*t*Bu^, respectively. Comparison of the NRVS spectra of L^*t*Bu^FeNNFeL^*t*Bu^ and K_2_L^*t*Bu^FeNNFeL^*t*Bu^ shows three important observations. First, in the low-energy region (below 50 meV/403 cm^–1^), the NRVS features are sharper in K_2_L^*t*Bu^FeNNFeL^*t*Bu^ than in L^*t*Bu^FeNNFeL^*t*Bu^. Second, the energies of high-energy bands change. Specifically, a group of peaks visible in the energy range from 60 to 85 meV (484–686 cm^–1^) in the spectrum of L^*t*Bu^FeNNFeL^*t*Bu^ (inset, Figure 1a) shifts to energies less than 70 meV (565 cm^–1^) in K_2_L^*t*Bu^FeNNFeL^*t*Bu^ (inset, Figure 1b) in which the core is reduced and bound to two potassium ions. Third, the substitution of ^14^N_2_ (blue spectra) for ^15^N_2_ (red spectra) results in observable differences in the NRVS spectra of both complexes, particularly in the high-energy region (>50 meV/403 cm^–1^). In L^*t*Bu^FeNNFeL^*t*Bu^, ^15^N labeling alters both the energies and intensities of the features in the 60–85 meV (484–686 cm^–1^) region, with the average energy of the bands shifting to lower energy by 1–2 meV (8–16 cm^–1^). Similarly, the peaks observed at 63.5 meV (512 cm^–1^) and 66.5 meV (536 cm^–1^) in K_2_L^*t*Bu^FeNNFeL^*t*Bu^ shift to 62.1 meV (501 cm^–1^) and 65.5 meV (528 cm^–1^) with ^15^N substitution.

In order to interpret the NRVS spectra and assign the observed features, we used density functional theory (DFT) calculations of L^*t*Bu^FeNNFeL^*t*Bu^ and K_2_L^*t*Bu^FeNNFeL^*t*Bu^ by performing geometry optimizations on the known crystal structures. As previously reported, the electronic structure of L^*t*Bu^FeNNFeL^*t*Bu^ is best described as a septet ground state with two high-spin Fe^II^ (S_Fe1_ = S_Fe2_ = 2) that are each strongly antiferromagnetically coupled to a triplet N_2_^2–^ (S_N_2__ = 1) (calculations used charge = 0, multiplicity = 7), while the electronic structure of K_2_L^*t*Bu^FeNNFeL^*t*Bu^ is best described as a quintet ground state in which the core is two electrons further reduced (charge = 0, multiplicity = 5).^16,54^

In order to assess the appropriateness of the calculated electronic structures for analyzing the experimental NRVS spectra, we first used the optimized structures to calculate Mössbauer parameters. The experimental Mössbauer spectra and their fits are provided in the Supporting Information (Figures S2 and S3). For L^*t*Bu^FeNNFeL^*t*Bu^, the calculated values were δ = 0.67 and Δ*E*_Q_ = 1.55 mm/s. These compare favorably with the experimental values of δ = 0.61, Δ*E*_Q_ = 1.63, and δ=0.73 mm/s, Δ*E*_Q_ = 1.61 mm/s, within the expected accuracy of ±0.1 mm/s for δ and ±0.4 mm/s for Δ*E*_Q_.^51,55,56^ For K_2_L^*t*Bu^FeNNFeL^*t*Bu^, a similarly strong correspondence between theory and experiment was obtained with the calculated values of δ = 0.50 and Δ*E*_Q_ = 2.19 mm/s and experimental values of δ=0.50 and Δ*E*_Q_ = 2.27 mm/s. This agreement indicates that the DFT modeling of L^*t*Bu^FeNNFeL^*t*Bu^ and K_2_L^*t*Bu^FeNNFeL^*t*Bu^ reflects the electronic structure accurately enough to be a good basis for the calculation of the NRVS spectra and is also consistent with previous computational studies on these complexes.^17^

**Figure 2:**
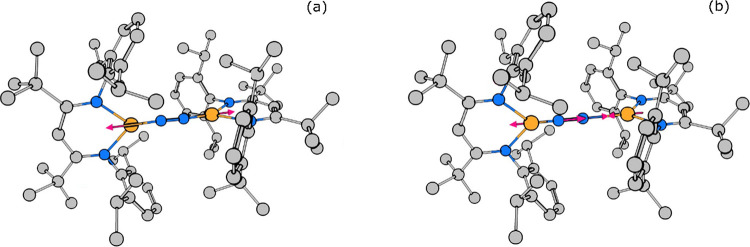
Arrow-style representation of the key calculated core vibrational modes of L^*t*Bu^FeNNFeL^*t*Bu^. (a) Fe–N=N–Fe symmetric stretching mode at 41.9 meV (338 cm^–1^). (b) Fe–N=N–Fe asymmetric stretching mode at 81.2 meV (655 cm^–1^). Animations of normal modes are available in the Supporting Information.

Utilizing the same optimized structures that were employed for the Mössbauer calculations, NRVS spectra were then calculated for L^*t*Bu^FeNNFeL^*t*Bu^ and K_2_L^*t*Bu^FeNNFeL^*t*Bu^, with the incorporation of both ^14^N_2_ and ^15^N_2_ in the bridge, as shown in Figure 1c,d. Overall, the calculated spectra agree reasonably well with the experimental trends. We note that the calculated vibrational frequencies are somewhat overestimated, as may be expected,^57^ but we have refrained from using a scaling factor. L^*t*Bu^FeNNFeL^*t*Bu^ has somewhat lower intensity and is broader than K_2_L^*t*Bu^FeNNFeL^*t*Bu^ in both the experimental and calculated NRVS spectra. As the NRVS intensity is proportional to the iron displacement in a given normal mode, this observation suggests that in the case of L^*t*Bu^FeNNFeL^*t*Bu^, the decreased intensity may result from a less rigid structure, which allows for Fe contributions to mix with a larger range of normal modes, hence resulting in a broader and less intense NRVS spectrum. There is greater structural rigidity upon the incorporation of potassium, since the potassium ions are bound tightly to the aryl groups and to the bridging N_2_.^15^ In addition, the highest energy features of L^*t*Bu^FeNNFeL^*t*Bu^ (at ∼75–85 meV/∼605–686 cm^–1^) appear at a higher absolute energy than those observed in K_2_L^*t*Bu^FeNNFeL^*t*Bu^ (at ∼70–80 meV/∼565–645 cm^–1^). These high-energy bands are also the most sensitive to ^15^N substitution, and we assign them to the modes dominated by Fe–N stretching. Based on computations, the largest spectral differences are assigned to the asymmetric stretching modes (Figure 2b), which appear at the highest energy in both complexes and shift downward by ∼2 meV (16 cm^–1^) upon ^15^N substitution. The corresponding symmetric modes (Figure 2a) appear at a lower energy (at ∼40–50 meV/323–403 cm^–1^ in both complexes) and show only modest shifts (∼0.5 meV/4 cm^–1^) relative to the asymmetric mode. This is also consistent with the experiment where the clearest spectral differences are seen at the highest energies.

**Figure 3:**
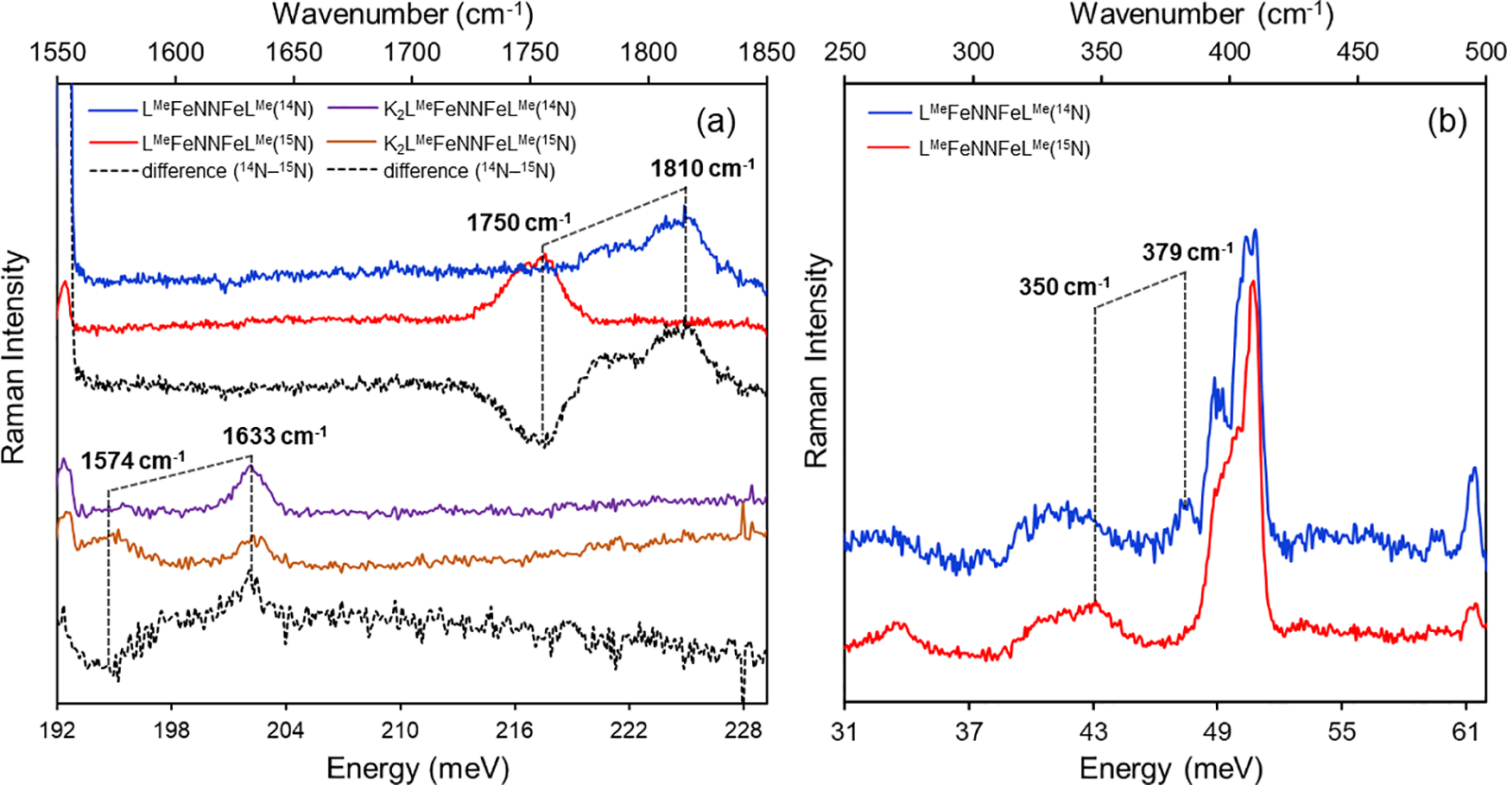
Resonance Raman spectra of samples in frozen pentane (77 K): (a) stacked spectra (520 nm, 30 mW) of L^Me^FeNNFeL^Me^ (dark blue), ^15^N-substituted L^Me^FeNNFeL^Me^ (red), and their difference (dashed black); stacked spectra (413.1 nm, 10 mW) of K_2_L^Me^FeNNFeL^Me^ (purple), ^15^N-substituted K_2_L^Me^FeNNFeL^Me^ (brown), and their difference (dashed black). (b) Stacked spectra of L^Me^FeNNFeL^Me^ (647 nm, 50 mW) (blue) and its ^15^N-substituted analogue (red). The dotted black line highlights the changes upon ^15^N substitution.

Here, it is useful to contrast the NRVS data to the corresponding resonance Raman data in an effort to provide assignments for the bands. These have been reported previously for L^Me^FeNNFeL^Me^ and L^*t*Bu^FeNNFeL^*t*Bu^ using an excitation wavelength (λ_ex_) of 406.7 nm; there was a band assigned to the N–N stretching vibration at 1810 cm^–1^ (L^Me^) or 1778 cm^–1^ (L^*t*Bu^) that shifted to 1745 cm^–1^ (L^Me^) or 1718 (L^*t*Bu^) cm^–1^ with ^15^N isotope substitution.^15^ Here, we remeasured the resonance Raman data of pentane solutions of the ^14^N- and ^15^N-labeled L^Me^FeNNFeL^Me^ complexes at λ_ex_ = 520 nm, which are in resonance with the broad CT band (440–600 nm) observed in solution. There is a feature at 1810 cm^–1^ (225 meV) that shifts to 1750 cm^–1^ (217 meV) upon ^15^N labeling (Figure 3a, dark blue to red lines; difference spectra in dashed black). Another set of vibrations is observed around 340 and 397 cm^–1^, but none of these vibrations are sensitive to ^15^N substitution (Figure S4a,b), indicating that these are likely to be associated with the β-diketiminate ligand. Supporting this idea, the DFT calculations predict numerous normal modes in this region that are localized in the supporting ligand, and these are also observed in the NRVS spectra as noted above. The asymmetric modes at 70–80 meV (565–645 cm^–1^) observed in the NRVS spectra are not seen in the Raman spectra, indicating that these are forbidden by Raman selection rules and supporting the assignment as the asymmetric combination of Fe–N(N_2_) stretching motions shown in Figure 2b.

We also measured an excitation profile for L^Me^FeNNFeL^Me^ using a set of excitation wavelengths across the broad absorption band around 500 nm (Figure S4c). The enhancement of both the ^15^N-sensitive N–N and ^15^N-insensitive β-diketiminate ligand vibrations between 480 and 550 nm suggests that this absorption feature represents a charge transfer that involves the β-diketiminate ligand and the FeNNFe bridge. A charge transfer from the occupied β-diketiminate ligand orbital(s) to the unoccupied orbital(s) of the Fe–NN–Fe unit is thus the most likely origin of this absorption feature. The Raman spectra of L^Me^FeNNFeL^Me^ collected by excitation into the lower energy end of the absorption spectra (λ_ex_ > 520 nm) show an additional band at 379 cm^–1^ that shifts to 350 cm^–1^ with ^15^N substitution (Figure 3b). The intensity of the 379 cm^–1^ band maximizes with longer-wavelength excitation (Figure S4c), indicating that there is another charge transfer state at lower energy with different excited state distortions involving the Fe–N bond. Overall, the resonance Raman data complement the vibrational data obtained from NRVS and additionally provide insights into the origin of CT transitions in this family of complexes.

Excitation into the prominent absorption band of the K_2_L^Me^FeNNFeL^Me^ complex at 700 nm (Figure S5) did not reveal any N-isotope-sensitive vibration, suggesting that this transition does not include the Fe–NN–Fe unit. When excited into the higher energy transition in the near-UV region, the resonance Raman spectra of K_2_L^Me^FeNNFeL^Me^ (λ_ex_ = 413.1 nm) show a band assigned to the N–N stretching vibration at 1633 cm^–1^, which shifts to 1574 cm^–1^ on ^15^N substitution (Figure 3a; purple to brown, difference in dashed black).^16^ In addition, the brown spectrum contains a band at 1633 cm^–1^ along with one at 1574 cm^–1^, indicating residual ^14^N-labeled N_2_ in the sample. No other N-isotope-sensitive vibration could be detected. These provide clear signatures for N–N stretching but are too high in energy to have substantial NRVS intensity and also are likely to have little iron displacement. Thus, the resonance Raman and NRVS data are again complementary to each other.

**Figure 4:**
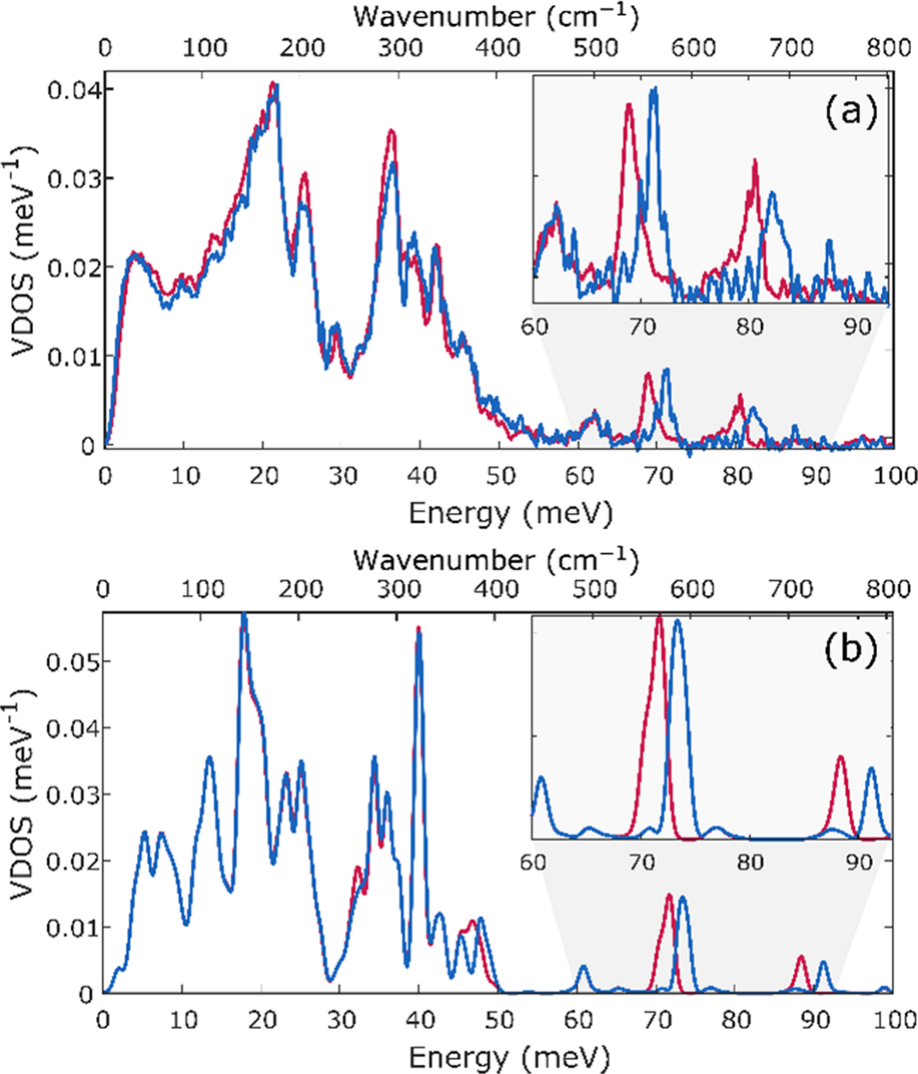
Experimental NRVS spectrum of solid NP (a) along with the calculated spectrum (b). Spectra of compounds with bridging ^14^N_2_ are shown in blue and those with bridging ^15^N_2_ are shown in red. Error bars for the experimental NRVS spectra (a) are provided in Figure S15a.

**Figure 5:**
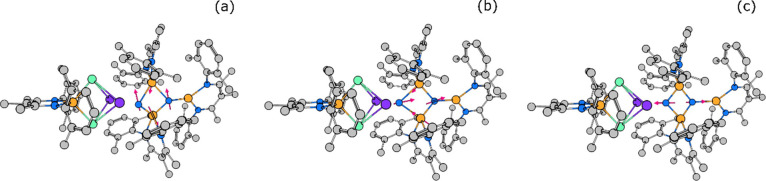
Arrow-style representation of key vibrational modes of NP. The three pictures show the wagging mode of the Fe_3_N_2_ unit at 73.0 meV (589 cm^–1^) (a) and 74.0 meV (597 cm^–1^) (b) and the Fe_3_N_2_ asymmetric stretch of the trimeric iron unit at 91.2 meV (736 cm^–1^) (c). Animations of the depicted normal modes are available as part of the Supporting Information.

Having established the ability of NRVS to identify the FeNNFe-specific vibrational modes, in the next step, we extended this approach to the tetra-iron complex NP, in which the N–N bond has been fully cleaved to form nitrides by virtue of the smaller β-diketiminate L^Me3^ (Scheme 1). DFT computations used a model of NP in which the two high-spin iron(II) centers and the two high-spin iron(III) centers are spin-aligned (multiplicity: 19); even though magnetic susceptibility studies^19^ show that there is antiferromagnetic exchange coupling within the core, these small energy differences (*J* = −288 and −98 cm^–1^, where *J* = −(*E*_HS_ – *E*_BS_)/(*S*_A_ + *S*_B_)^2^ and *E*_HS_ and *E*_BS_ are the energies of the high-spin and broken-symmetry solutions, respectively) are expected to influence neither Mössbauer nor vibrational spectra significantly. As shown in Figure S15 and Table S6, the calculated Mössbauer parameters showed a strong correlation with experiment, thus further validating the use of the calculated electronic structure for NRVS calculations. Figure 4a depicts the experimental NRVS for the samples generated from ^14^N_2_ and from ^15^N_2_. Note that ^15^N is incorporated only into the nitride bridges, since the other N atoms in the complex are not derived from ^15^N_2_. The spectra show clear changes between the isotopologues, with the peaks at 71.3 and 82.4 meV (575 and 665 cm^–1^) shifting to 68.9 and 80.6 meV (556 and 650 cm^–1^) with ^15^N labeling. Figure 4b shows the corresponding calculated spectra, which again show excellent agreement with experiment. While the calculated energies are somewhat overestimated, no scaling has been applied, and the general trends are clearly captured. Using the computations, we are able to assign the highest energy feature at ∼90 meV (726 cm^–1^) to the Fe_3_N_2_ asymmetric stretching mode of the trimeric iron unit (Figure 5). At a lower energy of ∼73 meV (589 cm^–1^), the calculations show a wagging mode of the Fe_3_N_2_ unit, which corresponds to the observed lower-energy NRVS band. There is also an isotope-insensitive band at ∼60 meV (484 cm^–1^), which is attributed to Fe–N(L^Me3^) stretching. These observations highlight the ability of ^15^N labeling to unambiguously identify the Fe–N(nitride) bands in the NRVS spectra.

In this section, we have established the ability of NRVS to identify Fe–N-related modes in crystallographically characterized iron complexes with N_2_-derived ligands. The NRVS spectra showed distinct changes as N_2_ became more activated. From L^*t*Bu^FeNNFeL^*t*Bu^ and K_2_L^*t*Bu^FeNNFeL^*t*Bu^, clear shifts in the asymmetric FeNNFe stretches to lower energy were observed. Further spectral changes were seen for NP, in which N_2_ is fully cleaved into a nitride complex. In this case, the asymmetric stretches of the Fe_3_N_2_ core show the greatest sensitivity of the frequency to ^15^N labeling. These observations highlight the ability of NRVS to serve as a probe of species that are formed during iron-mediated N–N bond cleavage reactions.

### Solution Structure of [L^Me3^FeCl]_2_

In the following sections, we evaluate the solution transformation of [L^Me3^FeCl]_2_ to a reduced species that binds and cleaves N_2_ to generate NP. The steps of this reaction and the conditions used will be described below. However, before evaluating the intermediates and the mechanism, it was necessary to evaluate the nature of the starting material and product in THF, the solvent used in the reaction that generates INT and NP. Because the THF adducts of each of these species have not been crystallographically characterized, the combined spectroscopic studies are valuable methods for elucidating the solution structures. In addition, this serves as a testing ground for the characterization of the more complicated intermediate(s) in the mechanism of N_2_ cleavage.

First, we evaluated the starting material. It is relevant that the ^1^H NMR spectrum of [L^Me3^FeCl]_2_ is different between the solutions in C_6_D_6_ (noncoordinating) and THF-*d*_8_ (coordinating) solvents (Figure S6), suggesting that the structure changes. Therefore, we first used Mössbauer spectroscopy in an effort to gain insight into the change. Figure S7 shows the Mössbauer spectra of [L^Me3^FeCl]_2_ as a solid and as a solution in THF. The spectra were very similar, with a slight decrease in the isomer shift (solid: δ = 0.93 mm/s; THF: δ = 0.90 mm/s) and no significant change in the quadrupole splitting (solid: Δ*E*_Q_ = 2.14 mm/s; THF: Δ*E*_Q_ = 2.13 mm/s). Therefore, Mössbauer is not effective for distinguishing whether the dimer is broken up in solution.

**Figure 6:**
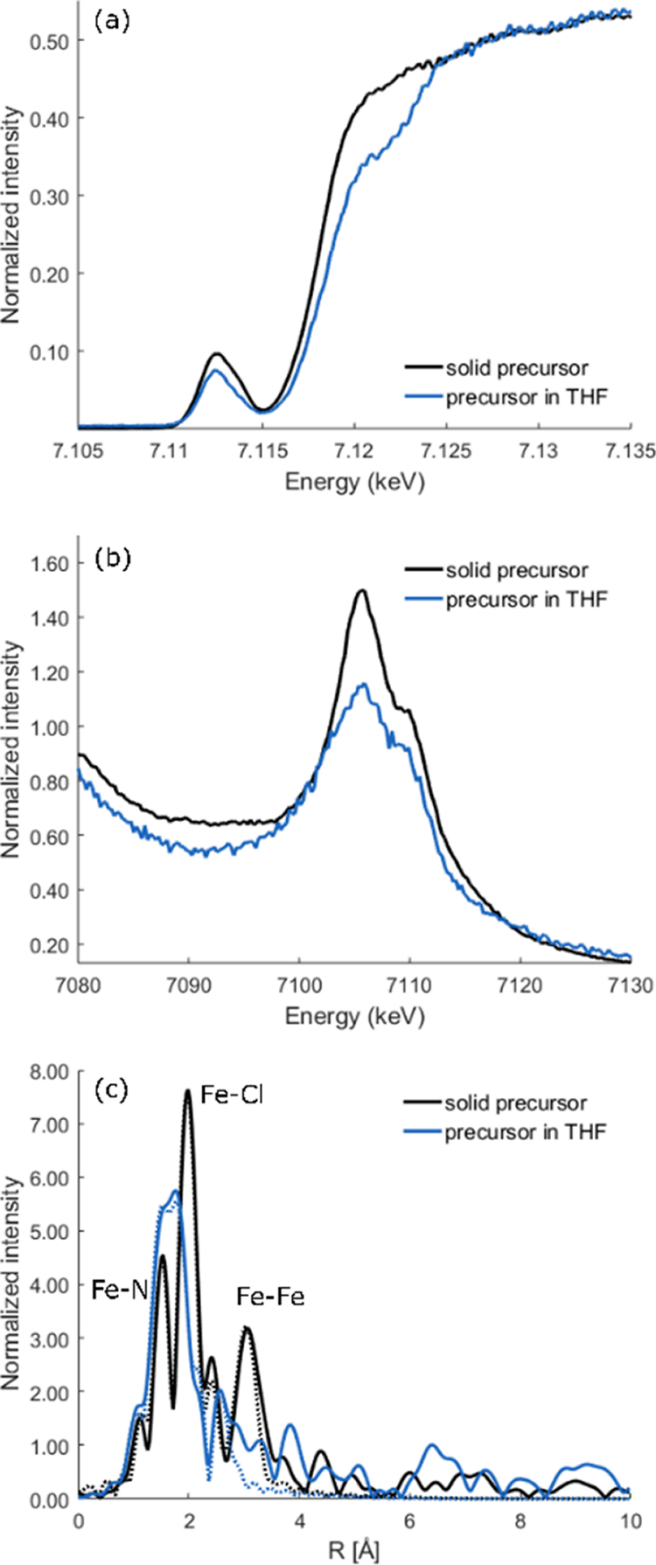
Fe Kα-detected XAS (a), VtC XES (b), and Fourier-transformed *k*^3^-EXAFS spectra (c) comparing the solid [L^Me3^FeCl]_2_ (black) to the solution in THF (blue). For the transformed spectra, experimental data are shown as solid lines and the corresponding fits as dotted lines.

On the other hand, Fe K-edge XAS showed more significant differences between the solid [L^Me3^FeCl]_2_ and its solution in THF. Figure 6a compares the normalized Fe Kα*-*detected XAS spectra. The solution sample in THF has a less intense pre-edge region, but the average pre-edge energy stays approximately the same as that of the sample in the solid state. This suggests that there are changes in the local coordination environment with no change in the overall oxidation state. This hypothesis is further supported by the changes in the rising edge region. While the white line features (at ∼7130 eV) are the same for the solid and solution samples, the rising edge has clearly shifted, with the solution sample showing a higher energy rising edge feature. Based on a comparison to the literature Fe K-edge data^58,59^ this is consistent with the replacement of chloride by a lighter scatterer, and we hypothesized that this is an oxygen atom from a coordinated THF. Coordination of THF in diketiminate-supported iron complexes has been observed.^60−65^

The proposed change in the first coordination sphere of [L^Me3^FeCl]_2_ upon dissolution of the sample in THF is further supported by the VtC XES and Fe K-edge EXAFS data. Figure 6b shows a comparison of the normalized VtC XES data of the solid and the solution samples. The loss of intensity at 7108 eV is consistent with the loss of Cl,^40,66^ while the presence of a shoulder at ∼7100 eV suggests coordination of a ligand with an ionization energy that is distinct from either L^Me3^ or Cl,^67^ presumably THF. This is further supported by the EXAFS data, the Fourier transforms of which are shown in Figure 6c. These data clearly show that an Fe–Cl vector is lost, and an additional light atom the scatterer is present. Further, EXAFS spectra show a loss of an ∼3.3 Å Fe–Fe vector, indicating that the [L^Me3^FeCl]_2_ dimer dissociates in solution. A summary of the EXAFS fits is provided in the Supporting Information (Figure S8 and Tables S4–S5). These data indicate that the precursor in THF is best described as a L^Me3^Fe(Cl)(THF) structure with three N/O light atom scatterers at ∼2.00 Å (two from L^Me3^ and one from THF) and one Cl at ∼2.25 Å.

**Figure 7:**
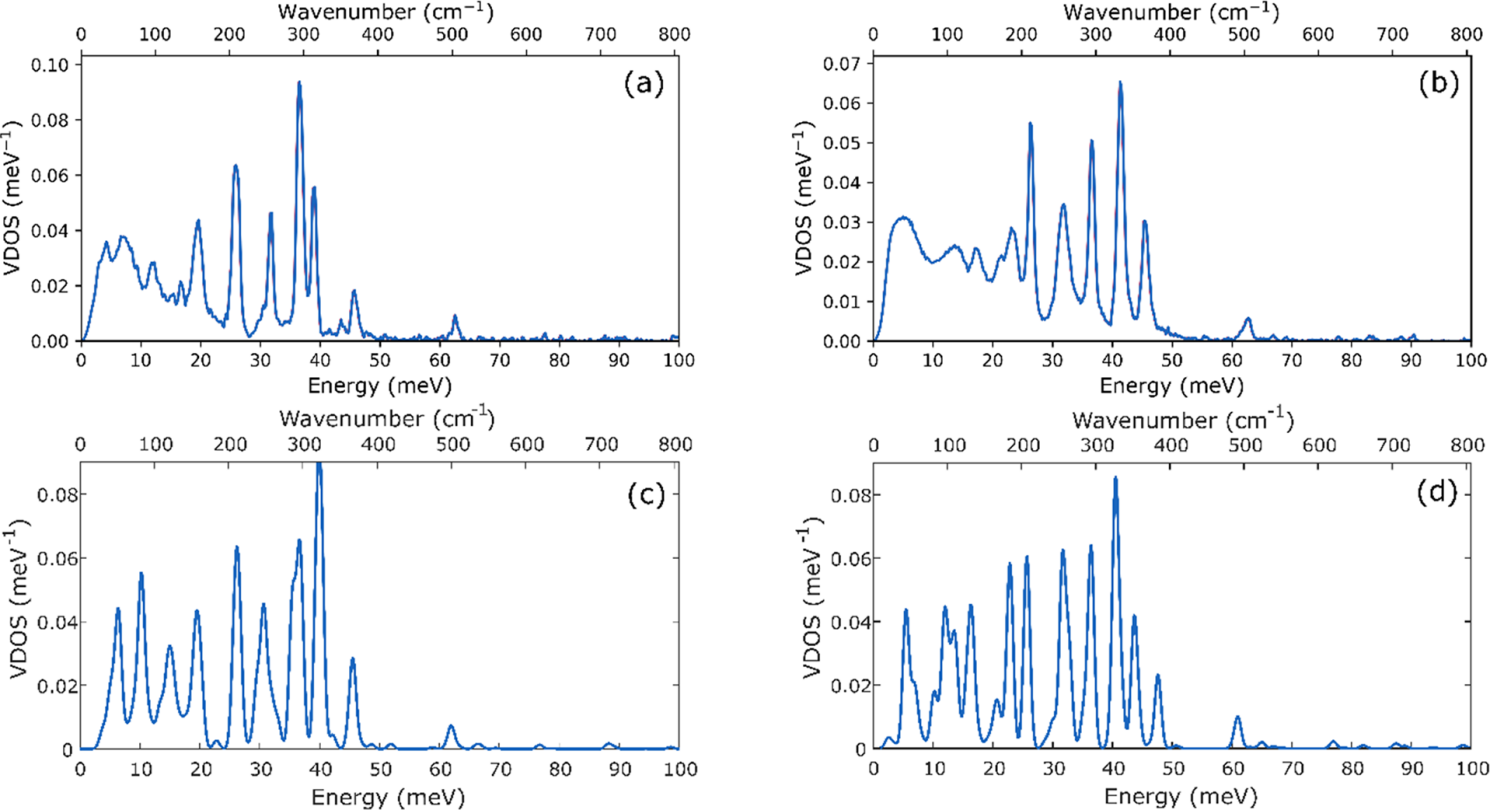
Experimental NRVS spectrum of the solid (L^Me3^FeCl)_2_ (a) and the (L^Me3^FeCl)_2_ precursor in THF (b) along with the calculated spectrum of solid (L^Me3^FeCl)_2_ (c) and of (L^Me3^FeCl)_2_ dissolved in THF (d), which we conclude has the structure L^Me3^Fe(Cl)(THF). Error bars of the experimental NRVS spectra (a, b) are provided in Figure S10.

NRVS spectra of ^57^Fe-labeled solid [L^Me3^FeCl]_2_ and THF solution samples of [L^Me3^FeCl]_2_ are shown in Figure 7a,b. The NRVS calculations utilizing the optimized coordinates of [L^Me3^FeCl]_2_ (multiplicity = 9) are shown in Figure 7c. The strong agreement between theory and experiment indicates that this approach provides an accurate method for assessing the structure. Further, we note that the same calculations also reproduce the experimental Mössbauer isomer shifts for the solid sample (δ_exp_ = 0.93 vs δ_calc_ = 0.87 and 0.79 mm/s). Experimental and calculated quadrupole splittings do not accord as well between experiment and computations, but this is not surprising because Münck has shown that the quadrupole splittings in a closely related system are extremely sensitive to small changes in geometry.^68^ Further, since the calculated quadrupole splittings typically have larger uncertainty than the calculated isomer shifts,^69,70^ the quantitative information that can be obtained from the quadrupole splitting is more limited.

Next, we calculated Mössbauer and NRVS spectra of potential monomeric structures that could be formed in THF. These included [LFeCl_2_]^−^, [LFe(THF)_2_]^+^, and LFe(Cl)(THF); all are high-spin iron(II) with *S* = 2 (multiplicity = 5). The calculated isomer shifts were 0.87, 0.95, and 0.88 mm/s, respectively, which are all within the expected ±0.1 mm/s uncertainty for the calculated Mössbauer isomer shifts. Thus, the isomer shift alone is not sufficient for identifying the nature of the monomeric complex in solution. However, the calculated NRVS spectra (Figure S9) differ more substantially and clearly favor the LFe(Cl)(THF) structure, in agreement with the EXAFS data. This highlights the power of our multipronged spectroscopic and computational approach.

### Structure of the Nitride Product (NP) in THF Solution

The solution structure of NP is also relevant to the process of analyzing the reaction mixtures. The structure in benzene solution is likely to be the same as the one crystallographically characterized, based on the Mössbauer spectrum of 30 mg dissolved in 0.5 mL of benzene, and frozen for measurement at 80 K. This spectrum fits well to three doublets with parameters that agree with those observed for the earlier reported solid-state Mössbauer spectrum of NP. Consistent with this assessment, the reported ^1^H NMR spectrum in C_6_D_6_ is indicative of molecular *C*_2v_ symmetry, as observed in the X-ray crystal structure.^19^ The ^1^H NMR spectrum in C_6_D_12_ is similar (Figure S11).

However, the ^1^H NMR chemical shifts for NP in THF-d_8_ solutions are significantly different, indicating that the structure in THF is not the same as that in the X-ray crystal structure. One of the sets of signals in THF-*d*_8_ has chemical shifts that match with those in L^Me3^FeCl(THF) described above and is thus attributed to the cleavage of K–Cl or K–N bonds that release the dangling iron(II) site. Other paramagnetically shifted signals are observed, of which the clearest are at δ 75, 7.2, −6.8, −14.5, −20, −58, and −68 ppm at room temperature. Though these presumably arise from an Fe_3_N_2_ cluster, their analysis is complicated by the relatively small number of observable peaks. (This situation often arises with paramagnetic complexes because some protons give signals that are broadened into the baseline due to rapid relaxation.) Another complication evident from the NMR studies of NP is that new peaks appear over minutes to hours in THF at room temperature (Figure S12), indicating that the mixture is evolving to another product. This presumably explains why the optimized synthetic procedure for NP in THF requires immediate removal of the THF solvent from the crude material, which is redissolved in noncoordinating hexanes for crystallization; in hexanes or benzene, the neutral Fe_4_K_2_ core is attained. Therefore, subsequent solution experiments used THF solutions that were freshly dissolved.

In order to gain insights into the structure of NP in THF, Mössbauer spectra of a THF solution of NP (33 mM) were analyzed immediately after dissolving. Figure S13 compares the Mössbauer spectrum for NP in the solid to that in THF solutions. The spectrum of the THF solution sample was fit to three peaks, with the intensity ratio constrained to 2:1:1 (Table 1). Although the isomer shifts are similar, one of the smaller components (with δ = 0.90) has a significant increase in its quadrupole splitting from Δ*E*_Q_ = 1.80 to 2.13 mm/s, which is the value observed for L^Me3^Fe(Cl)(THF). We cannot resolve whether there is an interaction of Cl with potassium in solution since DFT computations on LFeCl_2_K(THF)_2_, LFe(Cl)(THF), and LFe(THF)_2_^+^ gave similar computed Mössbauer parameters (see above). However, overall, the Mössbauer spectroscopy agrees with the NMR-based hypothesis that the dangling iron dissociates in solution.

**Table 1:** Experimental Mössbauer Spectral Parameters for NP

NP as a solid			NP in THF		
	I.S. (mm/s)	Δ*E*_Q_ (mm/s)		I.S. (mm/s)	Δ*E*_Q_ (mm/s)
component 1 (50%; Fe^3+^; Fe^3+^ in triiron unit)	0.29	1.79	component 1 (50%; Fe^3+^ in triiron unit)	0.32	1.36
component 2 (25%; Fe^2+^ in triiron unit)	0.68	1.54	component 2 (25%; Fe^2+^ in triiron unit)	0.70	1.54
component 3 (25%; Fe^2+^ dangler)	0.96	1.80	component 3 (25%; Fe^2+^ in THF solution)	0.90	2.13

In order to test these ideas, we constructed a DFT model of the triiron core with a K(THF) unit in place of the K_2_Cl_2_FeL^Me3^ unit from solid-state NP (Figure S14). This model gave calculated isomer shifts of 0.45 (Δ*E*_Q_ = 2.13 mm/s) and 0.57 mm/s (Δ*E*_Q_ = 1.72 mm/s), which are in moderate agreement with the experimental data. An alternative model, in which the iron atom that is three-coordinate in the solid-state structure of NP coordinates THF to become four-coordinate, predicted an isomer shift of 0.72 mm/s that is closer to the experimental value; therefore, this model is also compatible with the data and is slightly favored.

**Figure 8:**
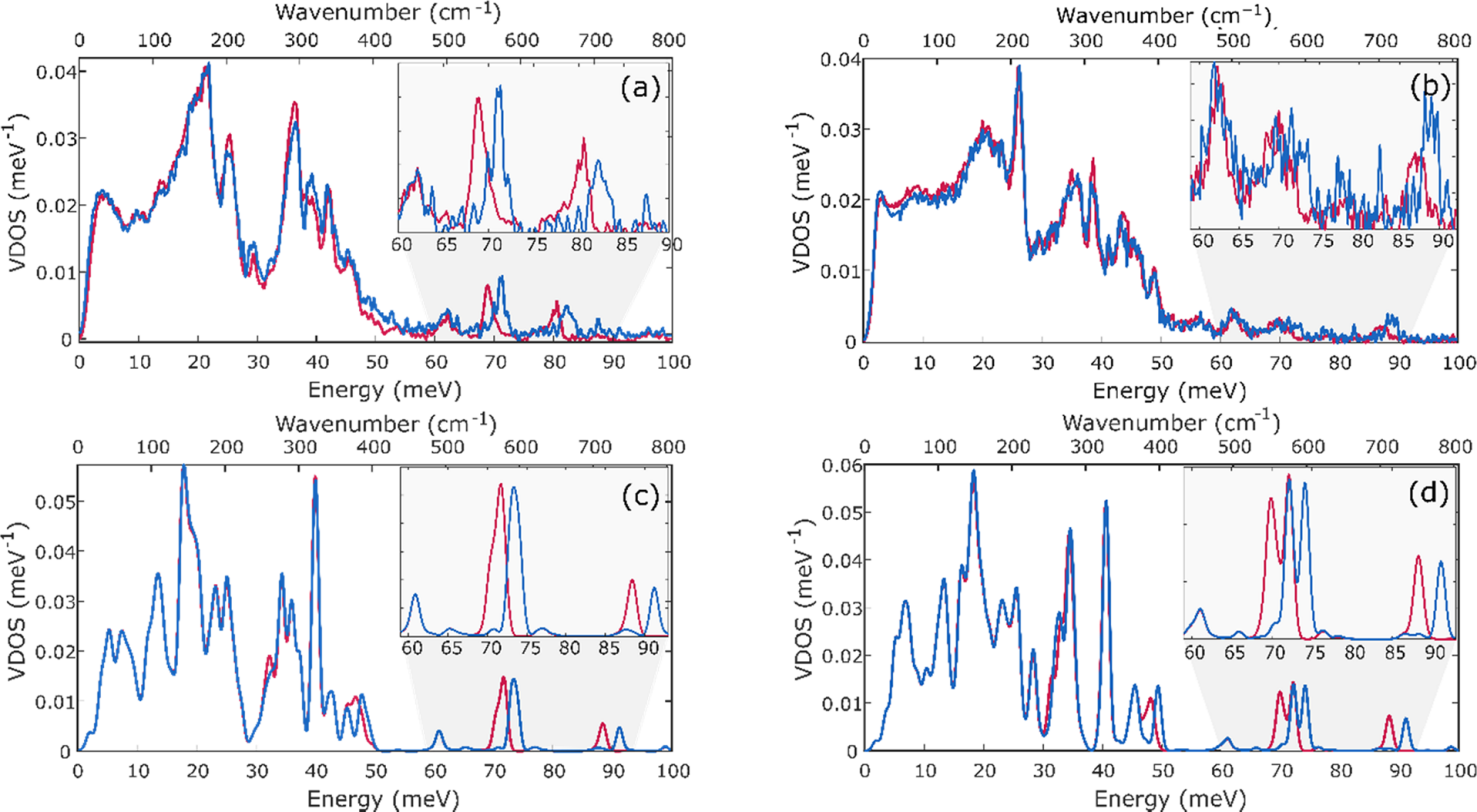
Experimental NRVS spectra of NP (a) and NP in THF (b) along with the calculated spectra of NP (crystallographic structure) (c) and the triiron core with K(THF) (d). Spectra of species with ^14^N are shown in blue and those with ^15^N are shown in red. Error bars of the experimental NRVS spectra (a, b) are provided in Figure S15.

We also measured NRVS spectra of both the solid and frozen THF solution samples of NP with either ^14^N or ^15^N in the nitride bridges. Upon solvation in THF, the bands at 71.3 and 82.4 meV (575 and 665 cm^–1^) (Figure 8a) shift higher to 71.6 and 89.0 meV (578 and 718 cm^–1^), respectively (Figure 8b). Both features are sensitive to N isotope labeling and shift lower by ∼2 meV (∼16 cm^–1^) upon ^15^N substitution (Table 2). The assignments of the observed features again require a correlation to calculations. For this purpose, we used the electronic structures that were already validated by a comparison of the calculated parameters to the Mössbauer spectroscopy results. NRVS spectra were calculated with the incorporation of both ^14^N_2_ and ^15^N_2_ in the bridge, as shown in Figure 8c,d. The calculated spectra agree reasonably well with the experimental trends, and again, as observed with the spectra above, vibrational frequencies are slightly overestimated. The feature at ∼73 meV (∼589 cm^–1^) in the NRVS spectrum of a solid NP which was assigned as a wagging mode of the Fe_3_N_2_ unit is present at 72.2 and 74.1 meV (582 and 598 cm^–1^) in the calculated spectrum of the triiron core with K(THF). The Fe_3_N_2_ asymmetric stretching mode of the trimeric iron unit, which was present in the calculated NRVS spectrum of the solid NP, remained at the same energy in the calculated spectrum of the NP upon solvation in THF.

**Table 2:** ^14^N/^15^N-Labeling Sensitive Bands in NRVS Spectra

	^14^N	^15^N
NP solid (experiment)	71.3 and 82.4 meV (575 and 665 cm^–1^)	68.9 and 80.6 meV (556 and 650 cm^–1^)
NP solid (calculations)	73.4 and 91.2 meV (592 and 736 cm^–1^)	71.7 and 88.4 meV (578 and 713 cm^–1^)
NP in THF (experiment)	71.6 and 89.0 meV (578 and 718 cm^–1^)	70.0 and 87.4 meV (565 and 705 cm^–1^)
NP in THF (calculations)	72.2, 74.1, and 91.1 meV (582, 598, and 735 cm^–1^)	69.9, 72.1, and 88.3 meV (564, 582, and 712 cm^–1^)

These data do not rule out models in which the cluster is further fragmented, but as described in the Introduction, solutions of NP in THF can have the solvent removed, and redissolving in benzene or hexanes gives tetrametallic NP again; this phenomenon would be difficult to reconcile with a greater fragmentation of the core in THF. Therefore, though there are details of the model that are not definitive (e.g., number of THF molecules on the K cation), the agreement of Mössbauer spectra with the NP solid and with DFT models supports the triiron model for NP in the THF solution.

### Intermediates during the N_2_ Cleavage Reaction

Now, we come to the most challenging task, the assessment of possible structures for the intermediate (INT) formed on the way to NP. In the N_2_ reduction reactions, an initial solution of L^Me3^Fe(Cl)(THF) was generated by dissolving [L^Me3^FeCl]_2_ in THF. This solution was frozen, and 2.3 equiv of solid KC_8_ (potassium on graphite, a reducing agent) was added. (We employed a slight excess of KC_8_ beyond the 2 equiv theoretically required to account for the incomplete activity of KC_8_ and potential oxidizing impurities in the solvent or reaction flasks.) As the solution began to melt (near the freezing point of THF at −108 °C), there was an immediate change of color from yellow (characteristic of the iron(II) chloride THF complex) to forest green. When this mixture was warmed to room temperature, the addition of pentane or hexane gave an immediate color change to red, which corresponds to the crystallographically characterized NP. (Addition of hexane could be done to either a THF solution or after removing the THF under vacuum.)^19^ This behavior suggested that the green species is an intermediate on the way to N–N bond cleavage, and accordingly, we refer to it here as INT. We have been unable to crystallize INT for structural characterization, and hence, we proceeded to assess viable structures by combining spectroscopy and DFT calculations.

First, we outline various experiments used to narrow down the possibilities for the potential structures of INT. Initially, we varied the solvent used in the reduction reaction and observed that the green color develops in tetrahydrofuran (THF) and 2-methyltetrahydrofuran (2-Me-THF) but not in 2,5-dimethyltetrahydrofuran. All of these reactions did ultimately give NP, as determined by ^1^H NMR spectroscopy of the product after solvent removal. Since THF, 2-Me-THF, and 2,5-dimethyltetrahydrofuran have similar polarities and differ mainly in their coordinating ability,^71^ we conclude that coordination of THF is important for stabilizing INT enough that it may be observed. Thus, INT is likely to contain coordinated THF.

Addition of benzene to INT, or attempts to generate INT in benzene, generated L^Me3^Fe(C_6_H_6_) as reported previously.^72^ (Performing the reaction in diethyl ether gave neither INT nor NP; the predominant product was [KL^Me3^FeCl_2_]_4_.)^18,72^ The high yields of forming L^Me3^Fe(C_6_H_6_) from INT, as well as products from the addition of S_8_,^73,74^ proceed with stoichiometries which suggest that the average Fe oxidation state in INT is +1. Below, we will consider models in which all iron is in the iron(I) oxidation state as well as models with equimolar amounts of iron(0) and iron(II).

Next, we used a manometry experiment to test whether N_2_ is present in INT. INT was generated and brought to room temperature and immediately treated with 7 or 10 equiv of benzene at a constant *T* of 22 °C and a constant pressure of 1 atm with a manometer attached to measure any increase in the volume of the headspace. This generated L^Me3^Fe(C_6_H_6_), as well as 1 equiv of a gas (which we assume is N_2_) per 4 Fe added. Since this has the same stoichiometry as NP, it suggests that N_2_ has already been incorporated in INT. As an independent test of this idea, we attempted the formation of INT under an atmosphere of Ar. No green color was observed nor were the characteristic peaks for NP observed in the ^1^H NMR spectra. Exposure of this mixture to N_2_ did not lead to any green color, suggesting that N_2_ reacts with an earlier intermediate in a necessary step toward INT (and ultimately NP). Further, treatment of a solution of INT with 12 equiv of H_2_SO_4_, followed by warming, removal from the glovebox, and subjecting the mixture to the indophenol test for ammonia revealed a yield of ammonia of <5%. This contrasts with the high yields of ammonia from the acid treatment of NP,^19,21^ as is typical for compounds in which the N–N bond has been cleaved. These experiments suggest that N_2_ is present in INT, but the N–N bond is intact. Unfortunately, resonance Raman spectra of INT solutions have shown no clear ^15^N-sensitive bands.

**Figure 9:**
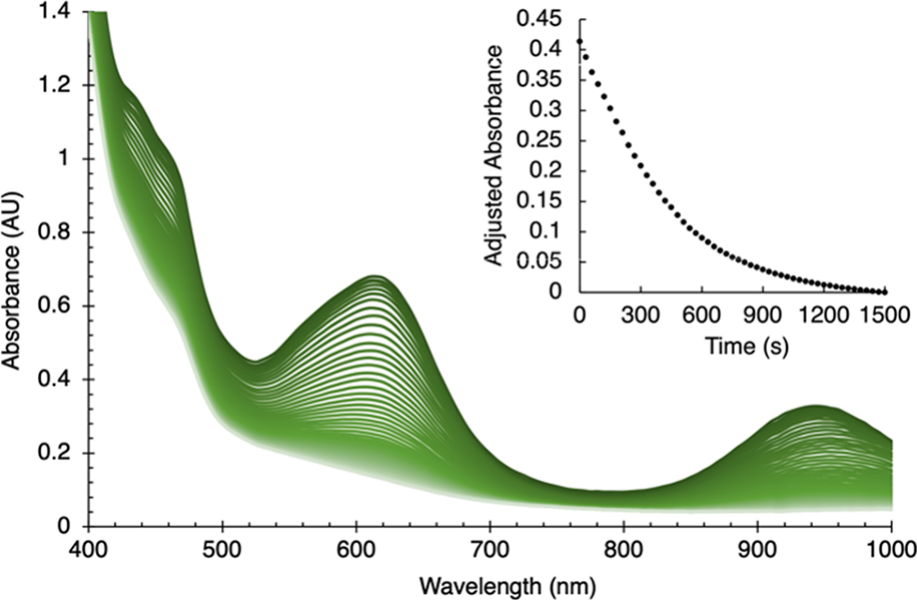
Disappearance of INT over 30 min at room temperature, using UV–vis spectrophotometry. Initial concentration of [L^Me3^FeCl]_2_ = 37 mM; path length = 1 mm; and *T* = 20 °C. Inset: decrease in absorbance at 615 nm during this time (not including the first 5 min while the sample was equilibrating).

In a series of spectroscopic experiments, we generated solutions of INT near −100 °C in a glovebox cold well, as described above, filtered at −80 °C to remove graphite and the remaining KC_8_ and monitored by UV–vis spectrophotometry. INT has distinct features at 615 (ε ≈ 1800 M^–1^ cm^–1^) and 945 nm (ε ≈ 900 M^–1^ cm^–1^) that result in its green color. These features decay upon warming but persist for hours below −30 °C. At room temperature, the loss of the characteristic absorption spectrum is more rapid, and the absorbance at 615 nm follows an exponential decay, with a half-life of about 5 min at 20 °C (Figure 9). We assume that the product corresponds to NP in THF, as both are yellow solutions without distinct maxima in the UV–vis spectrum, but other experiments (NMR and Mössbauer) below are more enlightening in this regard.

**Figure 10:**
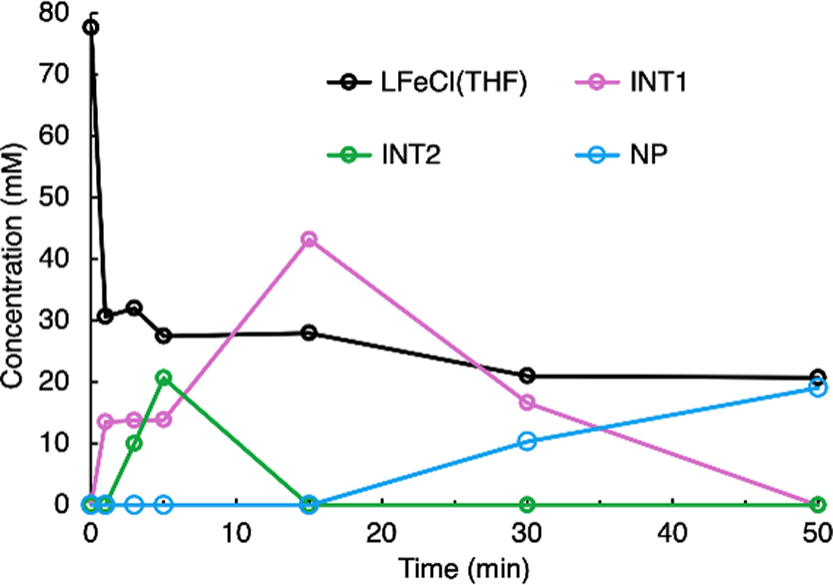
Concentrations of different species over time, from integrations vs a standard in the ^1^H NMR spectra of a sample generated from [L^Me3^FeCl]_2_, KC_8_, N_2_, and THF-*d*_8_ at low temperatures and then held at −50 °C. Spectra are shown in Figure S16.

We also used NMR spectroscopy to monitor the reaction in detail at a lower temperature, and this showed that the situation is more complicated than initially suspected (Figure 10). For these experiments, we generated INT in THF and held it at −50 °C. Aliquots were removed at different time points and cold-filtered before collecting NMR spectra at a low temperature. Peaks are observed for L^Me3^FeCl(THF), as well as two transient species (Figure S16). One transient species (INT1) is present immediately and persists past 30 min, and another transient species (INT2) grows in after 1 min and then disappears by 15 min. At the same time, the peaks from L^Me3^FeCl(THF) shift during the early stages of the reaction, suggesting that they may correspond to a species that is in equilibrium with another paramagnetic complex. Starting around 30 min, a substantial amount of NP begins to form as INT peaks disappear.

In addition, we examined the INT species with Mössbauer spectroscopy at various time points after warming to room temperature (from 0 min when only the precursor is present to 40 min when the conversion to NP is largely complete; see Figure S17). All Mössbauer spectra were fit to a superposition of six quadrupole doublets, the parameters of which are presented in Table 3. Component 1 comes from L^Me3^Fe(Cl)(THF), and components 5 and 6 are characteristic of the triiron cluster in NP. The remaining components are assigned as intermediates (INT1 and INT2).

**Table 3:** Isomer Shift and Quadrupole Splitting of Parameters Used for Fitting the Spectra Collected during the Evolution of INT

	**I.S.**[mm/s]	**ΔEq**[mm/s]	**Assignment**
component 1	0.90	2.13	LFe(Cl)(THF)
component 2	0.80	1.13	INT1
component 3	0.70	0.77	
component 4	0.46	2.28	INT
component 5	0.32	1.36	NP in THF
component 6	0.70	1.54	

**Figure 11:**
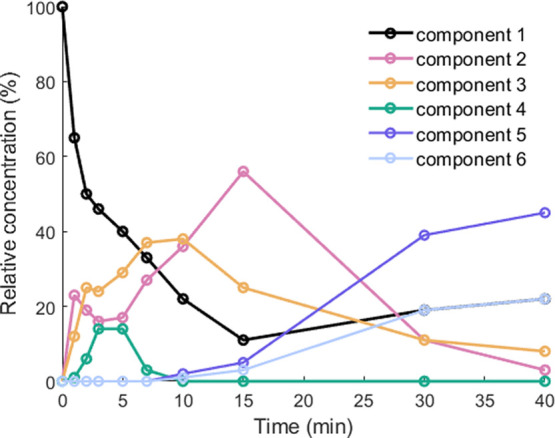
Variation of the relative concentrations of the components in Mössbauer spectra fits over the course of the reaction. The components in the figure correspond to those given in Table 1.

Variations of the relative concentrations of the components over the course of the reaction, as judged by Mössbauer spectroscopy, are shown in Figure 11. The time course plots from NMR and Mössbauer spectroscopies are similar (despite the different temperatures used in the experiments), suggesting that the NMR-identified INT1 corresponds to component 2 in the Mössbauer spectra, while INT2 from the NMR spectra corresponds to component 4 in the Mössbauer spectra. Component 3 in the Mössbauer spectra is apparently not discernible in the NMR spectra and may be NMR-silent or is in rapid exchange with L^Me3^FeCl(THF) on the NMR timescale.

**Figure 12:**
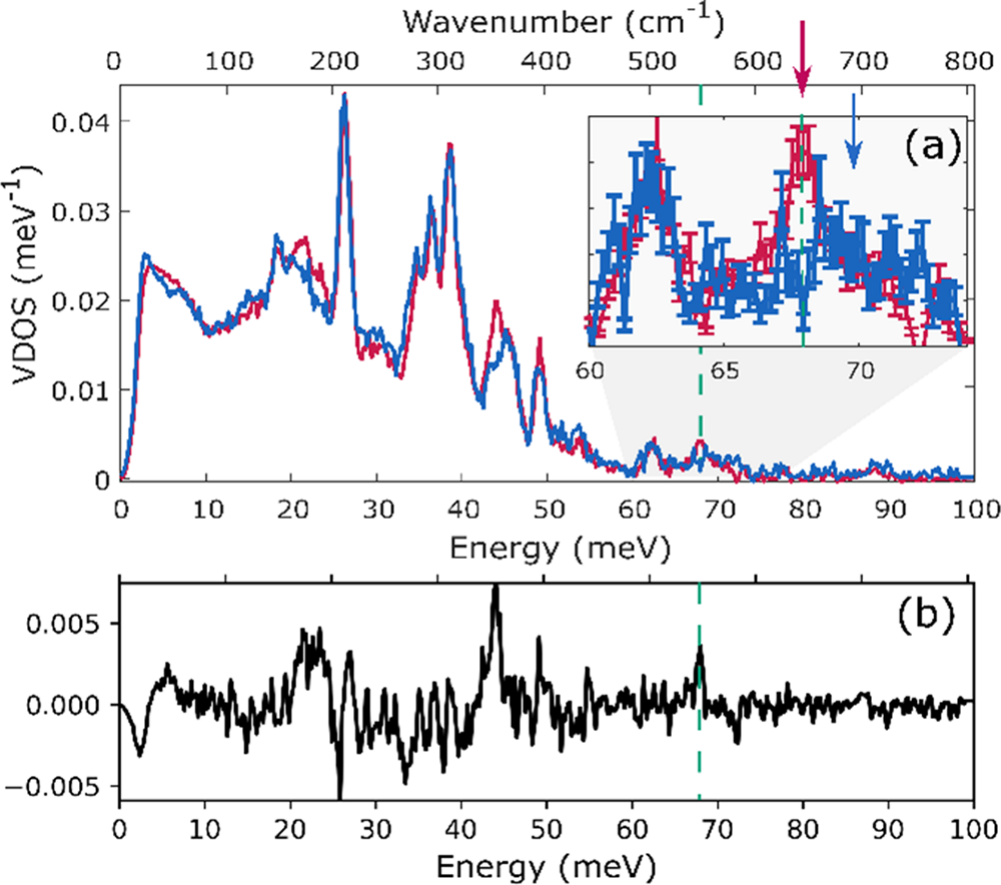
Comparison of ^14^N- and ^15^N-labeled NRVS spectra of the solution after 3 min of the reaction (a). Spectrum of the compound with bridging ^14^N_2_ is shown in blue and that with bridging ^15^N_2_ is shown in red. For a better visualization of changes due to labeling, the difference spectrum was plotted (b). The inset shows the changes in the experimental NRVS spectrum with error bars included to give a clearer view of the significance of the peaks. A full spectrum of (a) with error bars is shown in Figure S18.

NRVS spectra were also measured for very early time points with either ^14^N- or ^15^N-labeled N_2_. Figure 12 shows the comparison of the ^14^N- and ^15^N-labeled reaction mixture after 3 min, a time at which INT1 and INT2 are present. Differences are seen at ∼40 meV (322 cm^–1^) and ∼68 meV (548 cm^–1^), indicating that N_2_ has already bound to iron at this stage of the reaction (consistent with the manometry experiments above).

**Figure 13:**
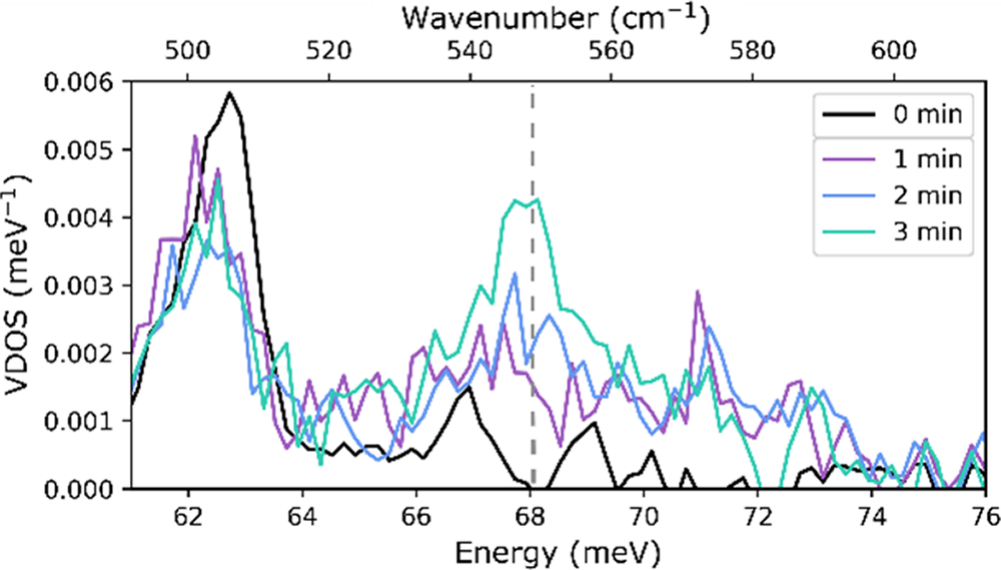
Zoom of the NRVS spectra measured 0, 1, 2, and 3 min after mixing the components to form INT. Error bars of the experimental NRVS spectra are shown in Figure S19.

Since our calculations in the previous section demonstrate that the high-energy vibrations are generally more reliably calculated than the low-energy vibrations, our attempts to identify the nature of the green intermediate focused on the peak at 68 meV (548 cm^–1^) that is well isolated and sensitive to ^15^N labeling. NRVS spectra measured after 0, 1, 2, and 3 min of the reaction show an increase in the intensity of the peak at 68 meV (548 cm^–1^) (Figure 13). Thus, it is likely to be associated with INT2 (component 4).

**Figure 14:**
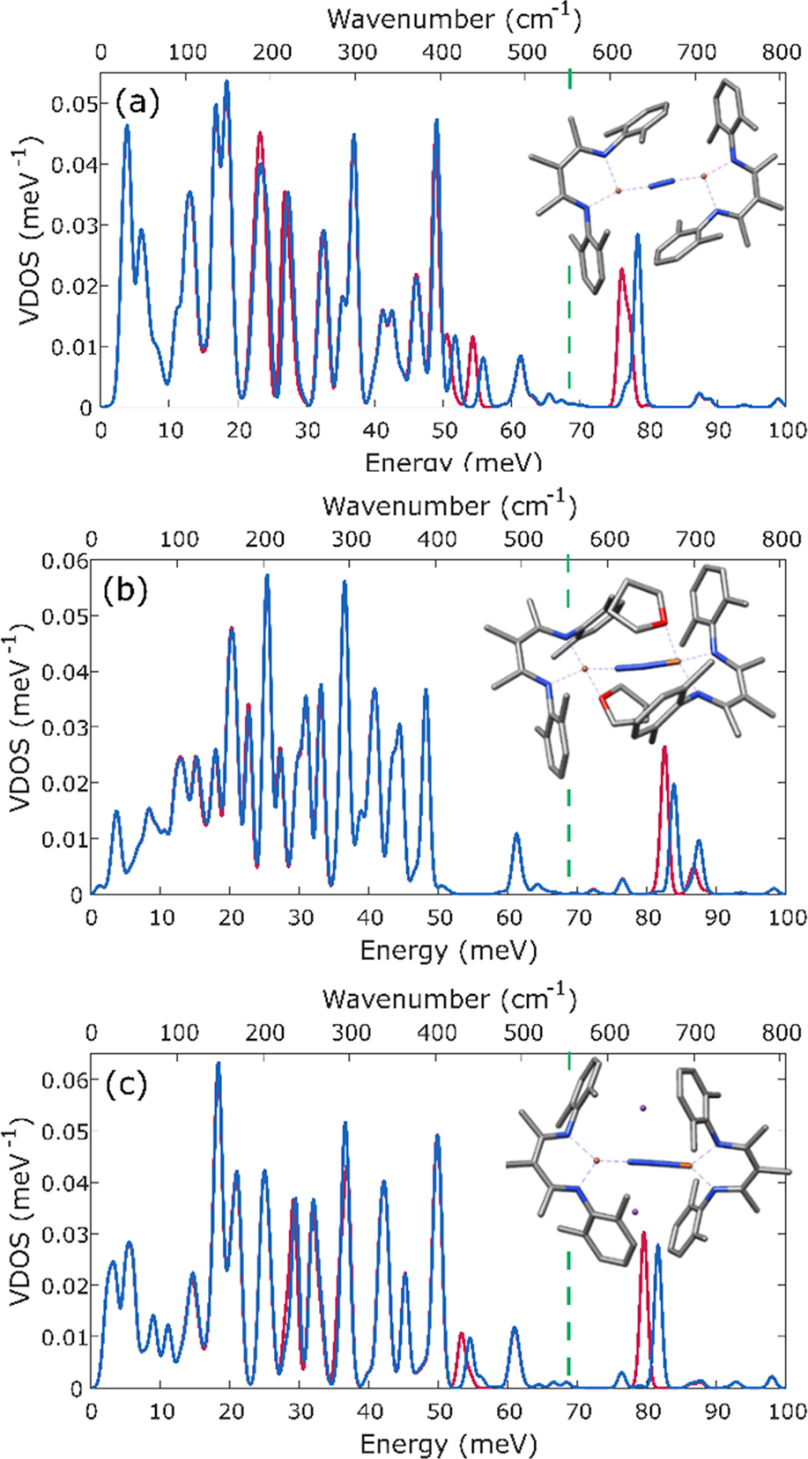
Calculated NRVS spectra of LFeNNFeL (a), LFe(THF)NNFe(THF)L (b), and K_2_LFeNNFeL (c). In each case, the green dashed line denotes the experimental energy of the experimental band (INT2).

By the correlation of these observations with the Mössbauer data presented in the previous section, it is most likely that INT2 (formed in the early minutes of the reaction) corresponds to the Mössbauer parameters of δ = 0.46 and Δ*E*_Q_ = 2.28 mm/s and an ^15^N-sensitive peak in the NRVS spectrum at 68 meV (548 cm^–1^). Utilizing this information, we then constructed DFT models of monometallic and bimetallic species that are possible structures of intermediates (Figures 14 and S20). We calculated the Mössbauer spectra as well as NRVS for ^14^N and ^15^N isotopologues. In mononuclear models (Figure S20) with end-on N_2_, the N_2_ isotope-sensitive modes are far too low in energy (<53 meV/427 cm^–1^) relative to experiment (68 meV/548 cm^–1^). On this basis, the calculated mononuclear models are ruled out. In bimetallic models (Figure 14), three representative species were tested: formally diiron(I) LFeNNFeL in which the irons remain three-coordinate, the related LFe(THF)NNFe(THF)L in which each iron also has a THF coordinated, and finally the formally diiron(0) K_2_LFeNNFeL (Figure 14). In all cases, N_2_ isotope-sensitive bands are predicted at higher frequencies (78–84 meV/629–678 cm^–1^) that are closer to experiment. Considering that the calculations generally overestimated the energies of these vibrational bands in the known compounds above (on average by Δ*E* = 3.1 meV (25 cm^–1^), with up to 8.1 meV (65 cm^–1^) for K_2_L^*t*Bu^FeNNFeL^*t*Bu^), N_2_-bridged dimers seem to be good candidates for INT species.

The calculated Mössbauer spectra of the three bimetallic models were different: the predicted isomer shifts for L^Me3^FeNNFeL^Me^ (0.54 mm/s) and K_2_L^Me3^FeNNFeL^Me3^ (0.42 mm/s) were consistent with INT2. THF coordination to the Fe centers led to a higher isomer shift of 0.80 mm/s, which corresponds to component 2 (INT1) observed by Mössbauer spectroscopy. Perhaps, most convincingly, the known molecule K_2_L^*t*Bu^FeNNFeL^*t*Bu^ has Mössbauer and NRVS data (described above) that are very similar to those of INT2 (component 4 in Mössbauer spectra), as well as a deep green color that is similar to that observed for INT2. Thus, we suggest that K_2_L^Me3^FeNNFeL ^Me3^ is a reasonable core structure for the structure of INT2. Additional DFT models having different numbers of THF molecules on the K ions gave very similar predicted Mössbauer and NRVS spectra, and therefore, our data are unable to distinguish them. THF coordination to Fe, on the other hand, gives a higher calculated isomer shift that corresponds to a species that is observed later during the reaction.

Since the average valence of INT is iron(I) (shown from its reactivity and from the amount of KC_8_ added), the formally iron(0) compound K_2_L^Me3^FeNNFeL ^Me3^ could be produced in a maximum of 50% yield, and the other half of the iron would remain iron(II). This is consistent with the observation of a species resembling L^Me3^Fe^II^(Cl)(THF) throughout the course of the reaction. Interestingly, the chloride in L^Me3^Fe(Cl)(THF) has the potential to bridge to other metals, and, for example, we have previously isolated L^Me3^FeCl_2_K(18-crown-6).^19^ Reversible coordination of L^Me3^Fe(Cl) units to the potassium ions in K_2_L^Me3^FeNNFeL^Me3^ could explain the shifting of the apparent L^Me3^Fe(Cl)(THF) peaks in the ^1^H NMR spectra; in this case, there would be a connection between the Fe^0^NNFe^0^ unit and the iron(II) chloride species.

**Scheme 2:**
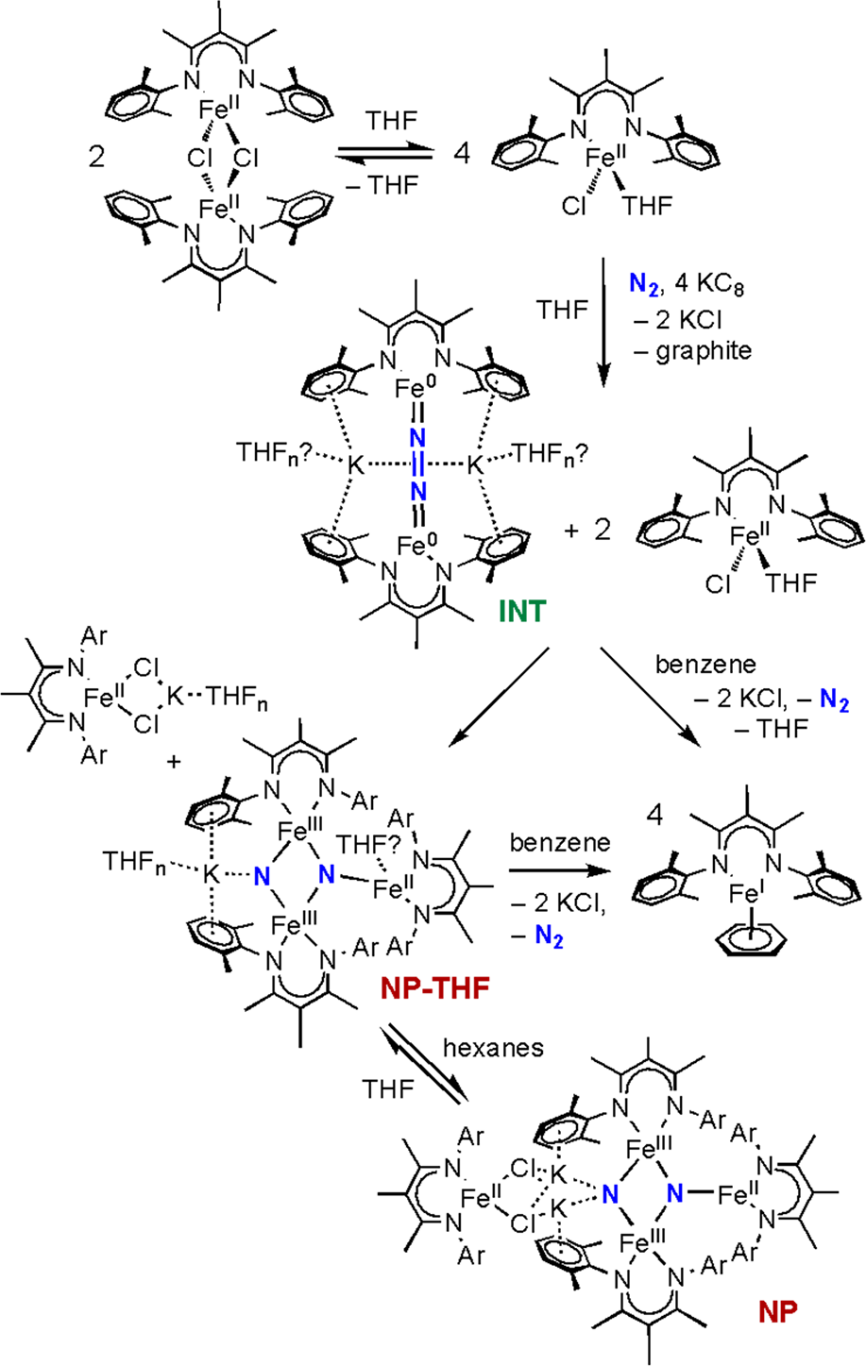
Proposed Mechanism of Forming NP that is Consistent with the Spectroscopic Observations

Overall, the structures of the INT species are not uniquely specified by the data, but the accumulated stoichiometry, manometry, solvent dependence, UV–vis, NMR, Mössbauer, NRVS, and computational evidence fit a self-consistent model (Scheme 2). In this model, addition of 1 equiv of KC_8_ per Fe gives double reduction of half of the iron centers to generate the formally diiron(0) complex K_2_L^Me3^FeNNFeL^Me3^, which has various numbers of solvent THF molecules coordinated to the K and Fe ions in equilibrium, and these close relatives correspond to the spectroscopically observed INT species. Since half of the iron sites remain as iron(II) in LFeCl(THF), addition of benzene can give comproportionation to the iron(I) complex L^Me3^Fe(C_6_H_6_) in accordance with the average iron(I) oxidation level. The mixture of iron(0) and iron(II) components is unstable and can convert to NP in its THF-dissolved form (which has the dangling iron dissociated). The transformation of INT to NP could be dependent on the variations in the location of potassium ions and coordination of THF and chlorides, of which two particularly stable forms are INT1 and INT2 observed by NMR and Mössbauer spectroscopies. The difference between INT1 and INT2 structures is not clear, and thus Scheme 2 contains one suggested structure as INT. This conformational variability could influence the ability of a third iron to approach the FeNNFe unit, and the previous computational study on truncated models^75^ indicates that trimetallic structures can accomplish the cleavage of the N–N bond. These trimetallic species are not observed here, presumably because their formation is rapidly followed by conversion to NP.

Finally, the standard workup for synthesizing NP involves evaporating the THF solvent and redissolving it in hexanes, giving a change to the red solution that is characteristic of the four-iron cluster. This arises because, in the nonpolar solvent without THF, the dangling iron is no longer present as LFeCl(THF): rather, this last iron(II) species binds to the triiron core to form the crystallographically observed four-iron cluster.

## Conclusions

Herein, we utilized NRVS in order to establish spectroscopic fingerprints for iron–N_2_ complexes with weakened and with cleaved N–N bonds. In both cases, through the use of ^14^N/^15^N labeling, we have identified well-isolated high-frequency bands (>70 meV/565 cm^–1^) in the NRVS spectra which are associated with iron–N_2_ stretches. For L^*t*Bu^FeNNFeL^*t*Bu^ and K_2_L^*t*Bu^FeNNFeL^*t*Bu^, the well isolated modes correspond to asymmetric Fe–N=N–Fe core stretches, while for the solid NP, these high-frequency modes are derived from asymmetric Fe_3_N_2_ core stretching modes. The experimentally observed frequencies were reproduced computationally and provided a basis for extending this approach to solution structures in THF. NRVS studies of NP in THF show that a reasonable agreement between experiment and theory can be obtained by assuming an Fe_3_N_2_ trimeric unit, suggesting that this can serve as a robust structural analysis tool.

These findings then allowed the extension of our approach to assess the structure of intermediate species that form in THF solution en route to NP. The complexity in this process starts immediately, as dissolving the precursor [L^Me3^FeCl]_2_ in THF results in a change in spectral properties that indicates a change in structure. Through a combination of Mössbauer, XES, XAS, and NRVS analyses, we confidently assign the precursor in THF as a L^Me3^Fe(Cl)(THF) species. We then followed the low-temperature reaction of this species with a reductant and N_2_ using NMR, Mössbauer, and NRVS of samples at different time points. Several species are present during the reaction sequence, which were difficult to deconvolute. However, using the combination of spectroscopy and comparison with the spectroscopically validated DFT models for potential intermediate species, we identified likely diiron intermediates. Specifically, a K_2_L^Me3^FeNNFeL^Me3^ core structure is implicated, though the precise structures are not known and variable numbers of THF molecules may be coordinated. Even though all the details were not revealed, the experiments described here serve as a demonstration of the insight that can come from this multitechnique approach.

Further, the observation of bimetallic intermediates during N–N cleavage has some mechanistic significance. Even though the INT species are transient, the fact that they can be observed suggests that they are more stable than subsequent intermediates that continue on to form NP more rapidly than they are formed. This logic suggests that the bimetallic N_2_ complexes have relatively low energy relative to the unobserved trimetallic N_2_ complexes. This conceptual model is consistent with the isolability of the analogous bimetallic N_2_ complexes with bulkier ligands, which do not split the bridging N_2_ ligand because the size of the ligand prohibits the formation of trimetallic species.^18^ It also agrees with computations on truncated models, which indicate that there is a facile pathway from a trimetallic Fe_3_ dinitrogen complex to a core with N_2_ split to two nitrides.^75^ Such conclusions should be understood with the caveat that our conceptual model and assignment of INT structures are largely based on this earlier work, and so there is a risk of circular reasoning. However, the ability to explain the accumulated analytical, spectroscopic, and computational results here within the model serves as a fairly stringent test of this model.

## Supporting Information Available

Additional spectra and computational results (PDF)
Selected normal modes (MOV)

## Notes

We thank the National Institutes of Health (GM065313) and the Max Planck Society for funding.
