# Supplementary material for: Mössbauer and Nuclear Resonance Vibrational Spectroscopy Studies of Iron Species Involved in N–N Bond Cleavage
Source: Inorg Chem. 2023 Oct 30;62(45):18449–64. doi: 10.1021/acs.inorgchem.3c02594 (PMC10647920; doi:10.1021/acs.inorgchem.3c02594)
Supplement: Supplementary file 1 — ic3c02594_si_001.pdf [file ic3c02594_si_001.pdf]

Supporting Information

Mössbauer and Nuclear Resonance Vibrational Spectroscopy Studies of Iron Species Involved in N–N Bond Cleavage

Aleksandra Wandzilak,[a,b] Katarzyna Grubel,[c] Kazimer L. Skubi,[c,d] Sean F. McWilliams,[c] Dimitrios Bessas,[e] Atanu Rana,[a,f] Stefan Hugenbruch,[a] Abhishek Dey,[f] Patrick L. Holland,[c] Serena DeBeer [a]**

[a] Max Planck Institute for Chemical Energy Conversion, Mülheim an der Ruhr, Germany

[b] Faculty of Physics and Applied Computer Science, AGH University of Science and Technology, Krakow, Poland

[c] Department of Chemistry, Yale University, New Haven, Connecticut, USA

[d] Department of Chemistry, Carleton College, Northfield, MN 55057, USA

[e] European Synchrotron Radiation Facility, F-38043 Grenoble, France

[f] School of Chemical Science, Indian Association for the Cultivation of Science, Kolkata 700032, India

Table of Contents

1. Reference Samples $L^{tBu}FeNNFeL^{tBu}$ and $K_2L^{tBu}FeNNFeL^{tBu}$ 2
2. Solution structure of $[L^{Me3}FeCl]_2$ 5
3. Structure of the nitride product (NP) in solution 8
4. Intermediates during the N_2 cleavage reaction 11
5. Summary of Mössbauer parameters 16
6. Animations of normal modes of $L^{tBu}FeNNFeL^{tBu}$ and NP 17
7. Optimized Geometries 17

1. Reference Samples $L^{tBu}FeNNFeL^{tBu}$ and $K_2L^{tBu}FeNNFeL^{tBu}$

- Experimental NRVS spectra of $L^{tBu}FeNNFeL^{tBu}$ and $K_2L^{tBu}FeNNFeL^{tBu}$ with error bars

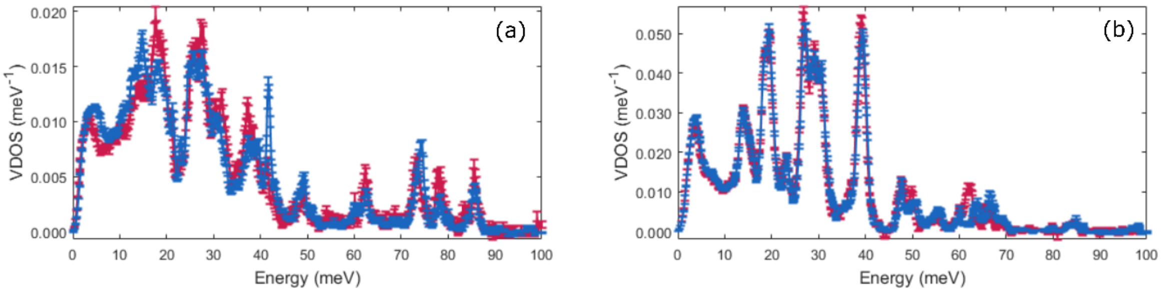

Figure S1. Experimental NRVS spectra of solid $L^{tBu}FeNNFeL^{tBu}$ (a) and K_2 $L^{tBu}FeNNFeL^{tBu}$ (b) with error bars generated by the DOS fitting program.

- Experimental Mössbauer spectrum of $L^{tBu}FeNNFeL^{tBu}$

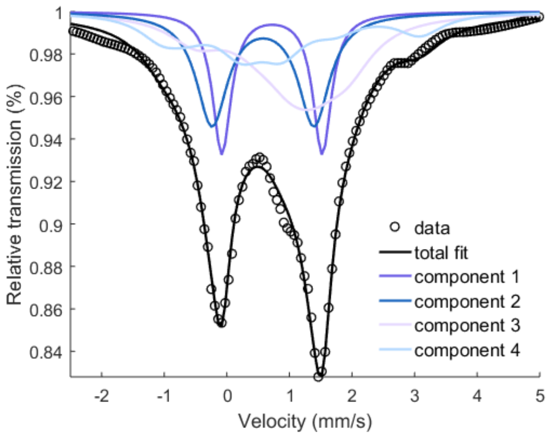

Figure S2. Experimental Mössbauer spectrum of $L^{tBu}FeNNFeL^{tBu}$. The pale components correspond to sextets arising from hyperfine interactions that appear even without applied field (analogous to Stoian et al., J. Am. Chem. Soc. ***2006****, 128, 10181-10192).*

.

Table S1. Experimental and computational Mössbauer parameters of $L^{tBu}FeNNFeL^{tBu}$. We assume that some asymmetric effect from the crystal packing leads to the observation of inequivalent iron sites.

	I.S. (mm/s)	ΔEq (mm/s)	Area
Experimental	0.61	1.63	26 %
	0.73	1.61	20 %
Calculated	0.67	1.55	

- Experimental Mössbauer spectrum of $K_2L^{tBu}FeNNFeL^{tBu}$

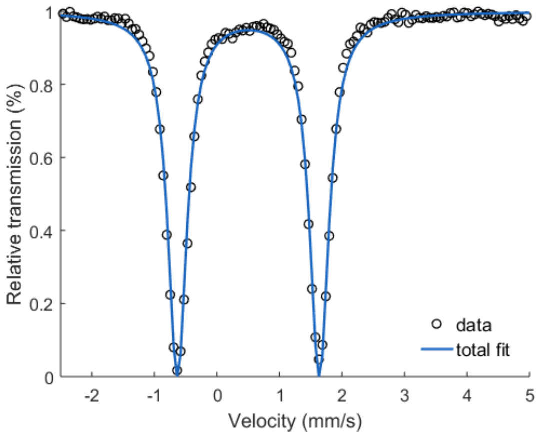

Figure S3. Experimental Mössbauer spectrum of $K_2L^{tBu}FeNNFeL^{tBu}$.

Table S2. Experimental and computational Mössbauer parameters of $K_2L^{tBu}FeNNFeL^{tBu}$.

	I.S. (mm/s)	ΔEq (mm/s)
Experimental	0.50	2.27
Calculated	0.50	2.19

- Resonance Raman spectroscopy of $L^{Me}FeNNFeL^{Me}$

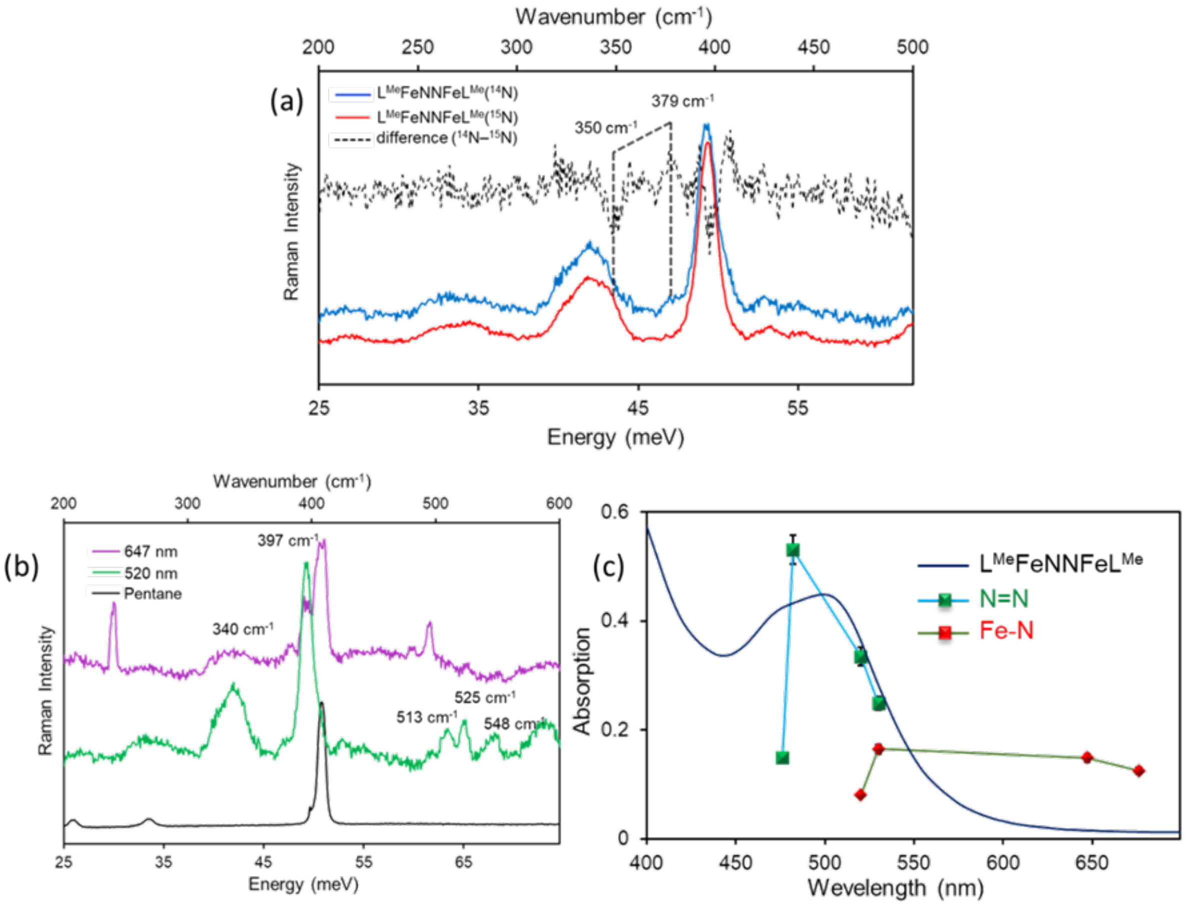

Figure S4a shows resonance Raman spectra of a frozen solution of $L^{Me}FeNNFeL^{Me}$ (520 nm, 77 K, 50 mW) (blue) and its ^{15}N substituted analogue (red). The difference spectrum is shown in black. Apart from the isotope-sensitive 379 (350) cm^{-1} an additional difference has been observed at 403 (395) cm^{-1} in the difference spectrum, which falls on the envelope of pentane solvent. This might be an artefact in this spectrum. Figure S4b compares the spectra in the Fe–N region between excitation energies. Figure S4c shows the excitation profile for $L^{Me}FeNNFeL^{Me}$. The errors on the absorption axis are estimated at ~5%.

- UV-vis spectrum of $K_2L^{Me}FeNNFeL^{Me}$

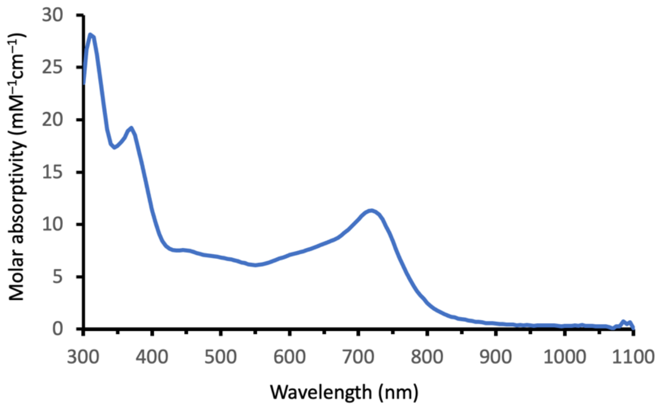

Figure S5. UV-vis spectrum of $K_2L^{Me}FeNNFeL^{Me}$ in pentane.

2. Solution structure of $[L^{Me3}FeCl]_2$

- ^{1}H NMR spectra of $[L^{Me3}FeCl]_2$

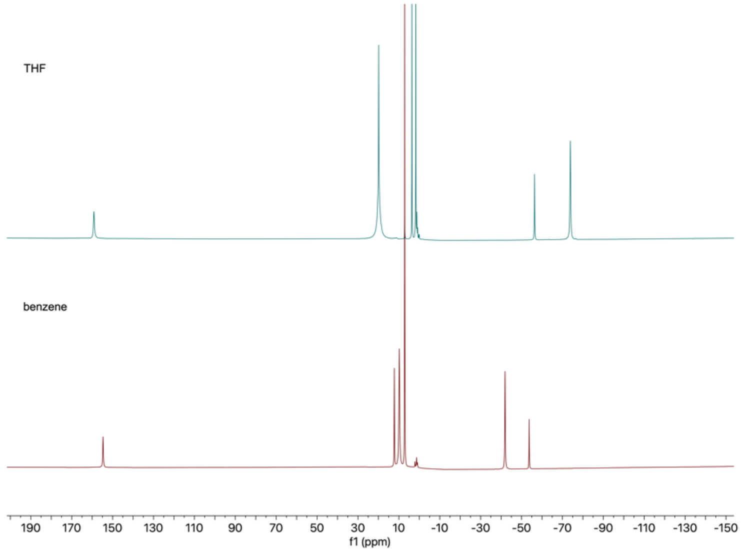

Figure S6. ^{1}H NMR spectra of $[L^{Me3}FeCl]_2$ in THF and in benzene.

- Experimental Mössbauer spectrum of $[L^{Me3}FeCl]_2$ as a solid and in THF.

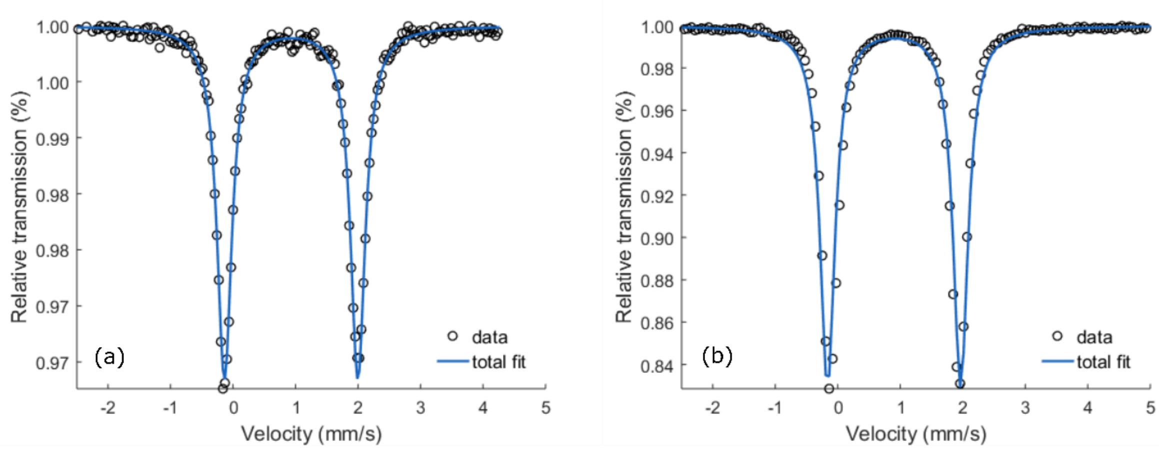

Figure S7. Experimental Mössbauer spectra of solid precursor (a) and the precursor in THF (b)

Table S3. Experimental Mössbauer parameters of of $[L^{Me3}FeCl]_2$.

precursor as a solid

	I.S. (mm/s)	ΔEq (mm/s)
component 1	0.93	2.14

precursor in THF

	I.S. (mm/s)	ΔEq (mm/s)
component 1	0.90	2.13

- EXAFS fit of $[L^{Me3}FeCl]_2$ as solid and as THF solution.

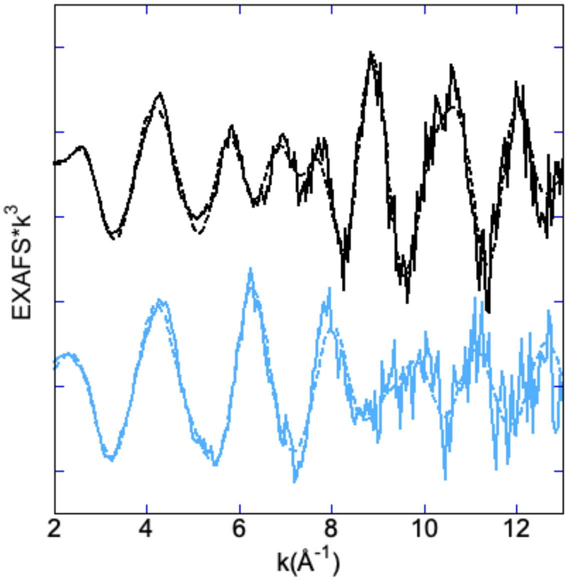

Figure S8. k^3-weighted EXAFS data for solid $[L^{Me3}FeCl]_2$ (black curves, top) and as a solution in THF (blue curves, bottom). In both cases the data are shown as solid lines and the corresponding fits as dotted lines. Fit 3 from Table S4 and fit 3 from Table S5 are show for the solid and solution data, respectively.

Table S4. Selected EXAFS fitting parameters for solid $[L^{Me3}FeCl]_2$. N is the path degeneracy, R distance, σ^2 is the Debye-Waller factor, and delta-E_0 is the shift in the edge energy relative to a E_0 value of 7118 eV. Fit 3 is displayed in the fits.

Fit	Path	N	R (Å)	σ^2 (Å^2)	⊗E_0 (eV)	R factor
1	Fe-N	2	1.98	0.0012	4.12	0.211
	Fe-Cl	2	2.40	0.0071		
2	Fe-N	2	1.94	0.0038	1.02	0.111
	Fe-Cl	2	2.38	0.0054		
	Fe-C	4	2.70	0.0465		
3	Fe-N	2	1.95	0.0010	3.32	0.036
	Fe-Cl	2	2.38	0.0029		
	Fe-C	4	2.94	0.0033		
	Fe-Fe	1	3.34	0.0013		

Table S5. Selected EXAFS fitting parameters for $[L^{Me3}FeCl]_2$ as a solution in THF. N is the path degeneracy, R distance, σ^2 is the Debye-Waller factor, and delta-E_0 is the shift in the edge energy relative to a E_0 value of 7118 eV. Fit 3 is displayed in the fits.

Fit	Path	N	R (Å)	σ^2 (Å^2)	⊗E_0 (eV)	R factor
1	Fe-N	2	1.98	0.0042	-0.037	0.070
	Fe-Cl	1	2.25	0.0072		
2	Fe-N/O	3	2.00	0.0042	1.90	0.064
	Fe-Cl	1	2.26	0.0072		
3	Fe-N/O	3	1.99	0.0045	0.43	0.047
	Fe-Cl	1	2.25	0.0063		

	Fe-C	4	2.99	0.0040		
4	Fe-N/O	3	2.00	0.0045	0.54	0.041
	Fe-Cl	1	2.26	0.0063		
	Fe-C	4	2.99	0.0040		
	Fe-Fe	1	3.63	0.0090		

- **Comparison of the experimental NRVS spectrum of ($L^{tBu}FeCl)_2$ dissolved in THF and calculated NRVS spectra of its potential structures**

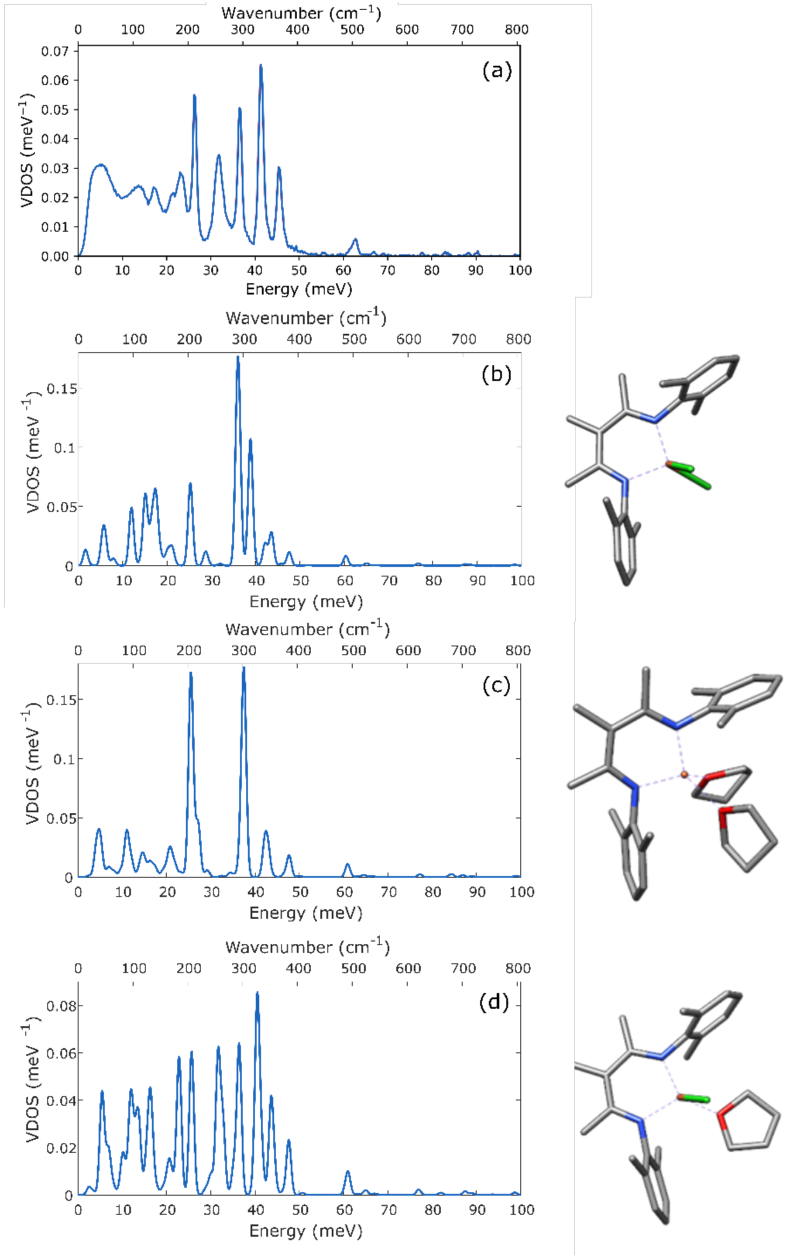

Figure S9. Experimental NRVS spectrum of $[L^{Me3}FeCl]_2$ dissolved in THF (a) and calculated NRVS spectra of its potential structures: $[L^{Me3}FeCl_2]^-$ (b) $[L^{Me3}FeTHF_2]^+$ (c) and $L^{Me3}Fe(Cl)(THF)$ (d).

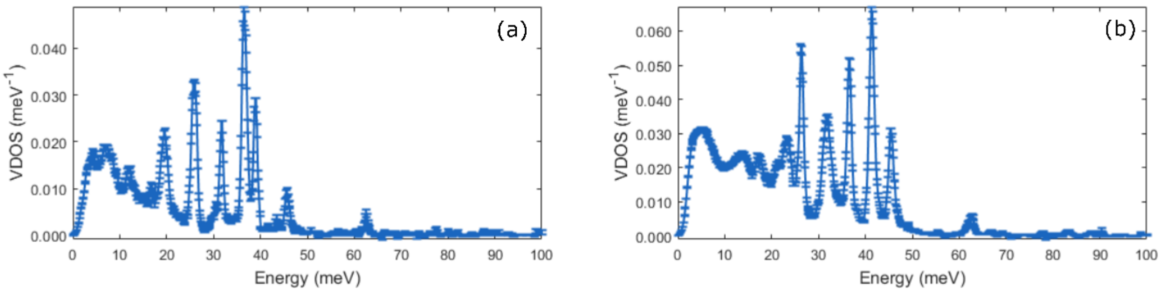

Figure S10. Experimental NRVS spectra of solid $(L^{Me3}FeCl)_2$ (a) and the $(L^{Me3}FeCl)_2$ precursor in THF (b) with error bars generated by the DOS fitting program.

3. Structure of the nitride product (NP) in solution

- ^{1}H NMR spectra of tetrairon complex NP

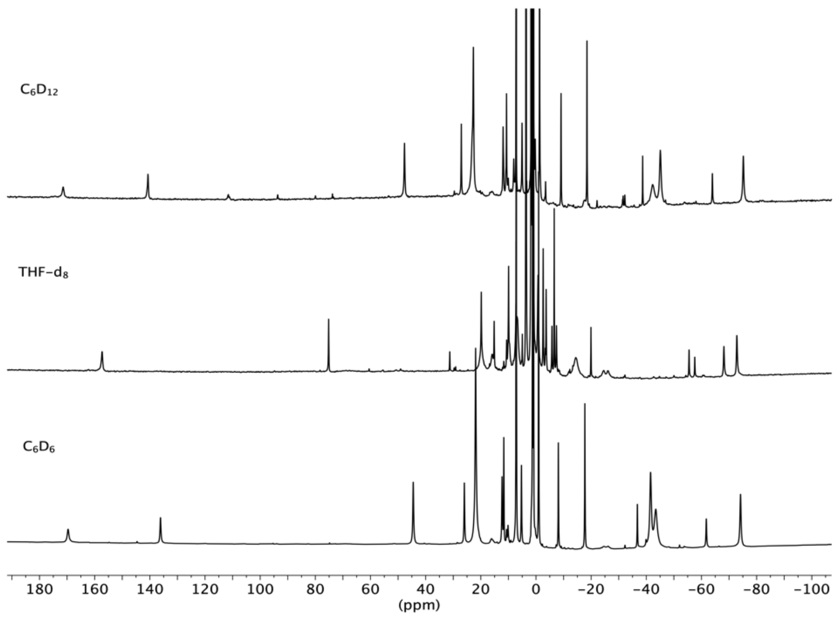

Figure S11. Comparison of ^{1}H NMR spectra of NP dissolved in different solvents.

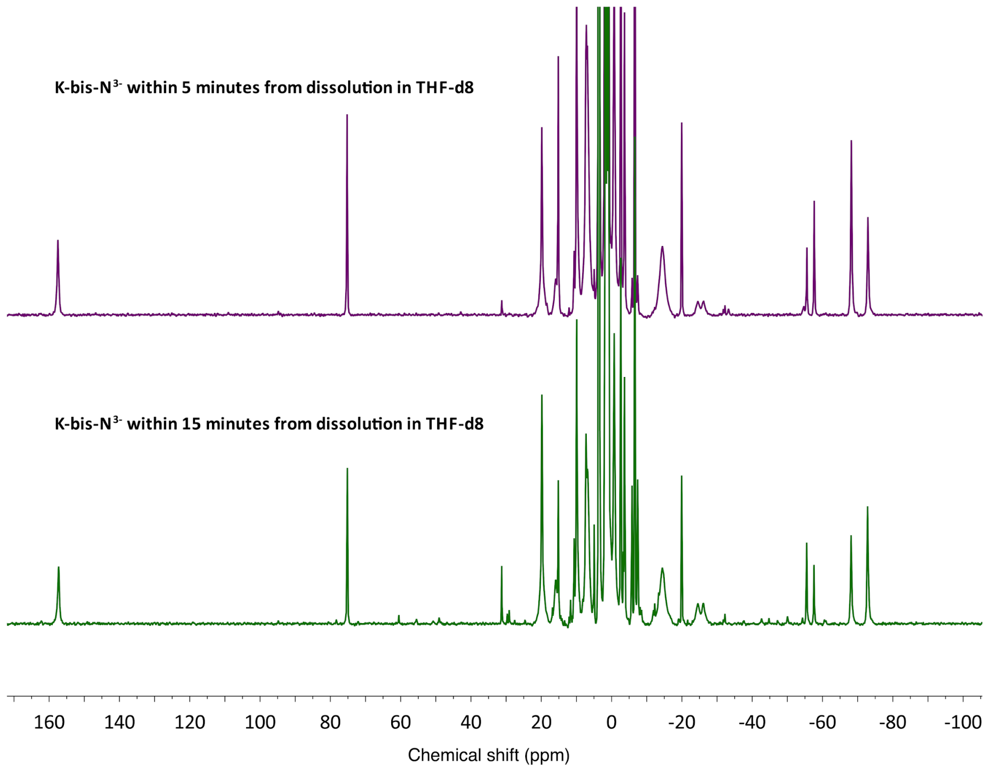

Figure S12. Changes in the ^1H NMR spectrum of NP in THF-d_8 solution.

- Experimental Mössbauer spectra of tetrairon complex NP as a solid and in THF.

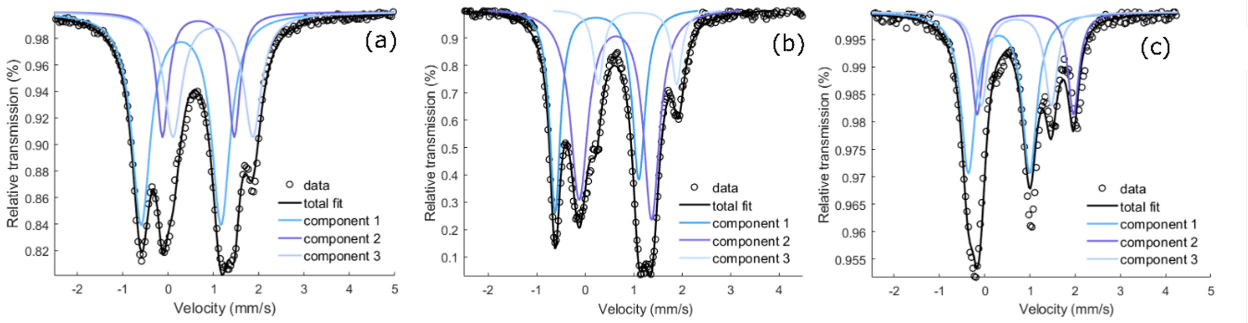

Figure S13. Experimental Mössbauer spectrum of NP as (a) solid (b) benzene solution (c) THF solution.

Table S6. Experimental Mössbauer parameters of NP.

	NP as a solid		NP as a solution in benzene		NP as a solution in THF	
	I.S. (mm/s)	ΔE_Q (mm/s)	I.S. (mm/s)	ΔE_Q (mm/s)	I.S. (mm/s)	ΔE_Q (mm/s)
component 1	0.29	1.79	0.25	1.73	0.32	1.36
component 2	0.68	1.54	0.63	1.50	0.70	1.54
component 3	0.96	1.80	1.08	1.70	0.90	2.13

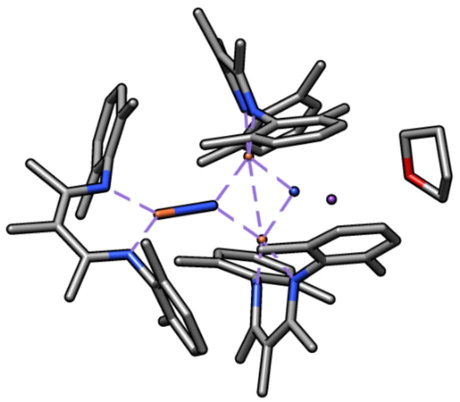

Figure S14. Optimized DFT model of NP in THF.

- NRVS spectra of NP as a solid and in THF solution with error bars

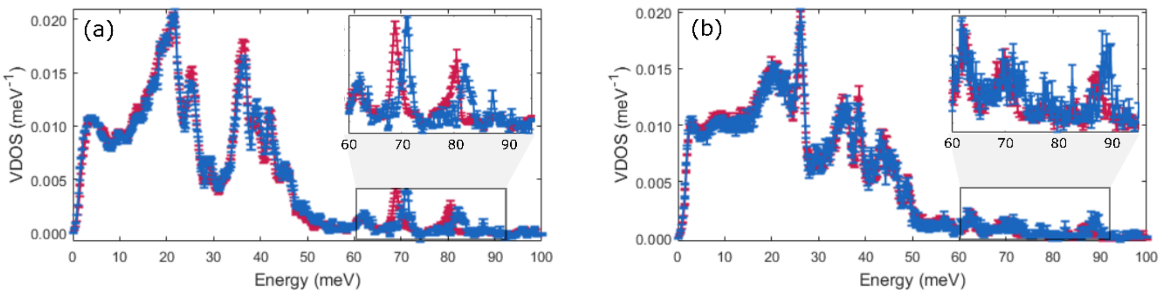

Figure S15. Experimental NRVS spectra of NP as a solid (a) and the and NP in THF solution (b) with error bars generated by the DOS fitting program.

4. Intermediates during the N_2 cleavage reaction

- NMR spectra measured at different time points of the reaction

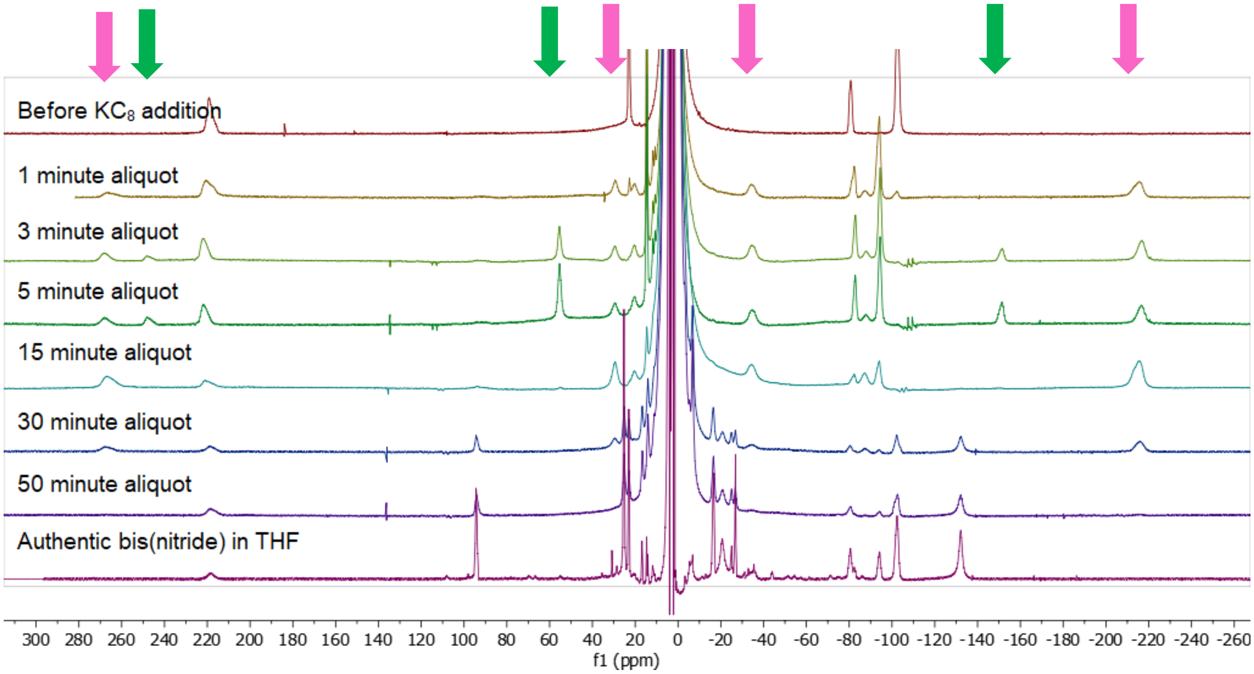

Figure S16. Stacked plot of NMR spectra (all data acquired at –50 °C. The pink and green arrows indicate INT1 and INT2, respectively.

- Mössbauer spectra measured at different time points of the reaction

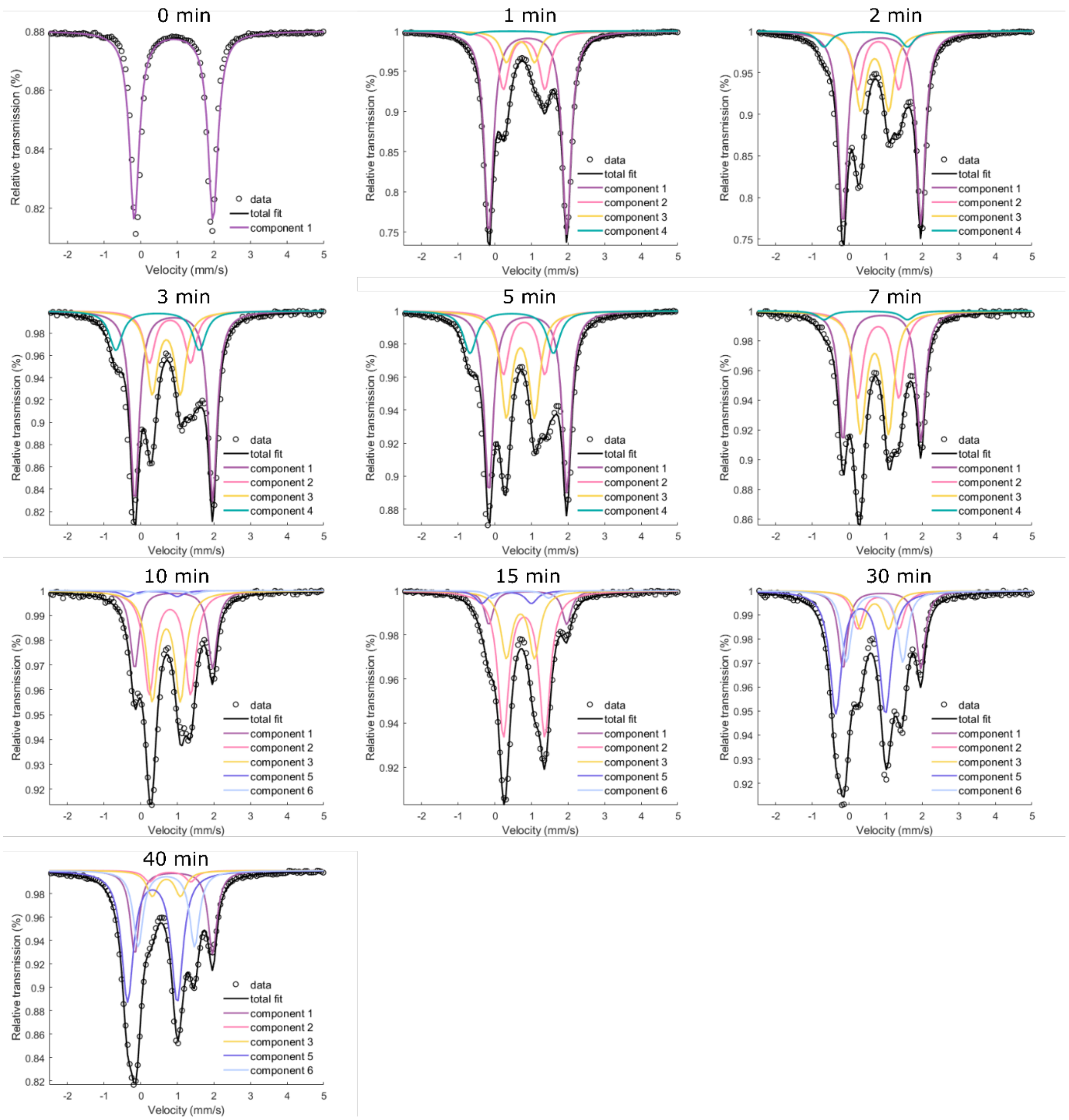

Figure S17. Mössbauer spectra measured at different time points of the reaction.

- NRVS spectra of solution after 3 min of the reaction.

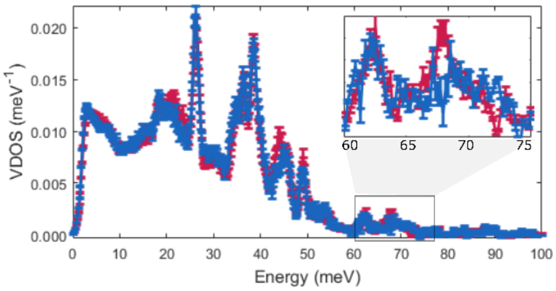

Figure S18. ^{14}N- and ^{15}N-labelled (blue and red respectively) NRVS spectra of the solution after 3 min of the reaction with error bars generated by the DOS fitting program.

- Time evolution of the NRVS spectra in the early minutes of the reaction

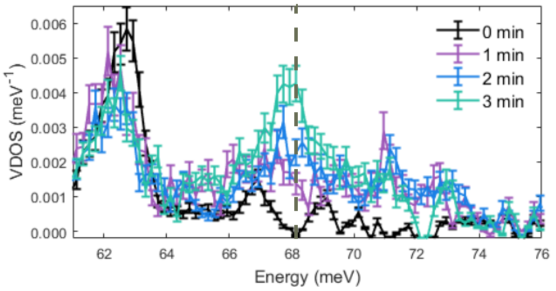

Figure S19. NRVS spectra with error bars measured 0, 1, 2 and 3 minutes after start of the reaction

- Calculated NRVS spectra of monometallic models for INT

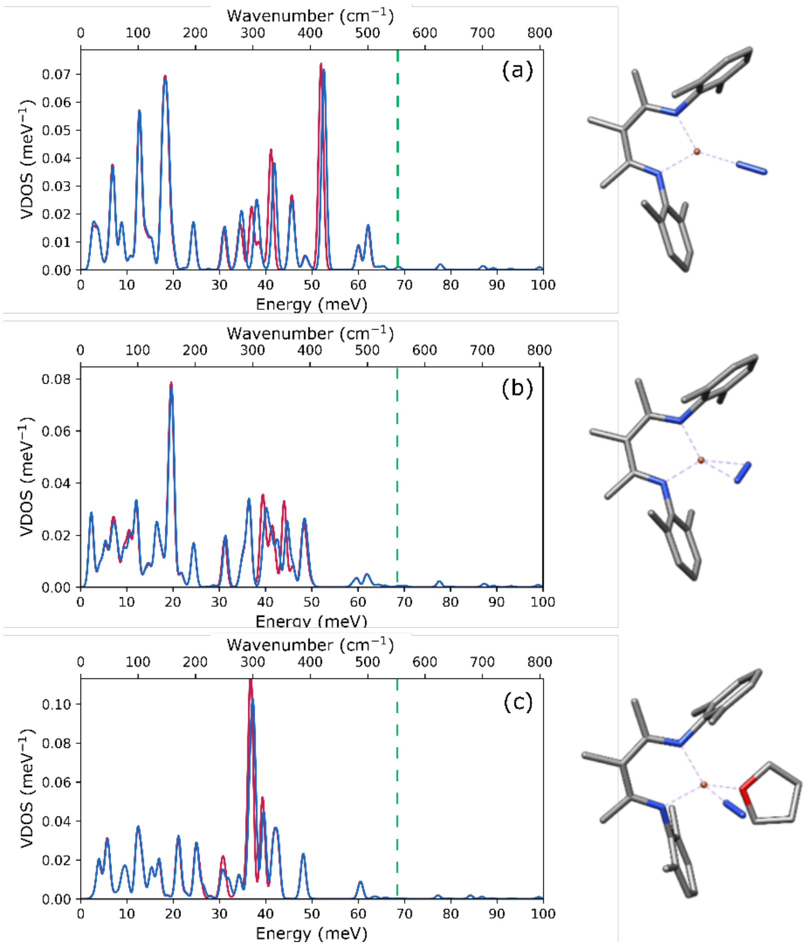

Figure S20. Calculated NRVS spectra of $L^{Me3}FeN_2$(end-on) (a), $L^{Me3}FeN_2$(side-on) (b) and $L^{Me3}FeN_2$(end-on)THF (c). Green dashed line is the energy of the experimental band of INT2.

Table S7. $^{14}N/^{15}N$ N_2-labelling sensitive bands of NRVS spectra of model presented in the Figure S15.

	^{14}N	^{15}N
$L^{Me3}FeN_2$(end-on)	52.6 meV (424 cm^{-1})	52.0 meV (419 cm^{-1})
$L^{Me3}FeN_2$(side-on)	44.6 meV (360 cm^{-1})	44.0 meV (355 cm^{-1})
$L^{Me3}FeN_2$(end-on)THF	39.6 meV (319 cm^{-1})	39.3 meV (317 cm^{-1})
	37.2 meV (300 cm^{-1})	36.7 meV (296 cm^{-1})

- Calculated NRVS spectra of bimetallic models for INT

Table S8. $^{14}N/^{15}N$ N_2-labelling sensitive bands of NRVS spectra of model presented in the Figure 15.

	^{14}N	^{15}N
$L^{Me3}FeNNFeL^{Me3}$	78.4 meV (632 cm^{-1})	76.1 meV (614 cm^{-1})
	51.8 meV (418 cm^{-1})	50.6 meV (408 cm^{-1})
	55.9 meV (451 cm^{-1})	54.4 meV (439 cm^{-1})
$L^{Me3}FeNNFeL^{Me3}THF_2$	83.9 meV (677 cm^{-1})	82.6 meV (666 cm^{-1})
	87.5 meV (706 cm^{-1})	86.8meV (700 cm^{-1})
$K_2L^{Me3}FeNNFeL^{Me3}$	81.6 meV (658 cm^{-1})	79.6 meV (642 cm^{-1})
	54.6 meV (440 cm^{-1})	53.4 meV (431 cm^{-1})

5. Summary of Mössbauer parameters

Table S9. Mössbauer parameters from experiment and calculations.

	Charge	Multiplicity	Experimental		Calculated	
			I.S. (mm/s)	ΔEq (mm/s)	I.S. (mm/s)	ΔEq (mm/s)
$L^{tBu}FeNNFeL^{tBu}$ (solid)	0	7	0.61	1.63	0.67	1.55
			0.73	1.61	0.67	1.55
$L^{Me}FeNNFeL^{Me}$ (solid)	0	7	-	-	0.61	1.85
					0.62	1.82
$K_2L^{tBu}FeNNFeL^{tBu}$ (solid)	0	5	0.50	2.27	0.50	2.19
NP (solid)	0	19	0.29	1.79	0.46	2.22
			0.68	1.54	0.59	1.74
			0.96	1.80	0.83	1.93
NP (in THF)	0	15	0.32	1.36	0.45	2.13
(calculated as triiron model with one THF on each K)			0.70	1.54	0.57	1.72
			0.90	2.13		
(calculated as triiron model with one THF on each K, and a THF on one iron)	0	15	-	-	0.45	1.74
					0.72	1.74
$[L^{Me3}FeCl]_2$ (solid)	0	9	0.93	2.14	0.87	2.44
					0.79	-3.55
$L^{Me3}Fe(Cl)(THF)$	0	5	0.90	2.14	0.88	1.88
$[L^{Me3}FeTHF_2]^+$	1	5	-	-	0.95	-1.76
$[L^{Me3}FeCl_2]^-$	-1	5	-	-	0.87	4.25
$[L^{Me3}FeN_2]^+$ (N_2 side on)	1	5	-	-	0.74	2.41

$[L^{Me3}FeN_2]^+$ (N_2 end on)	1	5	-	-	0.67	-2.21
$[L^{Me3}Fe(THF)N_2]^+$ (N_2 side on)	1	5	-	-	0.89	1.40
$L^{Me3}FeNNFeL^{Me3}$	0	7	-	-	0.54	1.94
$L^{Me3}FeNNFeL^{Me3}(THF)_2$	0	7	-	-	0.80	-1.49
$K_2L^{Me3}FeNNFeL^{Me3}$	0	5	-	-	0.42	-2.19

6. Animations of normal modes of $L^{tBu}FeNNFeL^{tBu}$ and NP

Animations can be found in a separate file.

7. Optimized Geometries

Optimized structure for $L^{tBu}FeNNFeL^{tBu}$

Fe 0.01120652330794 0.07244452721707 2.34325288257724
Fe -0.00720263748430 -0.06105253387091 -2.34494070520633
N 0.00120681350660 0.01752083998915 0.58609319204907
N -1.42075941686921 0.00555217058707 3.61829660605005
N 1.45674759913125 0.20605617976351 3.59604105757323
C -2.42965752756693 -0.00741039376052 5.99079483004946
C -1.26512961786082 0.07497245697338 4.95011547758264
C 0.03004014066714 0.15265318532395 5.51861155716675
H 0.03674428856726 0.18019006909821 6.60760221506096
C 1.31801531411630 0.20265554245922 4.93131939429781
C 2.49530861361737 0.34029789117696 5.95131801211933
C -2.26393875924560 -1.34719067996611 6.75592649475197
H -2.32735001566679 -2.20272989121805 6.06000367055731
H -3.06603327029059 -1.45793093440070 7.50950792471746
H -1.29277556008218 -1.41009593503445 7.27498456124862
C -3.86512275890668 0.02081872413426 5.42525319144415
H -4.10157858777735 0.96428259377691 4.90909277917238
H -4.56773843700462 -0.08057992286518 6.27306256493176
H -4.06851412869606 -0.80177641416724 4.72425781354094
C -2.32451785325017 1.17881628290296 6.98340568606536
H -2.39364523705645 2.14366041996319 6.44931561438662
H -1.38227257422914 1.17898031576703 7.55568677886282

H	-3.15631181671775	1.13300686273343	7.71043273156741
C	3.92411750782324	0.29235102555135	5.36991029652553
H	4.16207873986007	-0.67378315952190	4.89837140792002
H	4.63592810663208	0.44075993205706	6.20299681826697
H	4.11342585062883	1.08043869123529	4.62636769624930
C	2.40835121437550	-0.79613175800944	7.00220470353157
H	2.47717549086772	-1.78589303855225	6.51563888999239
H	1.47247144161483	-0.77299954345777	7.58446129759368
H	3.24809144549202	-0.70982095010733	7.71620745599301
C	2.33263273684088	1.71575589989714	6.65102273222500
H	1.36601480108030	1.80227785488379	7.17508630310590
H	2.38757618806382	2.53526891987397	5.91234032173390
H	3.14124523584382	1.86600235625680	7.39067424058226
C	-2.61754039244525	0.06916163806842	2.85551254046323
C	-3.19375062748649	1.34261703266120	2.56831012274653
C	-4.35273608914448	1.38296562554363	1.77696060821660
H	-4.82494240021329	2.34758918309667	1.56024379965033
C	-4.91616425266507	0.20993519947976	1.24766082710947
H	-5.81985013556575	0.26597842896934	0.62883689891375
C	-4.31369842525121	-1.02839944586363	1.50549492749162
H	-4.75234003910086	-1.94316586142786	1.08600494104231
C	-3.16409025108925	-1.12472612020421	2.31135299440531
C	-2.53397525593904	2.61130173890948	3.10047861421849
H	-2.12256143936384	2.37029572787218	4.09937706104246
C	-3.50699077039571	3.78684980685573	3.27704616014737
H	-2.99903768635867	4.63046045458484	3.77765824798968
H	-4.38199840265415	3.50221290937692	3.88866493379970
H	-3.87902512303974	4.16167294533621	2.30561602217868
C	-1.34151683990897	3.02397277512338	2.21528652365095
H	-0.83225015661963	3.91543189780812	2.62305371066316
H	-1.65470856393646	3.23988842477573	1.17924997560788
H	-0.57899869062689	2.21979787033538	2.14985833131095
C	-2.53295849294536	-2.48055496362489	2.59678561851455
H	-1.65948982192853	-2.29840883927941	3.25012325395618
C	-3.49524054466634	-3.41976042876903	3.34936609370659
H	-3.84651722335381	-2.97035495972225	4.29495351571257
H	-2.99606040082434	-4.37617778727240	3.59094444941451
H	-4.38795263839987	-3.65319747352704	2.73966799245648

C	-2.02472956792537	-3.13214281794217	1.29993478280731
H	-1.33052668921219	-2.47201628221745	0.75289149140928
H	-2.86230430273955	-3.36646823156644	0.61826305138021
H	-1.49636667684292	-4.07888957923109	1.50888255063545
C	2.64313138340688	0.09735945345600	2.82293526926290
C	3.18157307451048	1.25748948653584	2.20317591219407
C	4.32177738585403	1.11483534270495	1.39086695898879
H	4.75431607408100	2.00370746102004	0.91340274613085
C	4.92309098295792	-0.13614463986171	1.20022289626280
H	5.82043329405825	-0.22808737037833	0.57646646839000
C	4.36625500283799	-1.27656861921913	1.80283880527253
H	4.83754903454528	-2.25172744431484	1.63739329509690
C	3.21556089925458	-1.19100182851239	2.60266884059041
C	2.54952355658927	2.62644047366874	2.41385336735239
H	1.68609871529454	2.48288426553304	3.08996268259379
C	2.02142738824678	3.19535121627146	1.08636275017237
H	1.31882515762085	2.50314038624286	0.59200443857381
H	2.84935235446375	3.38493012416194	0.37939737024690
H	1.49703557126104	4.15431792721821	1.24284945992269
C	3.51897032116280	3.61366695670697	3.09222737657495
H	3.88811229642972	3.22445330723379	4.05748089329579
H	3.01896619112017	4.58097611583664	3.28334283124660
H	4.40028558589441	3.81295421511288	2.45454272603336
C	2.56135702494625	-2.42756944419782	3.21171530286256
H	2.16755800251637	-2.13404261954038	4.20369828940060
C	1.35246992665338	-2.88140245548486	2.36942388258709
H	0.57729182884457	-2.08963603313800	2.29993489598626
H	0.86457214094526	-3.76532486948722	2.81776696736714
H	1.64282249065299	-3.12614264157997	1.33317180161922
C	3.53372554138884	-3.59632989620034	3.43161562509732
H	4.42200349087179	-3.28378771455661	4.00957438746681
H	3.88461334890204	-4.02477298790269	2.47450022724859
H	3.03284576696452	-4.40939884537908	3.98687633063561
N	-0.00321011645875	-0.01548071404006	-0.58744411744015
N	-0.11454777600109	1.33723950870672	-3.65157027137661
N	0.08449039723729	-1.53885261206056	-3.56517714906889
C	-0.19649596732364	2.29035261913013	-6.04491741277455
C	-0.08157416335323	1.15059032047390	-4.98059753640938

C	-0.01845678788436	-0.15779632600774	-5.51967109402364
H	-0.02114102038052	-0.19078300493141	-6.60851861556354
C	0.04521373481820	-1.43156508034315	-4.90301913627399
C	0.15044686329408	-2.63302732214102	-5.89868362246764
C	-0.15578532630036	3.73850346748121	-5.51318829261962
H	-0.95673091489893	3.95585717772374	-4.79143516520054
H	-0.28592474773623	4.42149150408229	-6.37301731828288
H	0.80243830003559	3.98935809183487	-5.03216907482098
C	-1.55762581078298	2.10407688051515	-6.76626236160351
H	-2.39203642366079	2.18094474426920	-6.04650556285805
H	-1.63282331212222	1.12114286976912	-7.26097167147985
H	-1.69366273049043	2.88849513612087	-7.53420650893343
C	0.96067162933839	2.16466364953138	-7.06883684913210
H	0.89020735561589	2.97786630048120	-7.81466454253207
H	0.94824729975990	1.20840829616189	-7.61729162686436
H	1.94052517899165	2.25057126280071	-6.56525145225937
C	-1.01522773842650	-2.56584376306253	-6.91866942398735
H	-0.95408445999708	-3.42406263609225	-7.61304145667303
H	-1.00321565121206	-1.64527083802322	-7.52510100251139
H	-1.99090362102948	-2.61573147439972	-6.40240432961521
C	1.50569774930864	-2.49241256009931	-6.64136263878152
H	2.34645868494173	-2.53551479919832	-5.92611135504057
H	1.58106466563281	-1.53788209760167	-7.18879251049972
H	1.63037716760250	-3.31771117210302	-7.36718648201562
C	0.11421904958837	-4.04757263439956	-5.28295949291580
H	0.92338827476339	-4.22264129235040	-4.55895965839646
H	0.23520102773256	-4.77921730365232	-6.10320415574505
H	-0.83846600355070	-4.26976434995046	-4.77764137762298
C	-0.02885113414501	2.54973348606439	-2.91698202286984
C	1.25190851817439	3.13440467890186	-2.68402136749202
C	1.31414666639338	4.31205399878744	-1.92218689630150
H	2.28357036431403	4.79197689502718	-1.74826886874893
C	0.15719290976895	4.88500984329652	-1.36816205343932
H	0.23063945655921	5.80365640751790	-0.77365279288185
C	-1.08677831451178	4.27270341679771	-1.56934316059701
H	-1.98852971516292	4.71846469532423	-1.12961291925808
C	-1.20577882002798	3.10429119476637	-2.34458742500067
C	2.50486014087889	2.46482688645228	-3.24114279591823

H	2.23714032321960	2.04218205274260	-4.22858102152964
C	2.93961820543985	1.28144564594086	-2.35387889300924
H	2.14712067989419	0.50722434136502	-2.28086453663844
H	3.16109279237712	1.60150994410729	-1.32119429209541
H	3.83348458449903	0.78156007073757	-2.76804971188746
C	3.67737532802653	3.43299056059218	-3.45952577561407
H	3.37847794837223	4.30254528441105	-4.07211325989861
H	4.50577033863527	2.91769186221082	-3.97767856638002
H	4.07946997546216	3.81394108169623	-2.50250638792422
C	-2.56748002290340	2.46007385013037	-2.56621932222324
H	-2.40289516911146	1.56640446503421	-3.19683048921004
C	-3.53357610366614	3.39538999425863	-3.31874498958115
H	-3.11551469316736	3.71732582512546	-4.28880588040683
H	-3.75038173060306	4.30646007902534	-2.73044244356349
H	-4.49547222706055	2.88659004777492	-3.51406751778676
C	-3.17533017031411	1.99157701633604	-1.23376993207726
H	-2.49733664625775	1.31310415042701	-0.68882640895542
H	-4.12866324228160	1.45851150747823	-1.39511412833375
H	-3.38613932984892	2.84932987931840	-0.56975384127067
C	0.00063825469088	-2.70815911459877	-2.76269431346312
C	1.18015569716716	-3.23570005250236	-2.17076178815497
C	1.06306878713726	-4.35599341420782	-1.32730927846886
H	1.96689664939945	-4.77974418373354	-0.87053086341097
C	-0.18192104557690	-4.94885644760490	-1.07927240730981
H	-0.25417866127948	-5.83048901889855	-0.43101446927320
C	-1.34091617889547	-4.40396042477827	-1.65668313303466
H	-2.31103293371448	-4.86824866284820	-1.44735369085847
C	-1.28013130112811	-3.27322142407837	-2.48672648847757
C	2.54237254008894	-2.61307268210691	-2.44319133587195
H	2.37811612853021	-1.77083584296177	-3.14071304696976
C	3.15227722801615	-2.04501569682807	-1.15065630930799
H	2.47193871927362	-1.33418215967125	-0.65167965912792
H	4.10118669451767	-1.51836954430647	-1.35404496142547
H	3.37275032469053	-2.85186613195928	-0.42847236184412
C	3.50800380583835	-3.60387926132098	-3.12160501638070
H	3.08951574080278	-4.00181963540224	-4.06293556689591
H	3.72657569147725	-4.46567935231988	-2.46388918082111
H	4.46917803714256	-3.11097674809677	-3.35701473911947

C	-2.53524891207189	-2.63170738264993	-3.07042771556286
H	-2.26891038846377	-2.24616035501870	-4.07314972105281
C	-3.70416138020755	-3.61189397735918	-3.25121275418836
H	-3.40212692752216	-4.50319175850040	-3.83011183929751
H	-4.53459150663056	-3.11994903627894	-3.78853607837557
H	-4.10482390113626	-3.95707936469514	-2.28014801053766
C	-2.97206656306590	-1.41719132856349	-2.22767587075624
H	-3.85458563689007	-0.92152409007378	-2.67011143397186
H	-2.17067352616639	-0.65203298330906	-2.16232246216896
H	-3.21222894099333	-1.70390971238981	-1.18926687059028

Optimized structure for $K_2L^{tBu}FeNNFeL^{tBu}$

Fe	0.01586549009120	-0.00997525866423	2.34744356589428
Fe	0.12600995679911	0.01470672580688	-2.36409384868205
K	0.94106152486023	2.48957947435489	0.03030480964326
K	-0.75478454441065	-2.50180959950540	-0.02985859688121
N	0.06929166703700	-0.00619693263413	0.60617273593678
N	0.09360835044395	0.00182237034401	-0.62239007953950
N	-0.07525940886339	1.41592729310150	3.64846140938217
N	0.01987871995061	-1.43654991632925	3.64983520598855
N	1.00870349502947	1.16199236444714	-3.64504065737839
N	-0.70677710884190	-1.12036239145548	-3.68837475263951
C	-0.31521523823662	2.42920789263175	6.03814436820571
C	-0.13584053966818	1.27246689597802	4.99226363470005
C	-0.09529784380080	-0.01228774663694	5.57197722788032
H	-0.12837211001240	-0.01267703260808	6.66059529850771
C	-0.01021243139270	-1.29591050890856	4.99466514309826
C	0.12868630303692	-2.45261219002908	6.04664390229782
C	-1.68022523186145	2.20430064243843	6.74337653129377
H	-2.50580811059540	2.25773022688220	6.01073388519004
H	-1.85017735830177	2.98583882624113	7.50795097324502
H	-1.73843050457432	1.22124665979721	7.23916613247304
C	-0.33342127683552	3.88157915413062	5.51541134106237
H	-1.13287248135706	4.06311089099663	4.78250599824089
H	0.61824409967042	4.18809369173719	5.05588556271933
H	-0.51727890309954	4.54867682715927	6.37814520321734
C	0.83120186457447	2.35981623603351	7.08092912056292
H	1.81237587515808	2.49218431764008	6.59068044024668

H	0.85676619106383	1.40246926983223	7.62700075282243
H	0.71234842903735	3.16781809413371	7.82672383610398
C	0.15562351112739	-3.90557236859731	5.52559534435992
H	0.97363997187159	-4.09443877062662	4.81566691423766
H	-0.78458971224802	-4.20538255990598	5.03846518277696
H	0.30856224216569	-4.57324561470344	6.39390145227935
C	-1.04838351642821	-2.38159945724412	7.05470459208428
H	-1.08475682404661	-1.42924893238181	7.60861893231140
H	-0.95662789265002	-3.19552268251578	7.79782077396304
H	-2.01568260503686	-2.50430526187903	6.53514231163199
C	1.47154538559122	-2.22746169925427	6.79329580973411
H	1.50961671897035	-1.24509384296024	7.29272858999984
H	2.31905338677806	-2.27406370044327	6.08579364006992
H	1.62161591310186	-3.01069059252366	7.56036216811116
C	0.11468889010016	2.65257450544078	2.99420058428228
C	1.42687255263510	3.22457644229221	2.92582908618079
C	1.59590551431152	4.45130791833747	2.25643852762082
H	2.59444636592919	4.90154314058427	2.20337870703747
C	0.51048345271441	5.11406354487676	1.65310630072902
H	0.66278665946836	6.07044028657255	1.13974854692163
C	-0.77166156576617	4.53762514264976	1.71968285323310
H	-1.62088926247254	5.05871474480806	1.25919837434446
C	-0.99550653669379	3.32263380655409	2.39385924545568
C	2.61669961346555	2.47810371355819	3.52021193740399
H	2.22411222884981	1.83565043843423	4.32877167646222
C	3.68142776834081	3.40258630111864	4.12819456570333
H	3.24102703034432	4.10395572796991	4.85985680701188
H	4.20011461230961	4.00139043321585	3.35641660609818
H	4.45306372225792	2.80618945206089	4.64735975250239
C	3.22528269901579	1.53538942896100	2.46116766877091
H	2.45333665515828	0.83228759424605	2.08268609051146
H	4.04573637946467	0.92963847488942	2.88594986489444
H	3.63824985090808	2.11059994697617	1.60926578077261
C	-2.39394576527865	2.72835285928179	2.49037554676816
H	-2.33079683041118	1.90106094773527	3.22121889257045
C	-3.43985322313550	3.73937731938265	2.99182291933892
H	-3.14762277152522	4.18392478161203	3.95983775650354
H	-4.41795041465240	3.24280652886723	3.12686011524569

H	-3.58864456835311	4.56673011288566	2.27322415498503
C	-2.80801601216447	2.11116200703159	1.14408834925330
H	-2.07606472174509	1.34256140235716	0.84007270357877
H	-2.86364937341338	2.87780051970541	0.34941585881282
H	-3.79725233346417	1.62492080617386	1.21710290826172
C	-0.10901588980912	-2.67276364068287	2.98109032763674
C	1.04467984404592	-3.31422690531691	2.43532474337376
C	0.88436283499609	-4.53167023867603	1.74753042851605
H	1.76914571444976	-5.02883121932168	1.32893631356831
C	-0.37895763481667	-5.13605890563202	1.61070065142108
H	-0.48269806387411	-6.09343445130257	1.08712824734031
C	-1.50954577469823	-4.49797185131564	2.15499776412234
H	-2.49430165685614	-4.96793486105002	2.04544621184225
C	-1.40304265192178	-3.27213214872437	2.83806510731275
C	2.42295120939524	-2.68859306461487	2.59549552169836
H	2.28987560706534	-1.80329636778415	3.24431607874604
C	2.94373200242375	-2.18536790241611	1.23955930694075
H	2.23164856755419	-1.46419484914880	0.80165768114643
H	3.08418695859333	-3.01909032137881	0.52682370599828
H	3.91478935730257	-1.67109907920072	1.35338535485866
C	3.42971500247907	-3.63785521500555	3.27003664504519
H	3.06795046478929	-3.98448637443820	4.25419175799342
H	4.39667593727144	-3.12520574546144	3.42506088267388
H	3.62568037300688	-4.53305111184446	2.65086730202518
C	-2.63716383497781	-2.55225118656551	3.37155011028112
H	-2.30199145985128	-1.91622245926478	4.21050405572363
C	-3.19499032858270	-1.60283800098106	2.29141364683260
H	-2.41003029285021	-0.88933877898387	1.96332742728554
H	-4.04311499171181	-1.00842471458696	2.67600117020389
H	-3.55388366567394	-2.17209765251535	1.41167664267165
C	-3.72218071308736	-3.50002537015500	3.90308067691131
H	-3.31608620343980	-4.20080362986302	4.65492731447898
H	-4.18063379313598	-4.09952425182063	3.09460127895005
H	-4.53494874170006	-2.92166789211394	4.37792641874048
C	1.76734299833519	1.91238001141065	-6.02427720291443
C	0.96418002837773	1.04405933585173	-4.99184795335367
C	0.18530610026136	0.03194876405307	-5.59001400653328
H	0.20359497442059	0.03889813685323	-6.67914253324920

C	-0.61193306921091	-0.98822021207151	-5.03140452065837
C	-1.37862525500872	-1.84416690240973	-6.10113599805165
C	2.62544482857525	3.07586659232686	-5.48242319644227
H	2.02753275416424	3.86372957081140	-5.00045556094358
H	3.15190497843672	3.53489715887735	-6.33978689215639
H	3.38947273186617	2.74752411409961	-4.76311633537735
C	0.77826135053726	2.54385003449687	-7.03908886521583
H	0.05750839914127	3.20261144865993	-6.52212157827103
H	0.19993944995972	1.79113347087003	-7.59978687865173
H	1.33207971525767	3.15670825546305	-7.77456992555132
C	2.74443335724891	0.96060649190701	-6.76614652364411
H	3.32463604982408	1.52141705748334	-7.52318732173756
H	2.21930022953405	0.13709335527638	-7.27778003859783
H	3.45837001957647	0.50903315196608	-6.05361242356743
C	-2.34889875075673	-0.88664140130651	-6.84433590207179
H	-1.82030294581872	-0.04672671370937	-7.32505343136147
H	-3.08344707314156	-0.45825784673380	-6.13844285769139
H	-2.90603270302900	-1.43599024144050	-7.62674020414141
C	-0.35914724421780	-2.44231381698567	-7.10619844458203
H	0.21962799898501	-1.67195782973840	-7.64179400841547
H	-0.88873876174430	-3.04919691346494	-7.86407165775216
H	0.36009701951048	-3.10037780320402	-6.58616849045347
C	-2.23335818109613	-3.03067046148149	-5.60571017818071
H	-1.63620614295109	-3.81939598451633	-5.12392828124102
H	-2.72911697609104	-3.48011438874222	-6.48617885407343
H	-3.02183525456296	-2.72990360044891	-4.90094087211524
C	1.60185313180012	2.25036297503521	-2.96839951840521
C	2.90401179509916	2.11398610734619	-2.39745419661356
C	3.46216491374872	3.20578398508321	-1.70626881501060
H	4.46325054116477	3.10490079314865	-1.26747996830207
C	2.77508286632967	4.42794689092987	-1.58836747486922
H	3.23111608605358	5.27303678749645	-1.05992770411799
C	1.49280528177481	4.55304579562486	-2.15487539655738
H	0.95445886910442	5.50345576671000	-2.05770470902756
C	0.88714231466289	3.48563329530574	-2.84410016625087
C	3.66723025922745	0.80308836320879	-2.52386058786982
H	3.08711125612359	0.16904607479287	-3.21970543954980
C	3.69507918640653	0.07952285395655	-1.16722246628261

H	2.66343074478690	-0.09169026166710	-0.81311782678523
H	4.23637517998882	0.66991495557253	-0.40437934010865
H	4.18944671425406	-0.90494568293863	-1.24857749168516
C	5.08224349140255	0.98725673950949	-3.09907750081206
H	5.57176662428913	0.00560183392483	-3.23471711478878
H	5.72442256439292	1.58487738426681	-2.42560443912994
H	5.06124281208285	1.49507599566238	-4.07976206804148
C	-0.52692746087321	3.60833286558897	-3.40087570636080
H	-0.61912881219090	2.86715148545932	-4.21492769133331
C	-0.83964021347323	4.99411815475456	-3.98478314910923
H	-1.82871691262244	4.98721319271777	-4.47705969615162
H	-0.08651664869209	5.29832685200343	-4.73399749203517
H	-0.87213307586460	5.77521722409093	-3.20252394583995
C	-1.54733843903864	3.20812792372756	-2.31599212304989
H	-1.34499212960901	2.17617905520891	-1.96006210385485
H	-2.58065354857829	3.22813425783186	-2.70668903206089
H	-1.49855161579348	3.90266279854631	-1.45468947837516
C	-1.31142813894632	-2.22307208474351	-3.04604812290320
C	-0.58797314687568	-3.45279339434124	-2.91411581787751
C	-1.20508534317974	-4.53548257700498	-2.25984096905759
H	-0.66115223842131	-5.48220339110851	-2.15807655214056
C	-2.50658588827493	-4.43041756489179	-1.73431666440866
H	-2.97137266369673	-5.28710347079300	-1.23292018103148
C	-3.20257983508429	-3.21408145136123	-1.85995136127456
H	-4.21939113934868	-3.13012031318752	-1.45513190561429
C	-2.63425229716450	-2.10787108289503	-2.51917894549477
C	0.84502916856832	-3.55200612312256	-3.42584369573600
H	0.95045983445946	-2.80877996099256	-4.23655677183816
C	1.82390591487122	-3.13456057649711	-2.30952034044949
H	1.58338248324171	-2.11128132923193	-1.95096130621128
H	2.86773300475670	-3.12314783487122	-2.67156301583868
H	1.77106654730794	-3.83744988871715	-1.45542592631868
C	1.20097210114737	-4.93106935332071	-4.00024824429195
H	0.47909513413023	-5.24771986974618	-4.77469023023215
H	1.22194259344732	-5.71268831599897	-3.21808631820398
H	2.20560819614550	-4.90558506000418	-4.45928181609722
C	-3.41034167775492	-0.80667167339551	-2.66400449868886
H	-2.82065757230136	-0.16487276844404	-3.34438982399365

C	-4.80685031753295	-1.00903270899826	-3.27759497229561
H	-4.75346162232488	-1.52184911646567	-4.25446312885328
H	-5.30243033461317	-0.03326713721996	-3.43219762761762
H	-5.46157154600853	-1.60934501699334	-2.61880301461328
C	-3.48649148291765	-0.08263532341627	-1.30924853555230
H	-2.46870824120191	0.10752221598834	-0.92598652523007
H	-4.03851754029361	-0.68168324828364	-0.56096310701842
H	-3.99744460115609	0.89191619071297	-1.40603481248697

Optimized structure for NP as a solid

Fe	1.91271402165146	0.44561709116253	0.10265246887072
Fe	0.79096278411249	0.33149067702673	-2.39156842496006
Fe	3.48411594163091	-1.96326170129553	-2.01662338995594
Fe	-2.96271465039539	4.92230059073030	0.58760494225564
N	0.48653762392071	1.30497105703513	-0.79864535346071
N	2.28984322928865	-0.61444964664892	-1.51733741856970
N	3.46114653447343	1.52748508564372	0.94650285107208
N	1.50553359414735	-0.15131864815425	2.05211857384235
C	5.44106977321430	1.92081151334794	2.37568758249337
H	5.64436117101510	2.74251446287859	1.67526335740581
H	6.29140034625022	1.21476967891419	2.32751718896551
H	5.43336558635859	2.33213813909082	3.40042189285817
C	4.14094107693904	1.19750418601830	2.05882732486728
C	3.71540659361111	0.22019254545466	3.00373041987606
C	2.39674297920783	-0.31674817871673	3.03971632410661
C	1.97482176728957	-1.07531507395099	4.28857238655230
H	0.92881769986129	-1.40664073112358	4.22660551837562
H	2.09597603739206	-0.46150843910790	5.19870138670605
H	2.59767129099486	-1.97637881976898	4.41194828656306
C	4.65983185605079	-0.08374622802936	4.15489620093603
H	4.36424559897402	-0.98513935514528	4.71077822941516
H	4.71118001085176	0.73841086004602	4.89885946354943
H	5.69617035207812	-0.25502747454652	3.81038153608625
C	3.84862808752585	2.71801118310349	0.27326008966496
C	3.31349348861247	3.95658714317073	0.72195627811376
C	3.76253741894643	5.14855703085587	0.11792435398865
H	3.35774925402369	6.10591331665050	0.46859661764955
C	4.73429141147065	5.11824798106309	-0.89419918593854

H	5.08757729374356	6.05356986984172	-1.34539903743365
C	5.25457495550029	3.88739720736287	-1.32309804028429
H	6.02684750691718	3.86161762456186	-2.10346249774629
C	4.81887799810763	2.67260411656508	-0.75934997727704
C	2.34661441886753	3.97948441486805	1.88106885003687
H	1.86132980169697	4.96405801299915	1.99211710974124
H	2.86109228087327	3.75040672648606	2.83347666558804
H	1.56204526619623	3.20864763651175	1.77648136417119
C	5.42607016672971	1.35576618237679	-1.15919640013609
H	4.64056150462317	0.61202976889026	-1.40454495175435
H	6.02407498039152	0.92571092195128	-0.33458793957880
H	6.08600608061246	1.47325524146549	-2.03248307640314
C	0.13985805441294	-0.42186820659892	2.32604279123604
C	-0.44718881439729	-1.58038239529992	1.74859121800932
C	-1.80295374566391	-1.85118851350944	2.01353295598855
H	-2.25584757582115	-2.75109646750096	1.58551949887425
C	-2.56856999282910	-0.99309411516580	2.82306984699440
H	-3.62593614216473	-1.21613900409385	3.00918569605667
C	-1.96983056747934	0.13770075380508	3.40437310079101
H	-2.55758198669543	0.80724648617870	4.04552220607730
C	-0.60702369928545	0.43449643846615	3.18485558623904
C	0.42535033715842	-2.51354349476509	0.96139803272810
H	-0.11912821431471	-3.42234082130226	0.66620770604712
H	1.30481723794745	-2.81083776703153	1.55549042737386
H	0.82880757971358	-2.02917829261759	0.05001136365445
C	0.04877302138172	1.58825724134057	3.89935885236015
H	-0.67483189970500	2.39523451677360	4.09450763693109
H	0.87905349206662	2.00447241172842	3.30934354667339
H	0.46950715329514	1.26094186558785	4.86996615024036
N	-0.69112600234033	-0.81318980985635	-3.27329772962485
N	0.70046660696116	1.46683238421239	-4.13489042548787
C	-1.80770494073464	-2.10306730102507	-5.06332467479219
H	-2.38543981712037	-2.53912426095421	-4.23643310050797
H	-1.23340402248474	-2.91967491359203	-5.54093238960869
H	-2.51512339334288	-1.72823654835953	-5.82305788389668
C	-0.86410663174572	-1.00659911230815	-4.59276650754550
C	-0.23518351461556	-0.23133780449689	-5.60854406395638
C	0.40410146175716	1.01665613077680	-5.36253654444957

C	0.71821332533204	1.89455594120106	-6.56417929684231
H	1.13476151855950	2.86497134662663	-6.26055548432686
H	-0.17829546048959	2.07355325609222	-7.18341234710260
H	1.46753696294494	1.40321525100479	-7.20693545144699
C	-0.46030125428047	-0.65566319959607	-7.05038948358472
H	0.28916032538242	-0.22573338445141	-7.73189567912238
H	-1.45223884245954	-0.34663241612129	-7.44261251309302
H	-0.40195608125124	-1.75082077455030	-7.17647968554077
C	-1.59488377589173	-1.46366947882991	-2.38923333527898
C	-2.83714179954064	-0.83392945849875	-2.10292668205224
C	-3.77192106490661	-1.51056013024117	-1.29297495845290
H	-4.73425120928344	-1.02976375562384	-1.07661797247878
C	-3.49303139095974	-2.79224874372642	-0.79400941623153
H	-4.23493868012353	-3.31679729108656	-0.17947185883894
C	-2.26340196810679	-3.40117660203435	-1.08830419422292
H	-2.04837074062765	-4.41020915466933	-0.71200751790009
C	-1.29445634011708	-2.75098808134844	-1.87680636184263
C	-3.16237606218092	0.49884124533077	-2.73242103893701
H	-2.30585675313932	1.19432317440131	-2.68094039832726
H	-4.03651298806039	0.97532513184380	-2.25667552620442
H	-3.38799273948550	0.38731301735072	-3.80991919264429
C	-0.00519294412260	-3.42725558292863	-2.25307769777120
H	0.11946282097456	-4.37254108298634	-1.70362129489256
H	0.86496297144955	-2.77317054416468	-2.03650406387569
H	0.02844276778114	-3.65275906628579	-3.33482877084242
C	1.01417321849581	2.84228957187300	-3.98399673984950
C	2.36206316500713	3.19981471135850	-3.71262915949365
C	2.68077492086196	4.56323859525882	-3.56786942341073
H	3.72075694641349	4.84593866706487	-3.37625699687315
C	1.68921958386499	5.55447721738317	-3.67799852858264
H	1.95700323471700	6.61068841078057	-3.55460175027743
C	0.36293728501835	5.18771778347688	-3.96443209127522
H	-0.41506776584287	5.95570557822951	-4.06250651613557
C	0.00375171614887	3.83370504740406	-4.14035180442039
C	3.39907327789222	2.11653465774272	-3.68212969313522
H	4.41393964925501	2.53016236888286	-3.59226908522558
H	3.34957419390681	1.50833010464125	-4.59949267684823
H	3.22827306771305	1.41025635535392	-2.84593836638288

C	-1.39620853844288	3.45814666429737	-4.55281417702478
H	-2.12860668064380	4.19253505430341	-4.18285868296946
H	-1.67384264702484	2.46743922728288	-4.16225605996817
H	-1.48277255932842	3.41130146233376	-5.65570810502836
N	4.50303175044613	-2.38203921321052	-3.65135921211706
N	4.25561634946817	-3.54553286048213	-1.12053952323216
C	5.77526933707408	-3.67466936399101	-5.34030567042951
H	5.53349294218163	-2.82688021084088	-5.99636905054221
H	5.34373239732517	-4.58506433521980	-5.79706891713513
H	6.87193463432394	-3.81025174374084	-5.34255259127343
C	5.24252378167676	-3.47909386830025	-3.92918020573805
C	5.57529805053250	-4.46389597451609	-2.96012723590476
C	5.17652133499367	-4.41444022487757	-1.59728050770054
C	5.84060561276553	-5.37570734265782	-0.62060646680543
H	5.75594023470103	-5.01235539123626	0.41468383877450
H	6.91035712218048	-5.49847316614112	-0.85638362359012
H	5.38292163901512	-6.38361705648868	-0.64658284418045
C	6.44924152396068	-5.62566929813047	-3.40940491825059
H	6.35843413941360	-6.49480206988473	-2.74023011983767
H	7.52836313009166	-5.36827095199437	-3.46247384599629
H	6.16352989889118	-5.98231951088435	-4.41358711116639
C	4.37468584122170	-1.39352389245215	-4.66434834324207
C	5.42620102200557	-0.46199806340419	-4.86709273974396
C	5.32701623584334	0.43648030894527	-5.94572272527911
H	6.13377822685849	1.16224778395553	-6.11296477664484
C	4.22553150901014	0.39893838987338	-6.81506512553976
H	4.17205842228857	1.09049368537459	-7.66478686517173
C	3.19517578973653	-0.53046284713501	-6.59633129253632
H	2.35043411544956	-0.59013419985442	-7.29164649544604
C	3.23967611008310	-1.42491722003162	-5.51324575284059
C	2.16450813808102	-2.45261163981202	-5.27746890723789
H	2.59333121790655	-3.45814077336602	-5.12126246300772
H	1.45914089557464	-2.49329163365919	-6.12177479687754
H	1.58979530718967	-2.20259941414550	-4.36662331591873
C	6.65569619783842	-0.51562188688005	-3.99621304739950
H	7.22670534426865	0.42703944506459	-4.05004010572574
H	7.33226716295476	-1.33309927338289	-4.31279481455918
H	6.39237750740927	-0.72047320123033	-2.94467054145760

C	3.81581692659428	-3.74523906625518	0.21615290621650
C	2.91791112601901	-4.80653172553303	0.50412123787358
C	2.58948877224999	-5.06180525364844	1.84875689123366
H	1.89264078870636	-5.87753264091676	2.08267762814507
C	3.15099916035585	-4.30099277871305	2.88470594644614
H	2.90605193276904	-4.52876236256251	3.92939109081632
C	4.02779856745590	-3.24696767063823	2.57880691887691
H	4.49204091630031	-2.66592359860742	3.38280736792315
C	4.35680515930004	-2.93664980506246	1.24854403796897
C	2.37424563629094	-5.67196676565833	-0.60534801608878
H	1.39498724606511	-6.10053625961150	-0.32963706685658
H	3.05137706366383	-6.51941762177046	-0.82788597183319
H	2.26735442845074	-5.09876551277826	-1.54062387844812
C	5.31818590019527	-1.83048756443898	0.90226317399292
H	5.76447748033509	-1.39233360468071	1.80854465811823
H	4.79504978861827	-1.02233758688405	0.35696477091183
H	6.12876240366475	-2.19030204952809	0.24320211963084
K	0.46909884971422	4.02192704855819	-0.70692306839351
K	-2.06153908541107	1.27256962888276	0.14207135188507
Cl	-1.36724250100800	3.78695850765523	1.83690784127215
Cl	-2.54433883514459	3.93841320400067	-1.47348518882526
N	-2.91432445454009	6.84496391786688	0.71570195264733
N	-4.79978818065233	4.86172379696007	1.16495123616386
C	-3.56198849379610	9.17655534062443	1.08376976312207
H	-2.51533890551395	9.34240809281549	0.78345054173455
H	-3.70269146739534	9.62209431307171	2.08517568354364
H	-4.20699085328032	9.73809292719825	0.38350550469200
C	-3.89100875769778	7.69385006585143	1.07968898921545
C	-5.19393152798594	7.25021568800671	1.45274749720893
C	-5.60597493081643	5.88398943986786	1.49247354540055
C	-7.02154062170798	5.56308870149238	1.92913004550465
H	-7.19713204284884	4.47587662542041	1.93583328641081
H	-7.76598028275019	6.02804995280357	1.25641932618549
H	-7.22780792301236	5.95331291271857	2.94270495719273
C	-6.23805934920673	8.28136615936985	1.84828816003337
H	-5.87131211547040	9.31181066610673	1.74112932165701
H	-6.56387838613730	8.16876380484307	2.90169620286139
H	-7.15319662955084	8.20676727308606	1.22941260127235

C	-1.60436460326485	7.23925258236150	0.33695121331581
C	-1.31847320432707	7.47269222752507	-1.03892161127769
C	0.01718587382525	7.71518540560016	-1.41214146665392
H	0.24484908595827	7.90138883923620	-2.46841439311574
C	1.04835767524124	7.73028591619860	-0.45867350440543
H	2.08302909999441	7.91204335650643	-0.77338032696904
C	0.74340309784180	7.53835047805897	0.89996937765553
H	1.53966239784498	7.58941362886611	1.65456357444559
C	-0.57940754253102	7.30497956882941	1.32460684422496
C	-2.42558180971172	7.45151441508502	-2.06041846945457
H	-2.05929362026691	7.78706881803269	-3.04515432624868
H	-3.26786445199602	8.10058088160591	-1.76200864064326
H	-2.83002345347601	6.42767527029278	-2.16703175692080
C	-0.91726133748425	7.13182282817458	2.78243090662591
H	-0.02773483940172	7.29101994130302	3.41475412956864
H	-1.30098339162059	6.11290443401121	2.97183374519093
H	-1.70266759178795	7.84045835123579	3.10157498014309
C	-5.18331156301295	3.49538060271397	1.19544769417281
C	-4.94434378881879	2.73252961912606	2.37411439531829
C	-5.18993116951655	1.34692916090863	2.33452176356967
H	-5.01241784310221	0.75318818670849	3.23921825792900
C	-5.66057348575704	0.72400297402556	1.16713891930774
H	-5.83476715958717	-0.35864834527177	1.15517490444610
C	-5.93024650174208	1.49793382668761	0.02535768265982
H	-6.33684264453594	1.02275699561815	-0.87739147421228
C	-5.71237526799790	2.88985435128261	0.01949069814516
C	-4.43355802377032	3.40224208592319	3.62309016188934
H	-4.41160882861973	2.69431280726279	4.46861155238197
H	-5.06131225627514	4.26511748370359	3.90866906023902
H	-3.40949772510558	3.78703421522076	3.46196395165897
C	-6.02709243247143	3.72758179130519	-1.19235244541437
H	-6.70041568833601	4.56636772861668	-0.94009404280171
H	-6.50792839915191	3.11963913046026	-1.97700437063045
H	-5.10195711574144	4.16571579325546	-1.60903502400222

Optimized structure of NP solvated in THF

Fe	2.06326080540273	0.56141203538973	0.05880187099426
Fe	0.88711965618581	0.44047589728484	-2.36563624101861

Fe	3.44467654044949	-2.00298332833974	-1.99557438460423
N	0.65738662385549	1.48328266828225	-0.80825310013752
N	2.37245900267118	-0.55284146922656	-1.53000688137114
N	3.63201484685765	1.54894413720187	0.95919988681927
N	1.48248788111618	-0.02979849424183	1.91830153968311
C	5.57590197615085	1.72535144071371	2.46629079487548
H	5.92137705139936	2.45936639128355	1.72443430467999
H	6.32968046529505	0.91859545469104	2.52413628928533
H	5.55913040880851	2.21221507234486	3.45807498648993
C	4.21874122891086	1.14684556775988	2.10272982648479
C	3.64240111441143	0.22767432666801	3.02417913590247
C	2.28138910182764	-0.20927614661654	2.97903766861355
C	1.71606887179491	-0.87719677172263	4.22191420847418
H	0.62492526517216	-0.99093245807871	4.15820012305285
H	1.95664558417416	-0.30548912112546	5.13427697075546
H	2.15167595649627	-1.88684474208249	4.33969775169990
C	4.48011912069915	-0.13789264401164	4.23989871060178
H	4.06752939788437	-0.99685555590720	4.78805748929537
H	4.56562088895828	0.69575746441552	4.96747160533434
H	5.51341925223569	-0.41210211743432	3.95924855493452
C	4.11454444058161	2.73296490443704	0.34090074453741
C	3.72607347858149	3.99443522409371	0.87771011222333
C	4.19651617359380	5.17501080111393	0.26473429840612
H	3.89804878287363	6.14828326688605	0.67860196304304
C	5.05606908163296	5.11224267577306	-0.84455118266265
H	5.42061751068074	6.03512500223846	-1.31222210774725
C	5.45500469973650	3.86214762263201	-1.34409173809567
H	6.14737092770877	3.80937026879606	-2.19486180443570
C	4.99529808030464	2.66068681461799	-0.76942833209621
C	2.86854188099477	4.04934197271266	2.12068876638823
H	2.41113576423543	5.04512013639956	2.25897182642416
H	3.46617483466320	3.83767039413819	3.02773894457673

H	2.08166808756708	3.27550633553420	2.10322192530492
C	5.48419339517946	1.32385200535909	-1.25358390805505
H	4.63920945031853	0.63041061760384	-1.44213383427671
H	6.12506796727957	0.84028428963270	-0.49259093926965
H	6.07015820494453	1.43108273600656	-2.17914178826094
C	0.09178043836839	-0.31235354594487	2.05078993389886
C	-0.38930602127239	-1.60311551134477	1.71952074541142
C	-1.73670598975293	-1.90394200276398	1.98964393694184
H	-2.10196637527877	-2.91358984904066	1.77480844755095
C	-2.60844597322324	-0.93629343610333	2.51255173574005
H	-3.65865784688242	-1.18823040052067	2.70578881146044
C	-2.13228298511531	0.35850976376574	2.77353342883763
H	-2.80766985035019	1.12576725768990	3.17534536565089
C	-0.77874336165365	0.68572070323520	2.55795082269299
C	0.55491118369231	-2.62114184098171	1.14667377421406
H	0.03543407995817	-3.56242155666767	0.90738063987633
H	1.37866104353749	-2.84393337064637	1.84471199734344
H	1.03385829002947	-2.24043994048339	0.22361986930535
C	-0.24826163747304	2.06103049404648	2.86107425600289
H	-1.01502429625100	2.69227950412149	3.33896137100507
H	0.07109099376245	2.54018383416755	1.91596490507036
H	0.63868089446682	2.02975752550023	3.51831011297476
N	-0.66683595879001	-0.65014879111600	-3.11122646101348
N	0.72305596616906	1.63359291747726	-4.04350340041048
C	-2.05037407744714	-1.76927470005708	-4.82806572757891
H	-2.69467285978831	-2.04781548439819	-3.98205357095748
H	-1.59926759765074	-2.69947958393104	-5.22472375558212
H	-2.68286201672226	-1.35191908298230	-5.62863772666955
C	-0.96852340550194	-0.78513074700415	-4.41193620888980
C	-0.35654858879787	-0.02785682711471	-5.46112982475159
C	0.33836591982551	1.19357256500104	-5.25526837807070
C	0.61139028975429	2.06585582324299	-6.46995241237551

H	1.04417213276561	3.03440369614778	-6.18040619539415
H	-0.30669181603371	2.25039330554480	-7.05558495589353
H	1.33065683292509	1.57093998560760	-7.14544970983082
C	-0.64469753667649	-0.45096360413955	-6.89272458289600
H	0.16485669435228	-0.14852440532060	-7.57659081350090
H	-1.58072498376389	-0.01301777808564	-7.29903313057973
H	-0.73749528597243	-1.54592656852934	-6.98581857591649
C	-1.47084707380385	-1.35093811763882	-2.16371846346278
C	-2.56704063408184	-0.67241307524251	-1.56926053420800
C	-3.48125266298455	-1.41581312810190	-0.79960464728605
H	-4.34402789975905	-0.90165129556386	-0.35652147381670
C	-3.30224256348291	-2.79328423137824	-0.59713893437867
H	-4.03043611553599	-3.36193077853467	-0.00549216070807
C	-2.16091902197423	-3.42761395889133	-1.10971131327925
H	-1.99041666804995	-4.49535120319569	-0.91709005816903
C	-1.22371993525369	-2.72053057186026	-1.88885721718370
C	-2.74543053855387	0.80536652335824	-1.79440242733207
H	-1.82191042170826	1.34452333104858	-1.49597540062755
H	-3.59708658071655	1.19174027808741	-1.21012494057853
H	-2.92360028793413	1.03696217286917	-2.86115252414505
C	-0.01472944348959	-3.41123875696264	-2.45868401319224
H	0.05912600742868	-4.44454139922831	-2.08391624793939
H	0.91278085963063	-2.86727653384144	-2.17935902306822
H	-0.03899555934443	-3.44748741274185	-3.56207005857060
C	1.03455167305957	3.00372406025779	-3.87633693021675
C	2.38944383536608	3.38512630884705	-3.67361454801001
C	2.68939318592833	4.74896704067242	-3.47689822661797
H	3.73367260019008	5.04517638846917	-3.32327098960470
C	1.67092574623070	5.72238939779922	-3.47090853272362
H	1.91872751722575	6.77912550299632	-3.30904710460528
C	0.33412669303845	5.33334344694774	-3.68535248166285
H	-0.46367229804210	6.08852124711253	-3.69207167358387

C	-0.00485281195020	3.97938493221180	-3.90101608648348
C	3.44835955296530	2.32288353877045	-3.71094855628129
H	4.45912343751409	2.75300681883860	-3.65401804351665
H	3.37219572664207	1.71888548097961	-4.63009148339675
H	3.32147025146930	1.61328648199304	-2.87078021548842
C	-1.42882748964453	3.56629154775281	-4.17446071651305
H	-2.13204894423201	4.38238496094408	-3.93979509350472
H	-1.70055049299275	2.67510086112694	-3.58436342225899
H	-1.57540048297828	3.29434354513005	-5.23672739502739
N	4.38710660508557	-2.43797485447366	-3.67541077859560
N	4.21830564628666	-3.58482066326222	-1.10678117798950
C	5.54723455616636	-3.78058228836768	-5.40330448394652
H	5.29263372704851	-2.93179580549994	-6.05361134469004
H	5.08596876081587	-4.68793077512023	-5.83553616270721
H	6.64139278922070	-3.93107812526158	-5.44532386395338
C	5.07079445045390	-3.56497202902014	-3.97608196413795
C	5.40653160060502	-4.55728946926823	-3.01290880871526
C	5.09219940299866	-4.47889575063843	-1.62749440103153
C	5.80301079222321	-5.43269080720841	-0.67728386070036
H	5.81652589261991	-5.02884763763599	0.34667195025336
H	6.84496601278748	-5.60020463311808	-0.99493926574150
H	5.31225032544052	-6.42403566878000	-0.63003446254096
C	6.21196111610974	-5.75652991992064	-3.49489549591961
H	6.12581515728972	-6.61374948380886	-2.80965441204845
H	7.29448155353104	-5.53860929837943	-3.61117994722219
H	5.85721473681086	-6.10991048868552	-4.47819959337792
C	4.31332306638459	-1.41267574993775	-4.65830814380948
C	5.41591445299654	-0.53233667992905	-4.82471637601892
C	5.36168526030618	0.42558592194024	-5.85549469525879
H	6.20874947453996	1.11134485342202	-5.99097663669262
C	4.25124083282510	0.50105040662186	-6.71321397272855
H	4.22752221099246	1.24320289995419	-7.52096543768047

C	3.17074403237946	-0.37908366048987	-6.53325052723889
H	2.31462899828704	-0.34738529587793	-7.21739215919487
C	3.17599969079550	-1.33625037164344	-5.50232690751612
C	2.04485123330825	-2.31111522425482	-5.31725142032894
H	2.41527507561584	-3.34648789773064	-5.21426774215565
H	1.33660112489957	-2.26679019771249	-6.15869523975273
H	1.48903105910891	-2.07426410490967	-4.39266685588169
C	6.64330718145318	-0.69260618900990	-3.96266664904245
H	7.28417072677473	0.20410357236589	-4.00948480793097
H	7.25046226785623	-1.55806113301378	-4.29210772818564
H	6.36938025309290	-0.88585831142320	-2.91081650962760
C	3.84756845753640	-3.76284587099663	0.25598592238211
C	2.96408904662164	-4.82110426327290	0.60308742801378
C	2.69929146984698	-5.05740543296991	1.96526817360712
H	2.01841243042965	-5.87379508772012	2.24123235689773
C	3.28350687548713	-4.26105260168436	2.96356111081208
H	3.07699411486070	-4.46226767079539	4.02223434562041
C	4.12644349719261	-3.19705910545842	2.60052603536628
H	4.59057033352510	-2.57764415585809	3.37492156136834
C	4.41592038041241	-2.92524832092516	1.25014766020681
C	2.34374696182028	-5.68230768742176	-0.46877100009725
H	1.42795441433728	-6.17376399032002	-0.09808545762591
H	3.03351624206303	-6.47745082180396	-0.81135162402181
H	2.09106557831261	-5.07935927646763	-1.35706481577941
C	5.35529697672745	-1.81787175578539	0.84967810108623
H	5.79275400556350	-1.32864320833711	1.73400551229589
H	4.82015250886131	-1.04470629835896	0.26568179994881
H	6.17360441016127	-2.19353785508046	0.20847656779573
K	0.97961195543117	4.06255610446704	-0.52477553248185
H	-1.75636745812425	3.65546683797047	-1.32792598060788
O	-1.36931595753491	5.06406869440529	0.16415491959090
C	-2.04682104407449	4.96839439843862	1.43676022690349

C	-3.10842708242983	3.88725688816876	1.23807024173207
C	-3.56146125495407	4.18182097258650	-0.20212899121462
C	-2.23718158645608	4.55039318144557	-0.88287135929637
H	-1.29641215671845	4.73135314812666	2.21100291629471
H	-2.51270105010465	5.94459145112925	1.68920350419080
H	-3.92453315617516	3.94375690492047	1.97834646922576
H	-2.64897087865348	2.88281001947590	1.29895018657564
H	-4.26021542409222	5.03863580062590	-0.21814658677608
H	-4.05634993540758	3.32615845737040	-0.69035905942309
H	-2.34458367159630	5.31717706857610	-1.67146674355409

Optimized structure of NP solvated in THF - additional THF coordinated to one Fe

Fe	1.62902784047546	0.24124058730507	-0.02755236615603
Fe	0.30460210167062	-0.21757915892377	-2.31744204499390
Fe	3.32862723723521	-2.05925986040347	-2.20191795783090
N	0.08117379135343	0.92659857160515	-0.84388048780784
N	1.99486806281071	-0.87423320595039	-1.60389232961224
N	3.14570166120792	1.36994778802357	0.79177473158112
N	1.03649690078725	-0.02468635469673	1.92846401673084
C	5.00351549425164	1.86966725269017	2.35065237009785
H	5.18565646584415	2.72336236712602	1.68203942660307
H	5.89425208656225	1.21429358731381	2.31275411691991
H	4.92884062966762	2.24295263750363	3.38664064869577
C	3.74697822855931	1.10772269276005	1.96145689040253
C	3.23945174016079	0.17938139892101	2.92928512300491
C	1.88303065512696	-0.22735449028054	2.95740853377314
C	1.33171909328869	-0.85174797926017	4.22677000047393
H	0.25082028327508	-1.03768483116343	4.13138113031388
H	1.50438021075299	-0.21450980744155	5.11343173387008
H	1.82974173955326	-1.81810282877219	4.42031945308350
C	4.11546191701208	-0.13754609525024	4.12792012681806
H	3.81757257443022	-1.08333246317743	4.60437921634700

H	4.07177966885986	0.64866606955091	4.91153772530450
H	5.17546317531106	-0.24995595972517	3.84446331861712
C	3.71902068803281	2.34663521186241	-0.06688604635897
C	3.16592760513681	3.64989945342284	-0.13110262692025
C	3.81714799405140	4.61891335674987	-0.92069524091743
H	3.40820895267576	5.63789196907776	-0.95448414763688
C	4.96414232629190	4.29550082451046	-1.66569724979230
H	5.45505565725165	5.05964916602299	-2.28134123285131
C	5.46010542296938	2.98047566835001	-1.64396394278380
H	6.32964355376295	2.70582955230878	-2.25421392249119
C	4.84941212988397	1.99180550991715	-0.84939120842516
C	1.91393173909660	3.97235635157465	0.63942621390968
H	1.45346886074454	4.90386716325324	0.27319274939538
H	2.11073254370664	4.08924882613906	1.72108217004444
H	1.19565268029464	3.13834796997997	0.53280636734623
C	5.37216764827042	0.58382555631372	-0.79673938255966
H	4.57275918858360	-0.13399427404607	-1.08161722612758
H	5.69280918201521	0.29907165246836	0.21983982606073
H	6.21853561688558	0.45236867669963	-1.48456166145986
C	-0.32921034825850	0.21668984246115	2.18349350886942
C	-1.31171670764365	-0.68001262096849	1.68480093851796
C	-2.67116877824419	-0.35428320418228	1.83684462529849
H	-3.42274709276727	-1.04984980390561	1.44805557280575
C	-3.06960463966066	0.83764472218587	2.46950501399851
H	-4.13497704631214	1.08159880448675	2.57100062272170
C	-2.09116104704428	1.70045495803218	2.99702826102081
H	-2.39294510137195	2.62037995262370	3.51678590105848
C	-0.71611251620445	1.40376050862876	2.87510351249675
C	-0.86541406920094	-1.95306439519550	1.02517810457658
H	-1.71486173382814	-2.61239188245257	0.79206817642382
H	-0.14269792962145	-2.49655886158335	1.65993353949115
H	-0.33654966335028	-1.72616377511904	0.07835819477842

C	0.32548533827382	2.32949915831162	3.45641063968413
H	-0.07649740884019	3.34932328746958	3.58743481717491
H	1.21581369242965	2.37814132905893	2.80942484815764
H	0.67122465686382	1.98281572117025	4.44926979580808
N	-1.21825547120592	-1.42343279051633	-3.06817409914531
N	-0.02420972829560	0.97100018718800	-3.96661589678545
C	-2.35168160878167	-2.63734135259901	-4.91327911460791
H	-3.10890119503037	-2.87843813559656	-4.15218049530980
H	-1.86834318591617	-3.58560606209992	-5.21650424399879
H	-2.86160040086254	-2.23872708056872	-5.80651457863550
C	-1.33048340511354	-1.64322467494447	-4.38480687586016
C	-0.57922063647385	-0.92259808647072	-5.37682219898174
C	-0.13541893565541	0.41119991229345	-5.18209571025475
C	0.16423243084086	1.25826404546076	-6.40524680305026
H	0.47374449718957	2.27328897637645	-6.11045123105318
H	-0.71376788476434	1.33515657072201	-7.07324416585219
H	0.97668810877092	0.80883562587442	-7.00439340052546
C	-0.50936089282216	-1.50122545742281	-6.77952856799358
H	0.44474663737744	-1.23662519895397	-7.26860545918146
H	-1.31981826704486	-1.14494374252266	-7.44979263684155
H	-0.56108944717054	-2.60273555062929	-6.76944626944240
C	-2.13805922107292	-2.04870133823082	-2.17306309769249
C	-3.16880649333969	-1.25862041372783	-1.59414869150355
C	-4.13797199934308	-1.88504448332743	-0.78679715604119
H	-4.95132005963905	-1.27491869721073	-0.37185889523852
C	-4.07440031163267	-3.25816874643135	-0.50442855129609
H	-4.83376549926304	-3.72957478973466	0.13189190483334
C	-3.01301695671293	-4.01443375650104	-1.02311702600405
H	-2.93180165947673	-5.08367175806300	-0.78534768241595
C	-2.03687199996787	-3.43248423803629	-1.85754012264537
C	-3.23432054701617	0.22078851596446	-1.85938614036979
H	-2.22016356995408	0.65756790826650	-1.75938604665690

H	-3.92798721036450	0.71154430084499	-1.15449930333518
H	-3.58016895687078	0.43536470325532	-2.88520695445482
C	-0.92468258587588	-4.28500827597023	-2.40623203061178
H	-0.77237142488734	-5.17623742038643	-1.77469751437190
H	0.02446233866988	-3.72905664407563	-2.46053279845376
H	-1.15249546186462	-4.64505200366111	-3.42765718680396
C	-0.16397722671259	2.35937914572556	-3.77248852662453
C	0.94223165822351	3.10059173658685	-3.26411856597905
C	0.75579323551344	4.44774857761613	-2.90172187485871
H	1.61037031829784	5.00894023665706	-2.50349447539442
C	-0.49612595078586	5.07494952310321	-3.05668692831073
H	-0.62975323502159	6.12568950222255	-2.77129991168374
C	-1.56680007614648	4.35408778939138	-3.61472566923070
H	-2.53777330772111	4.84531062089514	-3.76963289337839
C	-1.41902498001448	3.00037649589377	-3.99564446943617
C	2.28743331581254	2.44116989248076	-3.20103346217728
H	3.06361054092557	3.13488307235810	-2.85341308399219
H	2.57245324872034	2.06178550327637	-4.19614661926950
H	2.27206930395599	1.56124663918531	-2.52889053857132
C	-2.56690278993654	2.27867845309231	-4.66151369469961
H	-3.53921679928755	2.64156281556270	-4.28478271211945
H	-2.50995992758198	1.19002203024643	-4.50737731320965
H	-2.55622612852031	2.44953227284764	-5.75588626957713
N	4.89651735776715	-2.02592675806543	-3.46241758894135
N	4.26179612776916	-3.44848343317384	-1.09999809707580
C	6.16476885269910	-3.26497907063535	-5.17344722545643
H	6.12119723152376	-2.28839800852768	-5.67880010335611
H	5.65105314054647	-4.00174986449817	-5.81979887649427
H	7.22317350634476	-3.57674678814761	-5.11281043673679
C	5.49176405198031	-3.20024227199701	-3.81084417834946
C	5.51047098319043	-4.35506787182587	-2.98823389217964
C	5.05991088812786	-4.39063207153472	-1.62660534287648

C	5.45914529217333	-5.58739754988184	-0.78338237199935
H	5.19981037696223	-5.43847620388006	0.27521248931998
H	6.53930833765382	-5.80407706268004	-0.85792276339123
H	4.92810231365089	-6.49437982746295	-1.13268733610574
C	6.16353936046736	-5.62172166859227	-3.51973538720851
H	5.60236750221159	-6.52734991861433	-3.22624204733675
H	7.19885836952274	-5.75672355887178	-3.14455160023061
H	6.22156787220193	-5.62910094147024	-4.61831157979435
C	5.43890561672860	-0.81855319061490	-3.97007143239975
C	6.79692952066202	-0.47588514617201	-3.68555791742239
C	7.30831479980550	0.74926781246731	-4.15720233771455
H	8.34914932113881	1.01042485490069	-3.92186636111270
C	6.51241272550398	1.63885743883202	-4.89545389208247
H	6.92414789300254	2.59332747736718	-5.24613543944918
C	5.18198060563566	1.29158283394628	-5.17719612113676
H	4.55011700311896	1.96777209230068	-5.76845234132790
C	4.63384636317272	0.07333755020002	-4.73226269998406
C	3.23261885536720	-0.31171096622005	-5.11079157835404
H	3.22312662183040	-1.27789983610524	-5.64421552109249
H	2.77749199225850	0.44870601495586	-5.76403856759536
H	2.58947874567229	-0.42218962253376	-4.21542510585406
C	7.68522550262962	-1.38930202283247	-2.87482766529900
H	8.51847627294955	-0.82250339186286	-2.42478254273538
H	8.12802671481235	-2.19322999019556	-3.49270767546745
H	7.12048744357402	-1.88807026004695	-2.07086015368593
C	4.02431677834236	-3.41834621232652	0.30093964020820
C	2.75428784380003	-3.79780864793056	0.81382270079626
C	2.56144854058285	-3.80227488432961	2.20875080188884
H	1.58416801584856	-4.10925804973337	2.60480407520365
C	3.59627042801100	-3.44670071624168	3.08730903348425
H	3.43664352134450	-3.47943486378176	4.17218966379352
C	4.83427481680897	-3.03941358969391	2.56809480591996

H	5.64288226446551	-2.73861956872574	3.24669197646601
C	5.06621700851103	-3.00880112426037	1.18034550259826
C	1.65645310152095	-4.22757233471451	-0.11980080472057
H	0.73403672219527	-4.46940307552530	0.43293538925798
H	1.95136120723343	-5.11189035680360	-0.71192275861435
H	1.43047154345087	-3.42311945591079	-0.84331355900933
C	6.40640305007109	-2.59024822271874	0.63283488421700
H	7.00714290336120	-2.07828463203492	1.40329401783393
H	6.28035190746294	-1.91207409704549	-0.22681577183080
H	6.99254075710475	-3.45465375850977	0.26637975971464
K	-1.49306943421591	2.92132280784118	-0.31597083787276
H	-1.67682625278036	7.12713275335206	-0.22302752531369
O	-1.62703894850184	5.31773752094473	0.84145656370999
C	-1.99300777415964	5.52870644204488	2.22917671965015
C	-1.55651935460289	6.95952746155863	2.57891014064433
C	-0.42122101815861	7.21103962602955	1.57070570394071
C	-0.95581799718303	6.49165377273695	0.33349986659852
H	-1.46056979836753	4.77948894994097	2.84630465719157
H	-3.08035603904200	5.36486706120818	2.34815623626862
H	-1.23803662487999	7.05356261266046	3.63097377038771
H	-2.38440031649077	7.67191027935286	2.40652915832727
H	-0.22387358586520	8.28155445535826	1.39059996866629
H	0.51761260562120	6.73572687887156	1.91198190785336
H	-0.17222495500425	6.16161677487226	-0.37076116965231
H	1.43288287900095	-5.60577582863360	-3.23133584045339
O	2.14944811155250	-3.65784582959063	-3.38024220243979
C	1.57088071693419	-3.29162704739433	-4.64732699105156
C	2.42286403614122	-4.02789207500590	-5.68083878143069
C	2.79633358895273	-5.34275677365470	-4.94354802829225
C	2.37817614155216	-5.08146991028201	-3.47772766067140
H	0.52115952448251	-3.64409815421882	-4.67871059335672
H	1.56069334612688	-2.19649139983173	-4.71356629984766

H	3.32916921836976	-3.44122527732282	-5.91168214502315
H	1.87642066591433	-4.19824288817801	-6.62391209156220
H	3.87570103104745	-5.54873779815969	-5.01946902047357
H	2.25554852746845	-6.21300530862593	-5.35374898071266
H	3.14361374909042	-5.34853645620175	-2.73674641881811

Optimized structure of solid ($(L^{Me3}FeCl)_2$)

Fe	-0.22844079015587	0.50338190796563	-1.25003827765915
Fe	0.22907327351116	-0.50312503911204	1.24946479189359
Cl	0.54469370152575	1.70779071335196	0.59886190470114
N	-1.78921091808408	1.33667287941137	-2.05609310556803
N	0.90570288648585	0.72703379335100	-2.81280740570817
C	-3.33117722600156	1.78046549872149	-3.92155702627675
H	-3.80885576812450	2.47933112739908	-3.21819021531743
H	-4.02843450539057	0.93056308400863	-4.05519495201709
H	-3.22593593263001	2.27920194942519	-4.89780078152842
C	-1.99272014097803	1.30265673551227	-3.38605832309027
C	-1.00970672192686	0.87317706093533	-4.32787105076753
C	0.38730528856196	0.76549932099996	-4.05439796631663
C	1.34152652368371	0.72855137140776	-5.23529719741601
H	2.34397962226239	1.07502531512666	-4.94204215433542
H	0.98260410880057	1.35243919813730	-6.06918411787271
H	1.45502228049130	-0.30405813822988	-5.61861374144405
C	-1.46235361296859	0.65179850865465	-5.76367133847213
H	-2.49597873965642	0.27020814815433	-5.80382995825255
H	-0.83691280908068	-0.10247321435479	-6.26912931723716
H	-1.43196881108351	1.56835600140546	-6.38999277554511
C	-2.76523362947631	1.93921826695574	-1.20700226622399
C	-2.64326622000736	3.32183472352706	-0.90404081000620
C	-3.64940214299895	3.93126635175182	-0.13229898940777
H	-3.56357741576318	4.99960771396209	0.10542548405804
C	-4.76587422479723	3.20018312179975	0.30178255690527

H	-5.56100561497939	3.69593214513500	0.87231063735263
C	-4.86165671225402	1.83480729192705	0.00088090380303
H	-5.73199538523460	1.25762671941093	0.33327662195007
C	-3.85384417948565	1.17049847118251	-0.72385776302023
C	-1.46861336824463	4.11102296786194	-1.42020539855490
H	-1.57536366491788	5.18295908378449	-1.18443145274020
H	-1.34735821385116	4.00322578332483	-2.51310852312810
H	-0.53178955873366	3.74646170121845	-0.96279238106979
C	-3.93144658446938	-0.31489927446680	-0.96561756557674
H	-4.95491171740507	-0.68935355307389	-0.80075758539665
H	-3.26627131957918	-0.85179222269702	-0.26332252829592
H	-3.60511066704599	-0.59449800810823	-1.98139196230114
C	2.31954766483808	0.79889467813702	-2.63426652226563
C	3.09908787967445	-0.38436374471780	-2.67764861630899
C	4.50022429078848	-0.25401707932348	-2.63387441603447
H	5.11135909166273	-1.15969628826587	-2.72037642671587
C	5.11335673317941	0.99694441654636	-2.47961044113288
H	6.20708600857661	1.07385804525016	-2.44343744990741
C	4.32178218359349	2.15076328182016	-2.37318631616885
H	4.79397097898968	3.13334356700283	-2.24368687782772
C	2.91978098903490	2.07504667853582	-2.46430429449375
C	2.45253253885745	-1.74308497002323	-2.75749741944124
H	3.18939798286683	-2.51546324839928	-3.03124613190671
H	1.61826147655870	-1.77160020973221	-3.47889642088169
H	2.02747925935679	-2.02331301278520	-1.77532673685953
C	2.06419601634786	3.31313190714893	-2.39821034735917
H	2.68317611526338	4.22573641152944	-2.39546270021241
H	1.45382700669697	3.30512247705961	-1.47739354619412
H	1.35509847262497	3.37108126764287	-3.24309212845276
Cl	-0.54338482020311	-1.70750254694046	-0.59949967325488
N	1.78924159385432	-1.33700388283073	2.05574712830357
N	-0.90538544072527	-0.72522914313016	2.81243997944639

C	3.33114163325896	-1.78088077801235	3.92132324292135
H	3.80890007163680	-2.47920028783570	3.21747492937855
H	4.02856251174376	-0.93132237861709	4.05621044981431
H	3.22534830669329	-2.28054135339867	4.89705355866915
C	1.99300718211245	-1.30238477043398	3.38567725139291
C	1.01027678963513	-0.87195158850301	4.32731140518818
C	-0.38675630670799	-0.76374537870564	4.05392384346077
C	-1.34048298149336	-0.72633189697371	5.23523901628530
H	-2.34398444426937	-1.06994284043401	4.94229617843354
H	-0.98245125931315	-1.35231727469181	6.06796924633783
H	-1.45102828431965	0.30595783652637	5.62026824681255
C	1.46327186563504	-0.65068839758886	5.76301362910199
H	2.49694057563488	-0.26915807799089	5.80280095991996
H	0.83805810352355	0.10350921074632	6.26883593850947
H	1.43307482583784	-1.56731670909776	6.38924319134050
C	2.76444211876653	-1.94089877775901	1.20668183511005
C	2.64103937073728	-3.32343447416256	0.90409089133577
C	3.64647672161925	-3.93413374738683	0.13241103726100
H	3.55943218015511	-5.00244124550842	-0.10503666399016
C	4.76373838329971	-3.20445166046398	-0.30188986655687
H	5.55842422390898	-3.70097437993187	-0.87235787293179
C	4.86103531790019	-1.83909641952763	-0.00122878165741
H	5.73197370085540	-1.26288875383976	-0.33372630071301
C	3.85393772348542	-1.17351210505135	0.72331467651361
C	1.46566039821850	-4.11138799531806	1.42050141564032
H	1.57234052530214	-5.18374028636664	1.18658238160811
H	1.34355127685299	-4.00172849726128	2.51311283760919
H	0.52929549041292	-3.74740121597600	0.96168469034035
C	3.93334056325473	0.31179967319045	0.96490419010727
H	4.95555375174154	0.68602241956924	0.79213524509825
H	3.26265879936012	0.84907161278819	0.26819083297683
H	3.61525354369624	0.59130229324747	1.98338300947149

C	-2.31934728971269	-0.79717736984343	2.63447186721019
C	-3.09888443176401	0.38607187636839	2.67815465706966
C	-4.49999320756530	0.25563930493095	2.63492417508080
H	-5.11135487609949	1.16116006324876	2.72152138585980
C	-5.11309572177099	-0.99537249951214	2.48087538312222
H	-6.20682997087790	-1.07240922662745	2.44510055522100
C	-4.32154521107620	-2.14916440129983	2.37403057366313
H	-4.79376404602317	-3.13173339543616	2.24458443003290
C	-2.91947814054579	-2.07336357248102	2.46460639932489
C	-2.45196506721069	1.74457588471472	2.75814859930194
H	-3.18961611131631	2.51788029995894	3.02710799259357
H	-1.62112032448905	1.77397587648942	3.48350991418906
H	-2.02222995372694	2.02287779014364	1.77750240396700
C	-2.06388947238732	-3.31137761095468	2.39769929570399
H	-2.68278481319321	-4.22403615691796	2.39572709500408
H	-1.45442775201139	-3.30346693669252	1.47627040796980
H	-1.35383940123987	-3.36911679161263	3.24179260744713

Optimized structure of the ($L^{Me3}FeCl)_2$ precursor in THF

Fe	0.00201924858200	0.98938678626022	-1.57366689398387
Cl	1.37533693091154	2.14734975466731	-0.16606196293742
N	-1.56611846707218	1.78232688747176	-2.39097539389296
N	0.61287065829002	0.30114320710064	-3.27865301912812
C	-3.06263334328284	2.72647896478910	-4.09462601140339
H	-3.72156808607706	2.87453827057767	-3.22505834442338
H	-3.62724352418871	2.18393271774273	-4.87300008090620
H	-2.82046788282341	3.72426282181276	-4.50684223098167
C	-1.80152434550587	1.98228179290773	-3.70028036538906
C	-0.92677860184517	1.51560101461267	-4.72605712746530
C	0.17770841888166	0.64081052088060	-4.50664524524452
C	0.89449347237655	0.05151353018262	-5.70639860208118
H	1.60623752650353	-0.72927742357317	-5.39657818509693

H	1.46466981053429	0.83054624111836	-6.24690041549863
H	0.18472824104551	-0.38856449381544	-6.42864021064326
C	-1.25101858479030	1.90037046673880	-6.16151063948037
H	-1.73347376302952	2.88972085203371	-6.21985765296604
H	-1.93490014881573	1.18042388613208	-6.65784608339411
H	-0.34305761698249	1.95798085147321	-6.78391783260708
C	-2.51179606453361	2.10225547496050	-1.37873844016984
C	-2.40407501945788	3.33411080809074	-0.67683202805074
C	-3.27725734764855	3.56250242099580	0.40570387415918
H	-3.19824298483802	4.50751437276632	0.95950332863476
C	-4.24451978486564	2.61212570266194	0.77314682754608
H	-4.91487585388781	2.80897434822736	1.61914422138744
C	-4.35815765111686	1.41399110437184	0.04787986805595
H	-5.11779279150420	0.67143323830512	0.32628048238285
C	-3.49845378557422	1.13778655134160	-1.03165492654332
C	-1.38050245122746	4.36193697141509	-1.08938813253427
H	-1.52100772454897	5.30211880832599	-0.52990360671508
H	-1.44525454856325	4.58463396351741	-2.16983749886811
H	-0.35553749336511	3.99039804205983	-0.89879886072510
C	-3.58132836797580	-0.16247636692290	-1.79019495364491
H	-4.40974853225541	-0.78693409699172	-1.41650262192287
H	-2.63931025004567	-0.73298426768520	-1.68542654945131
H	-3.72805102591595	0.00119055129138	-2.87333118461455
C	1.58600951476996	-0.71283024605570	-3.06174772026443
C	1.15928853788743	-2.06845039991127	-2.99509787637642
C	2.10867598824953	-3.05860713929083	-2.67978464524283
H	1.78522925387674	-4.10641322198909	-2.62020062482479
C	3.44971567177924	-2.71940515305440	-2.43212065083364
H	4.17690779456720	-3.50064880237043	-2.17779558484429
C	3.85974217901571	-1.37893869290973	-2.52464639402137
H	4.91176924165455	-1.11416511763610	-2.35383807305428
C	2.94613518844561	-0.35644525551590	-2.85092081687851

C -0.28846009189271 -2.41403759896430 -3.23733388748100

H -0.46365780621104 -3.49613027965229 -3.11781881910232

H -0.61601599440763 -2.11922313052565 -4.25138878872273

H -0.94498023792350 -1.87189231237597 -2.53140712359661

C 3.39243679264087 1.07737994953219 -2.99016782397938

H 4.49166798193836 1.15278038673900 -2.93804434522951

H 2.95923599103948 1.70384567098979 -2.18717900180406

H 3.05707117872114 1.51018347738281 -3.95005609123231

H 0.53165718433903 -2.29168285995419 -0.39660385037476

H 1.56473352655851 -0.88118624415041 0.02298253647480

H 1.35882981154303 -1.85280826366786 2.23714735675531

C 0.60645211449269 -1.39149811385812 0.23222101557193

C 0.40373129622402 -1.64554985367139 1.72556551465310

H -0.26879911325516 -2.50823687028987 1.88669916083165

O -0.47384014604598 -0.47326862219105 -0.16100272517915

C -0.26791811384793 -0.34393158694535 2.19580321543746

H 0.47881413507038 0.46483173130084 2.29184319304039

C -1.20534484914585 -0.03708291055860 1.03553977880795

H -0.80692315687433 -0.44907707605024 3.15244288334058

H -2.14700755151390 -0.61405323677415 1.08728104456287

H -1.44327458708289 1.03173749657383 0.91232564216371

Optimized structure of $L^{Me3}Fe(THF)_2$ cation

Fe -3.09946993262391 4.94307540707148 0.72597704017879

N -3.08119044546830 6.87242392092491 0.67578028928904

N -4.93924371200760 4.95016050694358 1.30491574529203

C -3.74892239257727 9.22808817936091 0.77058452604072

H -2.71537082513867 9.37404708276652 0.42098473738462

H -3.86770178191630 9.77639002529896 1.72217595367237

H -4.42722099728281 9.69725664217687 0.03580215726630

C -4.06182776154538 7.75455052346640 0.94383475063739

C -5.35442794515191 7.34987114149099 1.38349259412054

C	-5.74999649762360	5.99177534242190	1.55958355835656
C	-7.15360187535521	5.70417911545159	2.04664985191884
H	-7.32399218279899	4.62377782568290	2.16948322444502
H	-7.90574834046375	6.09521098607065	1.33718848306011
H	-7.34547481695825	6.19920476537305	3.01568016930704
C	-6.39994267867818	8.40866153848867	1.69178997802820
H	-6.03287602134733	9.42690581620243	1.50379296700283
H	-6.72424149125783	8.37435949937623	2.75007262865354
H	-7.31332601051189	8.27805339329054	1.08036198705324
C	-1.78492317021168	7.25776309393081	0.23482974663138
C	-1.52148902273492	7.34013225859275	-1.16133458409487
C	-0.20182599134734	7.60648286698800	-1.57604824648543
H	0.01529592896109	7.66791302926803	-2.65041800778003
C	0.82992342690536	7.78137044036858	-0.63811714571412
H	1.85469533635918	7.97226125306810	-0.97971743139702
C	0.54603298597053	7.72871547210036	0.73683550558802
H	1.34668002525445	7.89237782858306	1.47002704227222
C	-0.76284875868613	7.48593798739061	1.19780667022700
C	-2.63628087275046	7.14426992587074	-2.15824426689984
H	-2.25776560073749	7.21312773037517	-3.19131952521896
H	-3.43021825725098	7.90261717697430	-2.02850238948473
H	-3.11840049858714	6.15833336123671	-2.02864252381515
C	-1.10211520259779	7.53341229818422	2.66648156443457
H	-0.20482469233870	7.39043834952472	3.29122666703018
H	-1.85493469433840	6.77321301834319	2.93707511956002
H	-1.54189385011353	8.51514929083617	2.92847978167066
C	-5.34542688674788	3.59578505401455	1.46035780516217
C	-5.10227918080010	2.93363193756189	2.69605771385301
C	-5.40374223363342	1.56038150474173	2.78645539266348
H	-5.21695954636463	1.03637114762738	3.73286962422185
C	-5.92522501401158	0.86026787121548	1.68539045036123
H	-6.13906268142829	-0.21236832796903	1.76917120903365

C	-6.18815723356445	1.53712349004941	0.48259508096190
H	-6.61971337614931	0.99584666470138	-0.36959564076842
C	-5.92130821258870	2.91425748681693	0.35273178712536
C	-4.54099951211587	3.69576447812579	3.87029724175313
H	-4.37152228651803	3.02672518081445	4.72988335745139
H	-5.22626947975370	4.50261223174990	4.18950192843890
H	-3.58285212996701	4.18155129414370	3.61190373846435
C	-6.29035822460991	3.67385019679223	-0.89697159156267
H	-7.24396937258295	4.21718681969300	-0.75142607014046
H	-6.41859525127980	2.99496323597217	-1.75637939959219
H	-5.53379681299176	4.43670968842046	-1.15018739735519
H	-1.01499815092464	4.58366471441940	3.53768880864678
O	-1.84746917259295	3.74185917070294	1.81967561526710
C	-1.92611710606076	2.27704256923040	1.73623247794102
C	-0.98429549371126	1.78846056346953	2.82847119792539
C	0.12509448702713	2.85547624252414	2.79807379968424
C	-0.66170758543302	4.14657303493588	2.58680979536436
H	-2.98059493495617	1.99146758719563	1.87298072905620
H	-1.58763554563958	1.97140178134019	0.72905903459544
H	-0.61232449699633	0.77006202998070	2.62739531412682
H	-1.49939467886222	1.78572023791690	3.80652143474345
H	0.80889331008259	2.67630638819933	1.94822614767416
H	0.72422208811147	2.88719584975033	3.72328827406261
H	-0.12919395882903	4.91483217435844	2.00208582747810
H	-3.67418213352278	3.63898655966474	-2.50803430358577
O	-2.35343549715900	4.00395559704846	-0.93504162037425
C	-0.91416659988149	4.00893140626600	-1.23224428158884
C	-0.80951565295382	3.33257716311729	-2.59264063169461
C	-1.93909865217880	2.28880540115584	-2.52229812419404
C	-3.05212525885968	3.05198274033467	-1.80910016425268
H	-0.57378534332004	5.05561197008363	-1.20831426133342
H	-0.39893866071654	3.43453438019621	-0.44035687757020

H	0.18489828149052	2.88469399251559	-2.75531480865737
H	-1.00033487250829	4.06294337647986	-3.40005435883554
H	-1.62126840129096	1.41734562406972	-1.92092854812541
H	-2.25964994061213	1.92641072037574	-3.51319953843378
H	-3.70077444564337	2.42554640170240	-1.17440850822238

Optimized structure of $L^{Me3}FeCl_2$ anion

Fe	-0.26413556412876	0.55121259857010	-1.58791773283789
Cl	1.18583678084411	1.60274345132494	-0.11559935756884
N	-1.64931569589778	1.66916567905092	-2.38789418854810
N	0.48708392421718	0.13575741253627	-3.34210660191044
C	-3.03288252702800	2.83449812149324	-4.05547692773566
H	-3.44627944679140	3.31896567287796	-3.15757436110637
H	-3.85766334325186	2.28902340868578	-4.55247558494211
H	-2.70263781669391	3.62399849526779	-4.75372770278050
C	-1.89007540271277	1.90139846629886	-3.69267818261773
C	-1.11729264682915	1.32553496237266	-4.74533111925965
C	0.04059849930961	0.51272731494531	-4.55551149012281
C	0.81931091624196	0.05956542311508	-5.77901141469131
H	1.75904379745988	-0.43442677899484	-5.48812196452190
H	1.06541007453465	0.91087085178246	-6.43825052409420
H	0.23545234849949	-0.65926238377714	-6.38491074952486
C	-1.53112868442847	1.64030348635574	-6.17614030231406
H	-2.61991809770542	1.78705169401296	-6.26617987944650
H	-1.27059071105886	0.82319880609060	-6.86849574575171
H	-1.05217854858733	2.55996546450592	-6.57496373542858
C	-2.44420536960407	2.24906097168924	-1.36122149170182
C	-2.05309939882584	3.49169613648646	-0.78864882524643
C	-2.80432825527886	4.00074299972139	0.28931412513103
H	-2.50445983839131	4.95663018374368	0.74003631592005
C	-3.92406845704177	3.31100728302532	0.78368707563709
H	-4.49508649152703	3.71983210783291	1.62709314297685
C	-4.31970083071624	2.10423577993802	0.18262400667858
H	-5.20770356135468	1.57201717633728	0.54998209131281
C	-3.59890309742719	1.55866539287800	-0.89810181159907
C	-0.86453071281891	4.24138280195759	-1.33532546834006
H	-0.78703322909324	5.24286769139669	-0.87865090102018

H -0.92802243056274 4.35957701776472 -2.43231682902591
H 0.06614009971871 3.68087138402156 -1.12206722720701
C -4.03366041737044 0.27449760722775 -1.55842142795897
H -5.00135202055967 -0.06813891706899 -1.15367782014551
H -3.27678194761576 -0.51512562553710 -1.38678567740994
H -4.13462256396687 0.39387968231014 -2.65250716508348
C 1.64841879627035 -0.66979419937275 -3.18521397787987
C 1.51539688330615 -2.08589741588298 -3.14846694654458
C 2.66385803063447 -2.86243322774964 -2.89685429160885
H 2.56671804075217 -3.95603692601762 -2.85964307697374
C 3.91883122483475 -2.26092633222179 -2.70352340053502
H 4.80253803819241 -2.88072545655444 -2.50491281604724
C 4.04055733443875 -0.86343957554790 -2.78228950567236
H 5.02318299113734 -0.38917634083281 -2.65473655558982
C 2.91876620502665 -0.04783260165156 -3.03020171705257
C 0.17141677150937 -2.72971017330901 -3.37991077304373
H 0.26435472378072 -3.82822859444552 -3.42741567548266
H -0.29023254960597 -2.37648382658499 -4.31992796014551
H -0.52325917972891 -2.46634369721167 -2.55903287106636
C 3.04957464065038 1.45059091633018 -3.13499531969670
H 4.11007383883049 1.75515860094703 -3.12083399707865
H 2.52989109803092 1.93363874582767 -2.28530728770211
H 2.58586879263873 1.83628116519146 -4.06115535294509
Cl -1.04602501425600 -1.25821288115292 -0.36279302264988

Optimized structure of $L^{Me3}FeN_2$(side on) cation

Fe 0.05542878166401 1.27805238253785 -1.97344196041532
N 0.28227767563502 1.03773724550954 0.02287422301634
N -1.51914362551737 2.02250189642271 -2.72127758441166
N 0.42215948273064 0.29024774409578 -3.56546334123644
C -3.14085224008695 2.83275370313436 -4.35796080309257
H -3.67226345969086 3.15263505284651 -3.44909084119339
H -3.81770925817680 2.21118973563806 -4.96922669883094
H -2.90006635024411 3.73513869482962 -4.94905206465335
C -1.87462179542747 2.08903031912812 -4.01250205020025
C -1.08542959056164 1.49454690725501 -5.04468534114264
C 0.00213309515170 0.59468318280749 -4.80254039628722
C 0.70289226595533 -0.03647831546591 -5.97812113956325

H	1.49982147930980	-0.72073205155344	-5.65135010561775
H	1.15484392934518	0.74559545574500	-6.61480981862890
H	-0.00969771003101	-0.59284383835521	-6.61254050684484
C	-1.46364866076882	1.74550038613326	-6.48781973699508
H	-2.15758098361591	2.59026550420423	-6.59687476016191
H	-1.94651586214679	0.85916972181040	-6.94472425312906
H	-0.57395991781171	1.97308027206380	-7.10076119103933
C	-2.36217886200878	2.33883023801835	-1.62074369820281
C	-2.03223426601859	3.47561304781053	-0.82911973199699
C	-2.80926639170694	3.73161162257714	0.31366502028771
H	-2.57650369822753	4.60779545347528	0.93161302894174
C	-3.86969445718225	2.87955771144739	0.66685920326431
H	-4.46305792051676	3.08999678058085	1.56487224632161
C	-4.16610431178629	1.75139527497174	-0.11728816799871
H	-4.98383045256146	1.08032066588398	0.17482845743112
C	-3.42169801468181	1.45217562256449	-1.27299165366496
C	-0.88012225763902	4.36376989254322	-1.22436554943700
H	-0.78611374897697	5.22053227621937	-0.53764478473010
H	-0.99220689097026	4.74416260981025	-2.25531703649978
H	0.08901380271005	3.81938543573519	-1.19616077715133
C	-3.71671343640568	0.23013943655970	-2.10581908972868
H	-4.39952930821034	-0.44920182975801	-1.57050056100201
H	-2.79411410493524	-0.32533567549936	-2.35421292656539
H	-4.19634489226711	0.49714079342897	-3.06611757449471
C	1.44003794136641	-0.65824536165081	-3.26858737691427
C	1.15637796790494	-2.04854462066593	-3.29174418647504
C	2.17720771432668	-2.92560411649587	-2.87998431014170
H	1.98146438954164	-4.00551362388375	-2.88668326139491
C	3.42448613223049	-2.44210114773868	-2.44646984877033
H	4.20153423336831	-3.14701555290319	-2.12731163965951
C	3.67276845613859	-1.06052323732272	-2.40311615257959
H	4.64078688540412	-0.68262726484675	-2.05114843206646
C	2.68429821963826	-0.14562820348062	-2.80878080510747
C	-0.20397617458774	-2.54467898668405	-3.70836590324975
H	-0.31424671450725	-3.61894678386651	-3.48890139421702
H	-0.37910997254706	-2.40235525995142	-4.79103699905770
H	-1.00380056302437	-1.99255131093382	-3.18113511749803
C	2.91579001154842	1.34555659159851	-2.77218445878991

H	3.90644635802762	1.58891802742486	-2.35500201920954
H	2.18858473035652	1.89288384930812	-2.11216841097710
H	2.82125025385646	1.80952277969649	-3.76955273346749
N	0.28213590163077	-0.03278545376021	-0.38679730677035

Optimized structure of $L^{Me3}FeN_2$(end on) cation

Fe	0.19602800769596	1.21426052150220	-1.49513097125973
N	-1.35093556645360	1.63307965418784	-2.50573062090467
N	0.87897376640628	0.19738908856122	-2.94055055228943
C	-2.76925422361576	2.22636473688389	-4.40837378632029
H	-3.44912878466116	2.56122974102952	-3.61037815005853
H	-3.30118727404700	1.50694504442566	-5.05534712538290
H	-2.51617271946279	3.10045231357053	-5.03583582469221
C	-1.51386767008295	1.61874665184725	-3.83616268740931
C	-0.54300979431248	1.03953067892758	-4.70862385779150
C	0.56844877448816	0.27354541426884	-4.24178696399963
C	1.42102597698300	-0.48630583661057	-5.22557657788505
H	2.18778005621332	-1.08381039503521	-4.70961503814968
H	1.93162187714941	0.21674129472119	-5.90880094734060
H	0.80536945875494	-1.15546707799393	-5.85209010252487
C	-0.77427395282611	1.12735605609818	-6.20124967536884
H	-1.31239175128144	2.04744296813434	-6.47711501692921
H	-1.37421576777856	0.27376517788129	-6.57625183215016
H	0.17458687429824	1.12562528863029	-6.76054640091589
C	-2.33471988097855	2.01515507188605	-1.55236217680289
C	-2.16058917953535	3.25888587411005	-0.87975842037653
C	-3.07541760819468	3.59747544012041	0.13287861667030
H	-2.95933517541864	4.55295889398424	0.65961151167629
C	-4.12684248027706	2.72829780276150	0.46910443423320
H	-4.83066687293071	3.00457194542400	1.26354375482703
C	-4.27635547814681	1.50133918491732	-0.20123111008006
H	-5.09267110767012	0.82176496643445	0.07486479448394
C	-3.38663923210944	1.11552530830752	-1.22002639161862
C	-1.02863650257280	4.17693062102647	-1.26871311219125
H	-1.00700858666616	5.07423683078002	-0.62953162519902
H	-1.10595734147404	4.49252050099578	-2.32484415157833
H	-0.03249786066291	3.68790439482625	-1.17045212343968
C	-3.52073144163511	-0.20639705700589	-1.93093583247160

H	-4.27731167557996	-0.83974370453523	-1.44038010996889
H	-2.55974499486095	-0.75250600371182	-1.94420004375749
H	-3.82400780471403	-0.07340389012616	-2.98623083382196
C	1.84888952744724	-0.67123210883536	-2.36803440304307
C	1.53920543208787	-2.04626672281507	-2.16910348038845
C	2.49568398275300	-2.84087919112679	-1.51174221591876
H	2.27733623299680	-3.90308703011908	-1.34322677137845
C	3.71035929418712	-2.29390034529477	-1.06167699256251
H	4.43846028130585	-2.93293333345593	-0.54754082840829
C	3.99195969891642	-0.93155751273494	-1.25863263468673
H	4.93916141029900	-0.50667683354784	-0.90335478290327
C	3.06937578430273	-0.09540280296323	-1.91179763620593
C	0.22325990338789	-2.61175867091329	-2.63714433333619
H	0.07800515395261	-3.63520141227261	-2.25552591424553
H	0.16581262638521	-2.65108526840917	-3.74099353740328
H	-0.62260312162817	-1.98586704151215	-2.29863644761888
C	3.34787272895619	1.36869784479413	-2.14429907052343
H	4.31224180774582	1.66487902981995	-1.70113161975589
H	2.56994185375375	2.02438234321439	-1.69062287613569
H	3.35925382761048	1.61491442464576	-3.22136201394816
N	0.48408958625871	1.11225661012043	0.35842303919248
N	0.61759760324140	0.99855113118043	1.46245667005824

Optimized structure of $L^{Me3}Fe(THF)N_2$(side on) cation

Fe	-0.18135389461700	0.74490307463092	-1.61057116705661
N	1.03549635304230	2.00519828964516	-0.71538347340672
N	-1.60048273371402	1.72002542455369	-2.46598879600177
N	0.53540838294447	0.28940349505370	-3.33225398635573
C	-3.03793689727657	2.80562697638636	-4.12202945233216
H	-3.69968787899028	2.93098180857623	-3.25102171182548
H	-3.60597400300733	2.30821667402923	-4.92704903497621
H	-2.76218403700810	3.81091105389926	-4.49065650512185
C	-1.80364115123446	2.01313805214532	-3.75529244567375
C	-0.90851179756816	1.58543397876347	-4.78303456699593
C	0.17940284989231	0.68963913977901	-4.55974652195564
C	0.95811886957806	0.14978738797591	-5.73824188594830
H	1.66517365840388	-0.63144062069574	-5.41886130065875

H	1.53702212929706	0.95625130573941	-6.22506785979926
H	0.28483637205816	-0.27505276949161	-6.50274541042143
C	-1.19217189854514	2.02519217587026	-6.20599517953185
H	-1.66812068126490	3.01824139403827	-6.23817787794892
H	-1.86906572201537	1.32426002456145	-6.73611648377149
H	-0.26856622516432	2.09340481707761	-6.80267279779741
C	-2.49577353722831	2.04373417140678	-1.40753996958393
C	-2.34260074804843	3.27645333836698	-0.71293343957288
C	-3.13485092112370	3.49237047382413	0.43197351653988
H	-3.02018401746187	4.43244841931026	0.98696753747048
C	-4.06993416150214	2.53479376499992	0.85799551505158
H	-4.67738983756687	2.72259081275191	1.75176558187250
C	-4.23618811426991	1.34145930165611	0.13469731772298
H	-4.97721390639081	0.59963417923772	0.45919693871830
C	-3.45327598232839	1.06992384960771	-1.00212235426061
C	-1.40471035936672	4.34118101153260	-1.22345619397125
H	-1.04121592991568	4.98059230368007	-0.40174511456750
H	-1.92707599359332	4.99550395299470	-1.94805810573451
H	-0.53905887297521	3.91174337688460	-1.75325782488426
C	-3.60286246552472	-0.21917521297728	-1.77047874704746
H	-4.44567032458959	-0.81307185135300	-1.38117967422175
H	-2.69025299365662	-0.84079928747863	-1.69559107187758
H	-3.77001471598824	-0.03673461452960	-2.84702521699985
C	1.52083048763284	-0.70050348453692	-3.05612822973605
C	1.11149151373252	-2.06300435437439	-2.97215580082697
C	2.05757174175442	-3.01672682418987	-2.55608970698129
H	1.75474153499701	-4.06915309520996	-2.48242174410295
C	3.36900703609337	-2.63545668309306	-2.22343577016154
H	4.08893569544884	-3.39000268639744	-1.88359077320645
C	3.76296785225369	-1.29285443105824	-2.34004159764883
H	4.79492549654435	-1.00126867967186	-2.10530867176331
C	2.85802537405753	-0.30364944223298	-2.77421828242675
C	-0.30475866864813	-2.45503136946549	-3.31143414712435
H	-0.44714500065452	-3.54288004692995	-3.20749800391460
H	-0.57185903908048	-2.16599214375443	-4.34412137176809
H	-1.03487022606700	-1.95048641476112	-2.65077408951467
C	3.31390466203627	1.11578371997546	-3.00372805243796
H	4.09449472570732	1.40778678631589	-2.28102924282868

H 2.48227011084009 1.83546127546855 -2.94299326339220
H 3.74818864571183 1.21453670756117 -4.01745281309347
H 0.33176931416844 -2.38522945301931 -0.30494704326421
H 1.56722630240927 -1.12382981988782 0.03641020704199
H 1.35324162942486 -1.98769344464938 2.29471873311661
C 0.56715973300160 -1.50405110162883 0.31048740273181
C 0.39634596824737 -1.72507344826524 1.81409855683538
H -0.32156883856021 -2.54263878390536 2.00461448084380
O -0.41092868467477 -0.44686863190863 -0.03434310913545
C -0.17507287455206 -0.38357806584056 2.31004443766293
H 0.62481435208665 0.37194087161873 2.41510864944917
C -1.11843374801070 0.00077631174835 1.18094733204282
H -0.70219478810546 -0.47145945038026 3.27455912783176
H -2.08047945889889 -0.53954207366185 1.22344169456767
H -1.31028823755957 1.08110164917886 1.07570509548940
N 1.70383256838353 2.63325658750307 -0.08153086936033

Optimized structure of $L^{Me3}FeNNFeL^{Me3}$

Fe 3.27356319888078 7.90204235503726 6.43237919453857
N 1.62461801955713 8.78365113631058 6.14438694330697
N 4.97387743838193 7.71091876201362 6.84149973500389
C 0.79077792707473 7.43118676834274 4.29116454401853
C -0.40520920334643 7.13809150421071 3.39759106345924
C 1.53150232262625 9.94749593043606 6.96225859057409
C 0.64705254822677 8.45076755388980 5.27174761087617
N 3.07512489122298 6.77910302917083 4.84232060304029
C 2.00111012134227 6.71445476403775 4.03891116834660
C 5.32161874632623 6.72318187986601 3.86013681711697
C 4.27417603508490 6.06299377260466 4.55356370694075
C 2.07891433285693 5.83318396571618 2.80626030096846
H 3.12009962192041 5.54313690411613 2.59781184841014
H 1.67086980209667 6.34594403172233 1.91881546117821
H 1.49318704309103 4.90419538120584 2.94362364926175
C 3.33074877303529 4.10266909610380 5.85529647130459
H 2.43607877716475 3.94754321820738 5.22392724191335
C -0.65759059112837 9.22036593896085 5.33461402597283
H -0.60504306215507 10.04036451175348 6.06628236839977
H -1.48840515072482 8.55404437121934 5.63197680923238

H	-0.92459342489157	9.64603626238362	4.35098126168842
C	4.45038498670179	4.76066712823537	5.08801198574553
C	1.81977469943656	11.21973688241929	6.39426633164962
C	6.54969801401184	6.05340043802383	3.70486279411841
H	7.37009519488185	6.55761792401392	3.18090401341229
C	1.19312239343306	9.81261298027955	8.33519945939303
C	5.69773080892632	4.12830441619839	4.91499396220276
H	5.84686862237637	3.12428521511815	5.33509327018679
C	6.74430582561986	4.76475412141999	4.22711371511900
H	7.71782055787415	4.27398893118819	4.11389455121308
C	5.12721398479075	8.13321945701827	3.36454741516916
H	4.90696095621982	8.81452377415826	4.21125622118309
C	1.10997108743911	10.97645045313883	9.12356573612046
H	0.84074472793474	10.88220239321720	10.18379795708947
C	0.94652182821735	8.44490894523841	8.91908657886098
H	0.14139399308625	7.90894884827257	8.38467269210456
C	1.72889663090841	12.35708172120210	7.21923268473118
H	1.95250710771336	13.34318805115547	6.79105538677848
C	1.36755232219381	12.24321248999692	8.57335123904450
H	1.30188354871931	13.13954264664081	9.20222034230354
H	4.26911303937056	8.21579574388087	2.67241168935385
C	2.22458633551998	11.32368731672857	4.94594001023166
H	1.39523093088085	11.05134282278692	4.26659130417538
H	3.64377009933353	3.12352612345768	6.25515715583627
H	0.67995927833698	8.50911446179316	9.98714739994564
H	1.85290922172379	7.80853034622382	8.83406214731160
H	3.00739191523894	4.74292082413112	6.69905721026720
H	6.03075941601149	8.50115438666779	2.85032207714257
H	3.05142771500975	10.62297109130323	4.72295239784995
H	2.54843672498345	12.34793338479210	4.69720799259320
Fe	7.88069062496615	7.94954163905062	6.92440159174381
N	9.67705935761817	7.97396332250352	6.33553069721696
N	6.14373354668985	7.67765403521563	6.89221252766812
C	10.40150260428840	10.06729937928917	7.34789853737471
C	11.53560929521799	11.06893445286074	7.50377604737459
C	9.92493927619000	6.81554240692236	5.54234991470398
C	10.64410163727425	8.87793572438977	6.60689459402057
N	8.09036387170003	9.58075719926680	7.96588243013944

C	9.17223793358551	10.37471116631525	8.01041469886741
C	6.78544662554143	9.30804946567403	10.02163891510627
C	6.91101899063827	9.85608469420431	8.71738432824758
C	9.09964660895450	11.64474129451913	8.83504587434543
H	8.14005212603021	11.71628307834572	9.36900865818513
H	9.91803632790220	11.68840084525649	9.57585344823846
H	9.20262956707050	12.53900151602297	8.19270498240918
C	5.97935218596785	11.08677295242305	6.70244186273846
H	6.81517314955400	11.80570167319066	6.61983821540906
C	12.04633293399626	8.58501119363807	6.10715242840839
H	12.33275871248993	9.26033707244039	5.27896375645522
H	12.79105642217387	8.72138372516797	6.90967076738613
H	12.12384229886215	7.55133344584180	5.73680950281921
C	5.83554838997986	10.54778246282944	8.10251522286832
C	10.11207901074481	5.56376961757327	6.19024013469686
C	5.55859946019328	9.45737994679218	10.69768494917262
H	5.45142470445280	9.02984259198806	11.70391096672916
C	9.93189885561321	6.92141921002492	4.12473529569049
C	4.62570490303834	10.67259764358568	8.81093655751366
H	3.78474636281447	11.19232383576485	8.33900919154505
C	4.48135985690231	10.13267065109616	10.09915056044709
H	3.52601741745580	10.23539852975401	10.62785178736653
C	7.94275967459012	8.56191537082766	10.63748623125862
H	8.28145885367008	7.74544199608747	9.97015718633454
C	10.16373651533470	5.75694404078802	3.36873132415395
H	10.16952360772882	5.82752883209000	2.27292747375951
C	9.66825537886076	8.25187481625585	3.46686453240586
H	10.48229215660824	8.97607261625150	3.65577718595381
C	10.33695101232074	4.42342903034418	5.39590774249348
H	10.48685445408587	3.45313765239062	5.88751965003545
C	10.37309608487055	4.51564974616379	3.99404995421075
H	10.55218818378422	3.61857942131558	3.38851691919338
H	8.81958055549768	9.21936718500279	10.78899662467173
C	10.06720989878324	5.48285219066672	7.69604576394622
H	10.83592418557768	6.12562694654272	8.16262367869187
H	5.05208270827390	11.58736939130064	6.37774904286625
H	9.55833598819092	8.13994330738757	2.37520769622879
H	8.74467306519978	8.70592977512417	3.87390829298787

H	6.19992736459791	10.26809725793255	5.98966699070669
H	7.66436750243496	8.12906268781437	11.61300313570131
H	9.09114498531962	5.83788197558160	8.09101726791613
H	10.21464686217583	4.44717867038290	8.04472120649112
H	11.18146802238838	12.10595332045614	7.36665494449240
H	12.00902830968073	11.02743094845892	8.50632771933110
H	12.33345356042894	10.91119536125646	6.76330325700663
H	-0.38705361266896	6.10572571626060	3.01378124460269
H	-0.46000323887430	7.81016418861658	2.51591749406785
H	-1.35700733830670	7.25204178873940	3.94203248891556

Optimized structure of $L^{Me3}FeNNFeL^{Me3}(THF)_2$

Fe	-9.906860	3.099679	1.055466
N	-10.526847	1.572691	0.370851
N	-8.173481	3.885844	0.826170
N	-9.894317	3.376590	2.974555
C	-5.889013	4.621146	1.354239
H	-5.870562	4.833041	0.273782
H	-5.116910	3.855498	1.558998
H	-5.585394	5.536344	1.893186
C	-7.261154	4.137808	1.787465
C	-7.531534	3.968597	3.175279
C	-8.825720	3.704899	3.723038
C	-9.003370	3.804817	5.227351
H	-10.069038	3.778954	5.502541
H	-8.560653	4.732822	5.629729
H	-8.504657	2.961553	5.742700
C	-6.387568	4.182190	4.156321
H	-6.327175	5.224459	4.534786
H	-5.410460	3.953151	3.701414
H	-6.480223	3.529994	5.041481
C	-8.021508	4.343415	-0.508337
C	-8.284913	5.713146	-0.798927
C	-8.230040	6.143836	-2.137453
H	-8.445696	7.196125	-2.366801
C	-7.917365	5.247475	-3.173500
H	-7.880048	5.595947	-4.213174
C	-7.643412	3.902928	-2.870066

H	-7.374961	3.204719	-3.672687
C	-7.689361	3.427843	-1.545162
C	-8.652142	6.659647	0.316166
H	-8.942024	7.646862	-0.080877
H	-7.815757	6.805616	1.024742
H	-9.491848	6.254273	0.910596
C	-7.387854	1.988175	-1.218678
H	-7.073106	1.433419	-2.118193
H	-8.278945	1.490002	-0.795486
H	-6.594568	1.907557	-0.453271
C	-11.183410	3.205210	3.539790
C	-11.619597	1.905045	3.916415
C	-12.930105	1.754378	4.408693
H	-13.268174	0.756751	4.715317
C	-13.797395	2.854312	4.525367
H	-14.816109	2.714717	4.908169
C	-13.348121	4.134225	4.157367
H	-14.017774	5.000044	4.250660
C	-12.042833	4.332895	3.669188
C	-10.684523	0.730459	3.782968
H	-11.178080	-0.209708	4.078137
H	-9.776498	0.861918	4.400226
H	-10.339048	0.632657	2.737211
C	-11.555685	5.700176	3.260111
H	-10.640619	5.991330	3.807907
H	-12.327912	6.466707	3.441231
H	-11.294849	5.707861	2.184997
H	-12.723240	3.065448	1.268865
H	-13.464703	4.634614	0.805246
H	-14.390090	3.140187	-0.924759
C	-12.818843	3.804592	0.457494
C	-13.291965	3.231874	-0.873218
H	-12.849011	2.232197	-1.035676
O	-11.486248	4.335878	0.184450
C	-12.719398	4.253644	-1.871324
H	-13.374670	5.142160	-1.928262
C	-11.367006	4.619864	-1.247914
H	-12.604002	3.848042	-2.890252

H	-10.539908	4.002193	-1.641805
H	-11.096715	5.681861	-1.368907
H	-7.708738	-1.286824	2.166302
C	-9.405721	-1.994405	0.965793
C	-8.193171	-1.099486	1.193081
H	-8.492818	-0.036238	1.150106
C	-7.306564	-1.468448	-0.009089
C	-8.327507	-1.666624	-1.135263
H	-6.565658	-0.690672	-0.258973
H	-8.494434	-0.742940	-1.718132
Fe	-11.338670	-0.854124	-1.110001
N	-10.889259	0.596486	-0.172777
N	-11.659824	-1.005990	-2.993599
N	-12.932076	-1.857094	-0.648131
C	-12.885841	-1.273450	-5.106389
H	-11.903110	-1.017575	-5.532292
H	-13.228685	-2.218021	-5.565403
H	-13.600495	-0.487591	-5.416051
C	-12.810899	-1.373439	-3.593694
C	-13.945250	-1.843082	-2.872319
C	-13.944120	-2.165747	-1.479130
C	-15.145718	-2.898463	-0.910056
H	-14.943396	-3.258019	0.110582
H	-15.430392	-3.763049	-1.535132
H	-16.029068	-2.233105	-0.861189
C	-15.220496	-2.122827	-3.654743
H	-15.288747	-3.170630	-4.016299
H	-15.306087	-1.475512	-4.542568
H	-16.122707	-1.939360	-3.046835
C	-10.445451	-0.844016	-3.709593
C	-9.722384	-2.005320	-4.107716
C	-8.471085	-1.843061	-4.730789
H	-7.904890	-2.736431	-5.027089
C	-7.938699	-0.563491	-4.962488
H	-6.960487	-0.451318	-5.446491
C	-8.669951	0.573314	-4.578009
H	-8.269273	1.575031	-4.776947
C	-9.925455	0.458013	-3.950645

C	-10.284200	-3.374917	-3.819950
H	-9.550892	-4.162690	-4.060844
H	-11.204997	-3.574433	-4.399232
H	-10.565204	-3.463470	-2.754223
C	-10.712050	1.677249	-3.544251
H	-10.826964	1.717405	-2.445842
H	-10.212310	2.602338	-3.876007
H	-11.734617	1.653515	-3.962963
C	-12.917082	-2.279543	0.705541
C	-13.399767	-1.409298	1.721491
C	-13.310887	-1.829975	3.062337
H	-13.696334	-1.170131	3.849489
C	-12.755540	-3.075665	3.402022
H	-12.693080	-3.383322	4.453316
C	-12.290734	-3.927868	2.385538
H	-11.860274	-4.905580	2.641578
C	-12.367795	-3.552162	1.031125
C	-13.991626	-0.073391	1.350970
H	-14.294687	0.491344	2.247548
H	-14.870667	-0.187246	0.689864
H	-13.255094	0.531131	0.790290
C	-11.856210	-4.452975	-0.064522
H	-12.637334	-4.674376	-0.814895
H	-11.488974	-5.408344	0.346815
H	-11.027860	-3.960048	-0.607416
H	-10.340015	-1.626381	1.419306
H	-9.226225	-3.031842	1.309841
O	-9.592992	-2.011742	-0.482680
H	-6.761511	-2.409051	0.192004
H	-8.066428	-2.477749	-1.834633

Optimized structure of $K_2L^{Me3}FeNNFeL^{Me3}$

Fe	0.169716146	-0.119722566	2.359744336
Fe	0.280168146	-0.094865566	-2.351842664
K	0.941059000	2.489175000	0.030453000
K	-0.474317003	-2.581052342	-0.065736514
N	0.223490146	-0.115972566	0.618481336
N	0.247729146	-0.107856566	-0.610134664

N	0.078329146	1.306202434	3.660714336
N	0.173632146	-1.546282566	3.662191336
N	1.162941146	1.052466434	-3.632740664
N	-0.552738854	-1.229839566	-3.676174664
C	-0.160677854	2.320063434	6.049884336
C	0.017937146	1.162812434	5.004461336
C	0.058176146	-0.121903566	5.584294336
C	0.011264152	-0.122367534	7.133584189
C	0.143252146	-1.405548566	5.007024336
C	0.281874146	-2.562247566	6.059093336
H	-1.132074787	2.159611368	6.553748538
H	-0.175046030	3.360261579	5.674361535
H	0.657892035	2.272020423	6.792091319
H	0.300926838	-3.603234262	5.685971782
H	-0.557264937	-2.511407287	6.777770500
H	1.238331456	-2.402053272	6.590848169
C	0.268115146	2.542857434	3.006429336
C	1.580171146	3.115115434	2.938392336
C	1.749054146	4.341858434	2.269009336
H	2.747507146	4.792303434	2.216209336
C	0.663684146	5.004290434	1.665347336
H	0.815871146	5.960705434	1.152040336
C	-0.618357854	4.427698434	1.731727336
H	-1.467565854	4.948629434	1.271045336
C	-0.842130854	3.212721434	2.405945336
C	2.770016146	2.369014434	3.533206336
H	2.377321146	1.726194434	4.341405336
H	3.437971675	3.081353027	3.975551482
H	3.096206066	1.635551058	2.827236296
C	-2.240523854	2.618255434	2.502192336
H	-2.177399854	1.790825434	3.232860336
H	-2.873435256	3.358361668	2.942080518
H	-2.651502301	2.144992573	1.586071356
C	0.044868146	-2.782474566	2.993355336
C	1.198640146	-3.423790566	2.447608336
C	1.038440146	-4.641177566	1.759707336
H	1.923252146	-5.138255566	1.341099336
C	-0.224823854	-5.245581566	1.622775336

H	-0.328421854	-6.202926566	1.099129336
C	-1.355462854	-4.607735566	2.167154336
H	-2.340159854	-5.077792566	2.057561336
C	-1.249109854	-3.381939566	2.850303336
C	2.576931146	-2.798184566	2.607848336
H	2.443998146	-1.913271566	3.257199336
H	2.760854006	-2.606341113	1.572605497
H	3.509090950	-3.206761784	2.930320839
C	-2.483285854	-2.662276566	3.383932336
H	-2.148351854	-2.026872566	4.223440336
H	-2.768090111	-2.034910938	2.523580166
H	-2.944039041	-3.489036921	3.858436959
C	1.920704146	1.803538434	-6.012085664
C	1.118149146	0.934718434	-4.979553664
C	0.339275146	-0.077346566	-5.577758664
C	0.357161146	-0.070004566	-6.666855664
C	-0.457664854	-1.097790566	-5.019206664
C	-1.224493854	-1.953616566	-6.089056664
H	2.465941293	2.544790565	-5.483530948
H	1.525635442	2.488340881	-6.725154647
H	2.593733611	1.147721461	-6.527661010
H	-1.908097076	-1.287497813	-6.535096060
H	-0.499871400	-2.415649991	-6.788108287
H	-1.685594024	-2.891527190	-5.885706991
C	1.756111146	2.140771434	-2.955957664
C	3.058261146	2.004321434	-2.384959664
C	3.616366146	3.096091434	-1.693726664
H	4.617411146	2.995227434	-1.254875664
C	2.929215146	4.318153434	-1.575757664
H	3.385217146	5.163182434	-1.047198664
C	1.647000146	4.443380434	-2.142286664
H	1.108632146	5.393753434	-2.044999664
C	1.041422146	3.376037434	-2.831699664
C	3.821693146	0.693510434	-2.511510664
H	3.241802146	0.059502434	-3.207545664
H	3.849472146	-0.030248566	-1.154983664
H	4.811939783	0.890743713	-2.959657595
C	-0.372552854	3.498831434	-3.388689664

H	-0.464678854	2.757710434	-4.202798664
H	-0.529674642	4.454211743	-3.827716104
H	-1.230495103	3.316564916	-2.659977018
C	-1.157504854	-2.332478566	-3.033803664
C	-0.434161854	-3.562240566	-2.901924664
C	-1.051321854	-4.644867566	-2.247603664
H	-0.507421854	-5.591593566	-2.145775664
C	-2.352779854	-4.539637566	-1.722064664
H	-2.817651854	-5.396286566	-1.220690664
C	-3.048730854	-3.323318566	-1.847729664
H	-4.065530854	-3.239307566	-1.442900664
C	-2.480276854	-2.217110566	-2.506876664
C	0.998784146	-3.661629566	-3.413786664
H	1.104131146	-2.918718566	-4.224780664
H	1.681330453	-3.322226771	-2.635897244
H	1.135180099	-4.662461247	-3.771251908
C	-3.256126854	-0.915733566	-2.651487664
H	-2.667278854	-0.274729566	-3.333315664
H	-4.271828311	-1.222444383	-2.982207245
H	-3.237771138	-0.317166137	-1.726334969
H	-1.061804588	-0.110780790	7.390135214
H	0.472177746	0.630367252	7.800056244
H	0.592963971	-0.981338789	7.517028715
H	-0.323484602	-0.497742072	-7.426459503
H	1.215423217	-0.588224663	-7.133835480
H	0.741752461	0.841729166	-7.160887162
