# Supplementary material for: Mössbauer and Nuclear Resonance Vibrational Spectroscopy Studies of Iron Species Involved in N–N Bond Cleavage
Source: Inorg Chem. 2023 Oct 30;62(45):18449–64. doi: 10.1021/acs.inorgchem.3c02594 (PMC10647920; doi:10.1021/acs.inorgchem.3c02594)
Supplement: Supplementary file 2 — ic3c02594_si_002.zip [file ic3c02594_si_002.zip › animations/animations.pptx]

## Slide 1
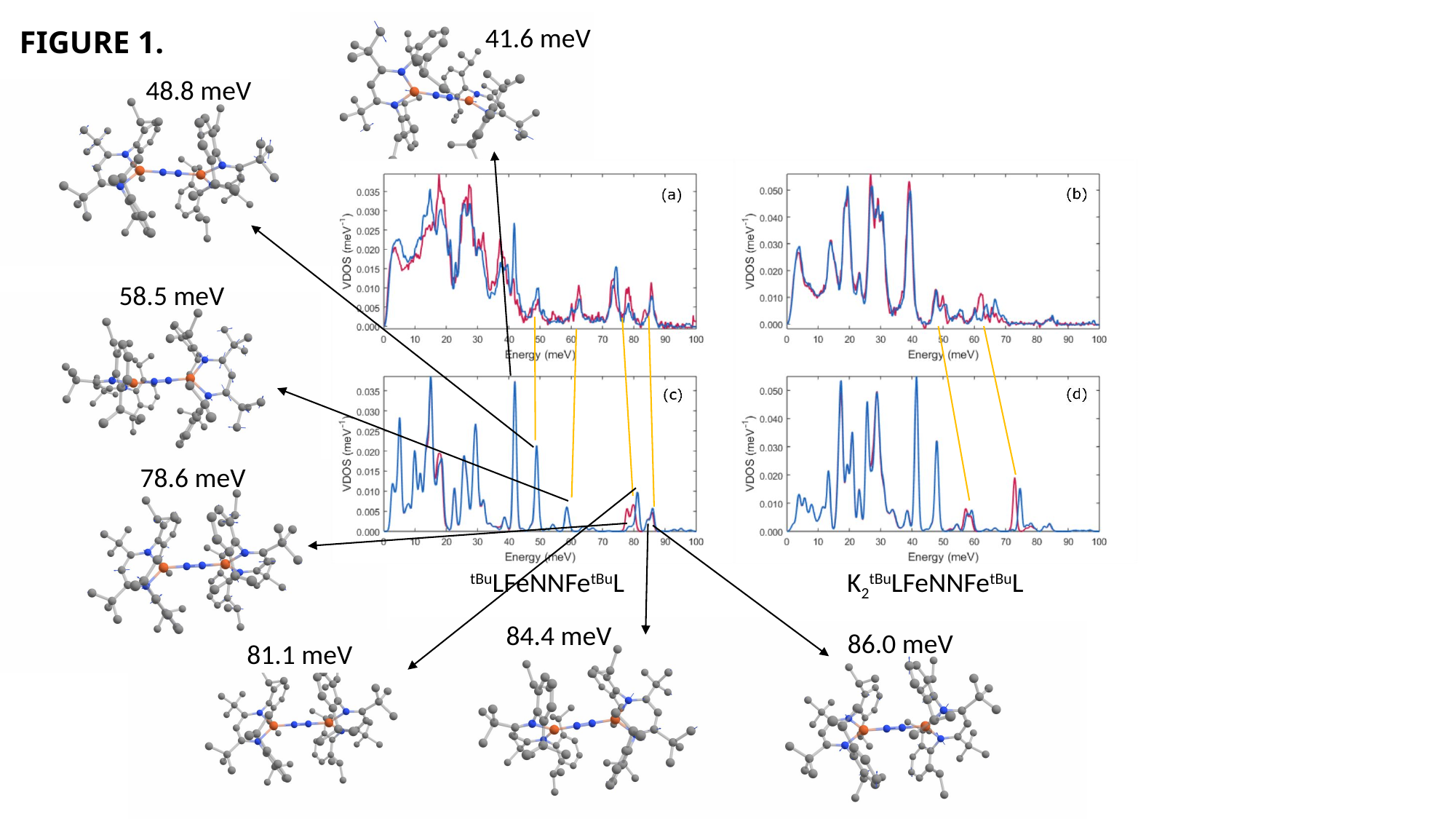

# FIGURE 1.
41.6 meV
48.8 meV
58.5 meV
78.6 meV
tBuLFeNNFetBuL 		 K2tBuLFeNNFetBuL
84.4 meV
86.0 meV
81.1 meV

## Slide 2
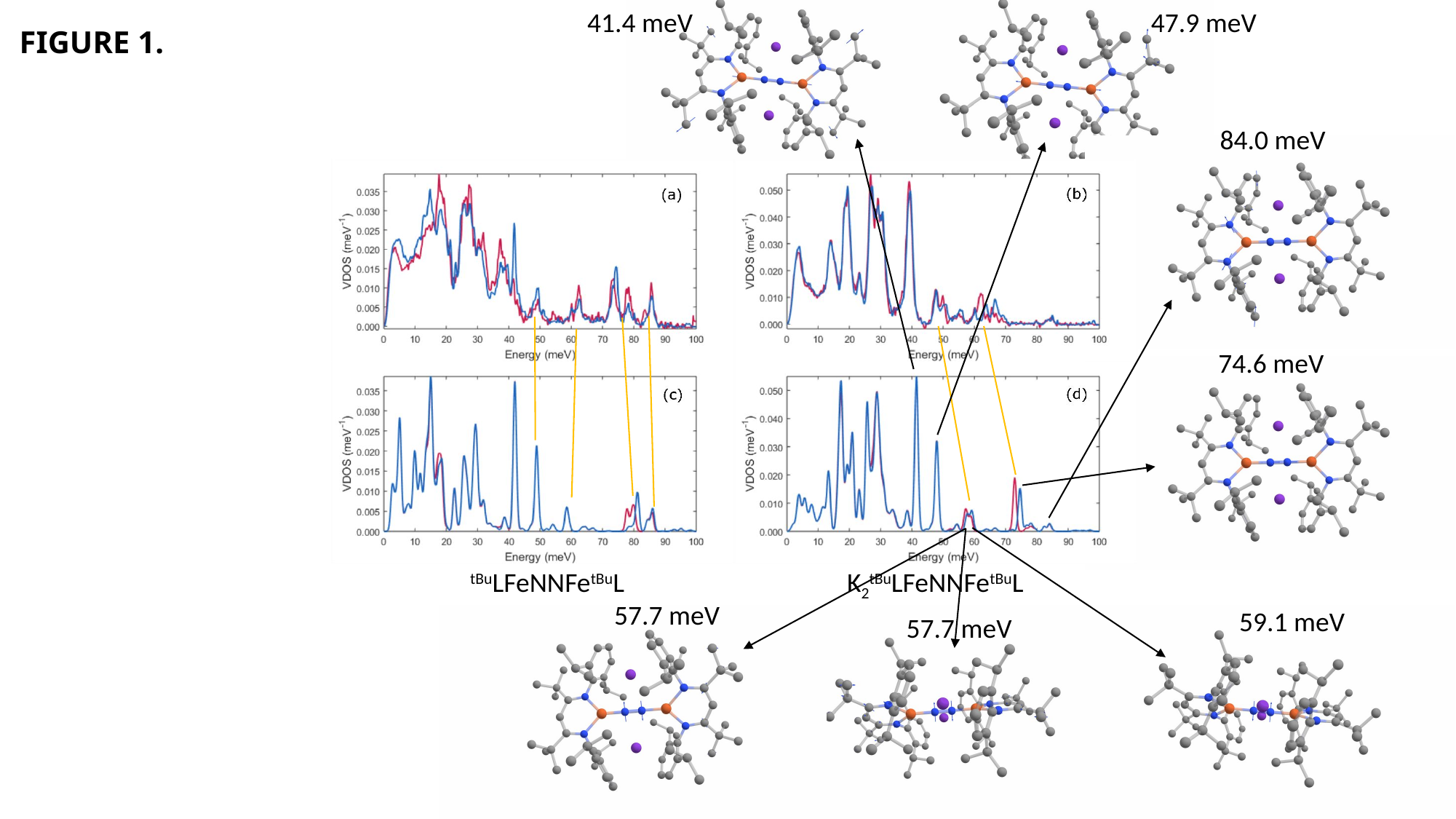

41.4 meV
47.9 meV
# FIGURE 1.
84.0 meV
74.6 meV
tBuLFeNNFetBuL 		 K2tBuLFeNNFetBuL
57.7 meV
59.1 meV
57.7 meV

## Slide 3
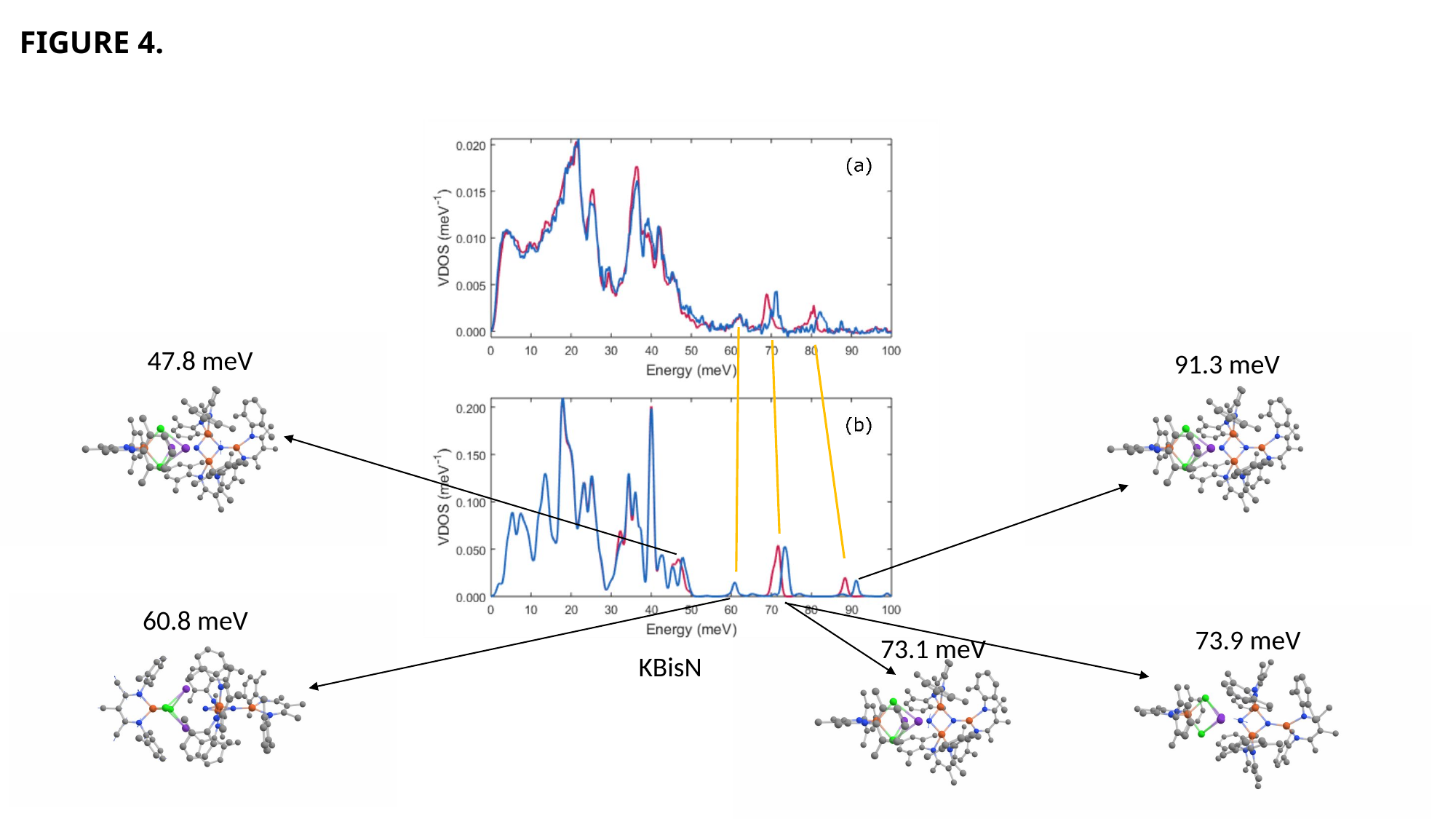

# FIGURE 4.
47.8 meV
91.3 meV
60.8 meV
73.9 meV
73.1 meV
KBisN
