# Supplementary figures and images for: Mössbauer and Nuclear Resonance Vibrational Spectroscopy Studies of Iron Species Involved in N–N Bond Cleavage
Source: Inorg Chem. 2023 Oct 30;62(45):18449–64. doi: 10.1021/acs.inorgchem.3c02594 (PMC10647920; doi:10.1021/acs.inorgchem.3c02594)

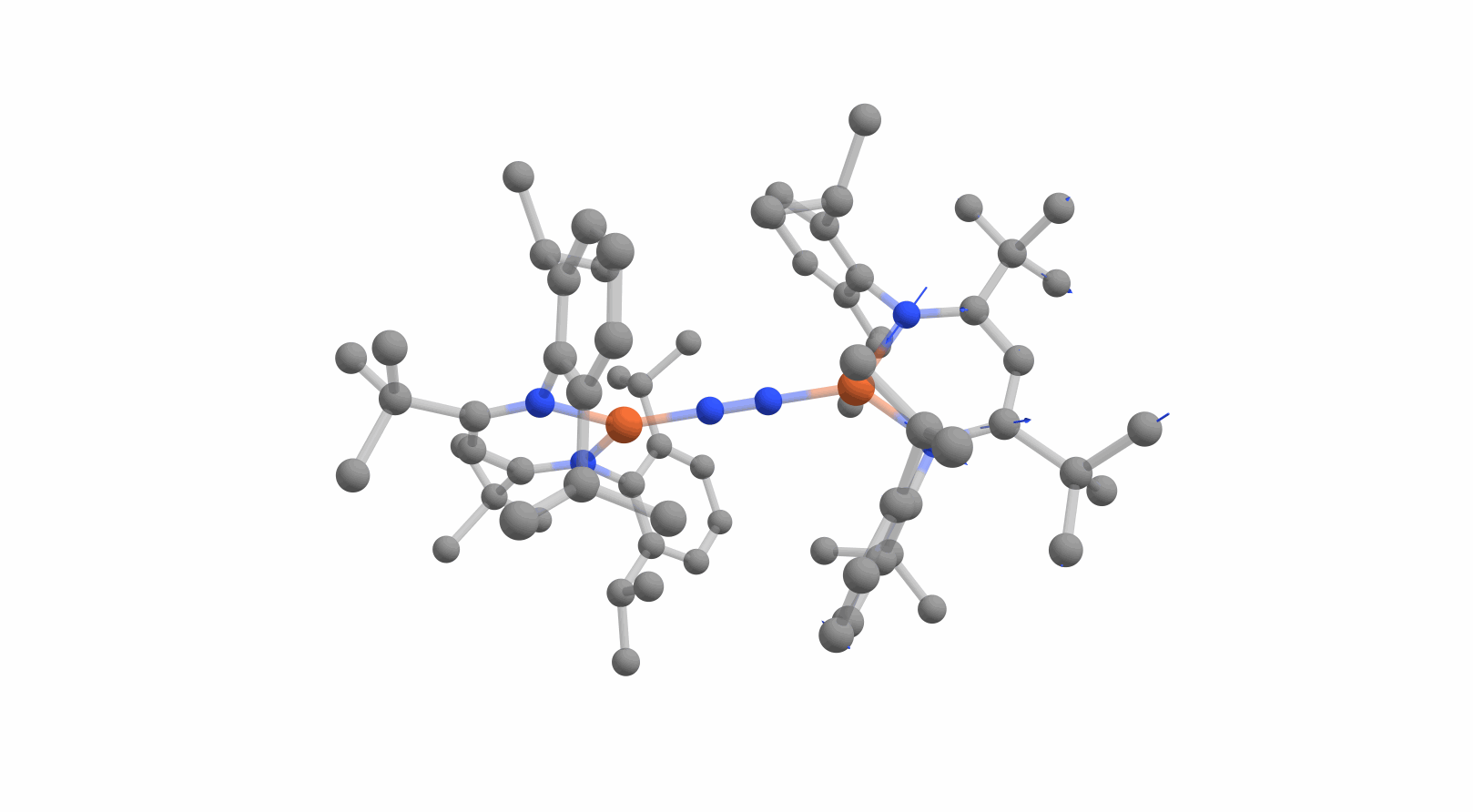

Supplement: Supplementary file 2 — ic3c02594_si_002.zip [file ic3c02594_si_002.zip › animations/tBuFeNNFeL_84_4a.gif]

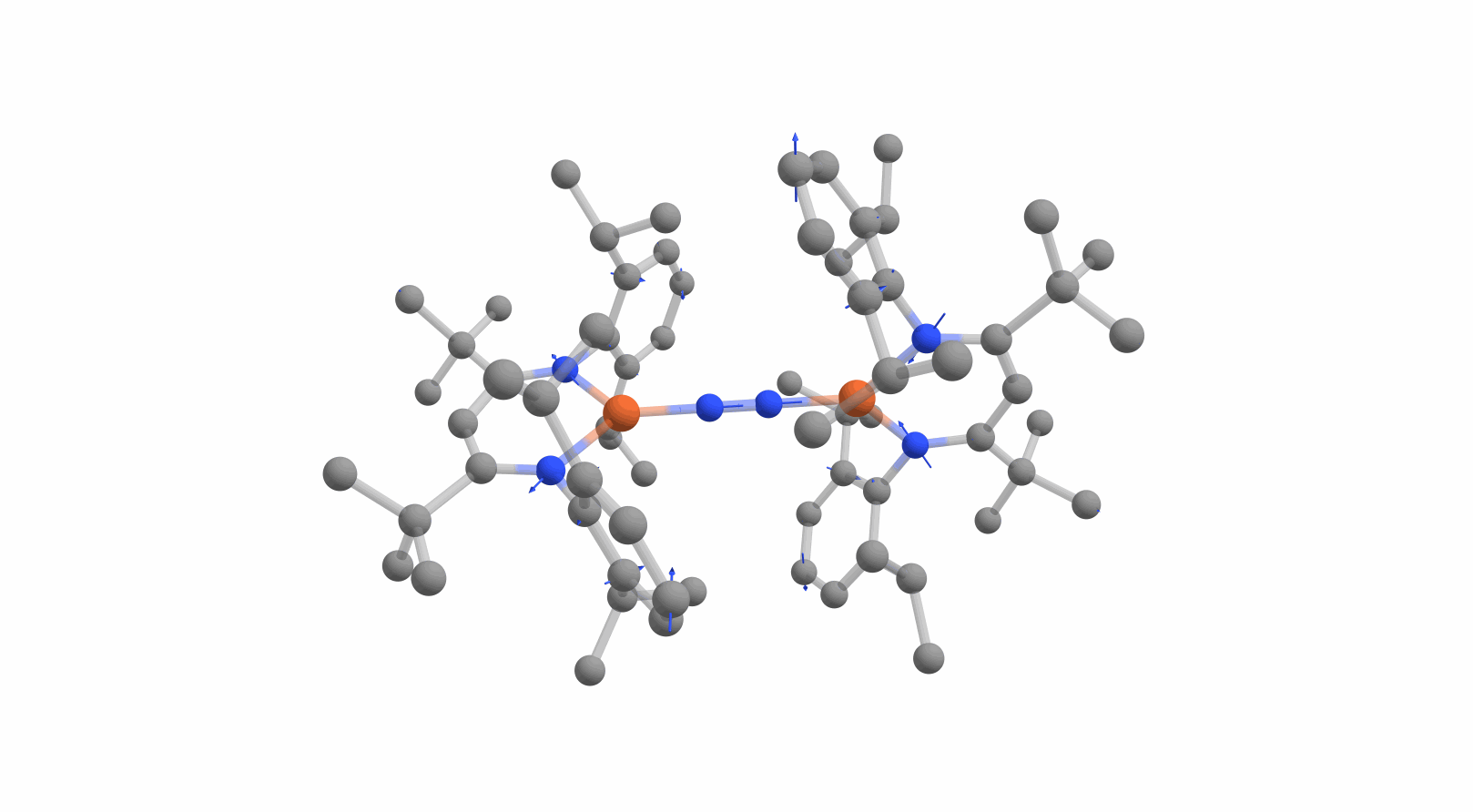

Supplement: Supplementary file 2 — ic3c02594_si_002.zip [file ic3c02594_si_002.zip › animations/tBuFeNNFeL_85_8.gif]

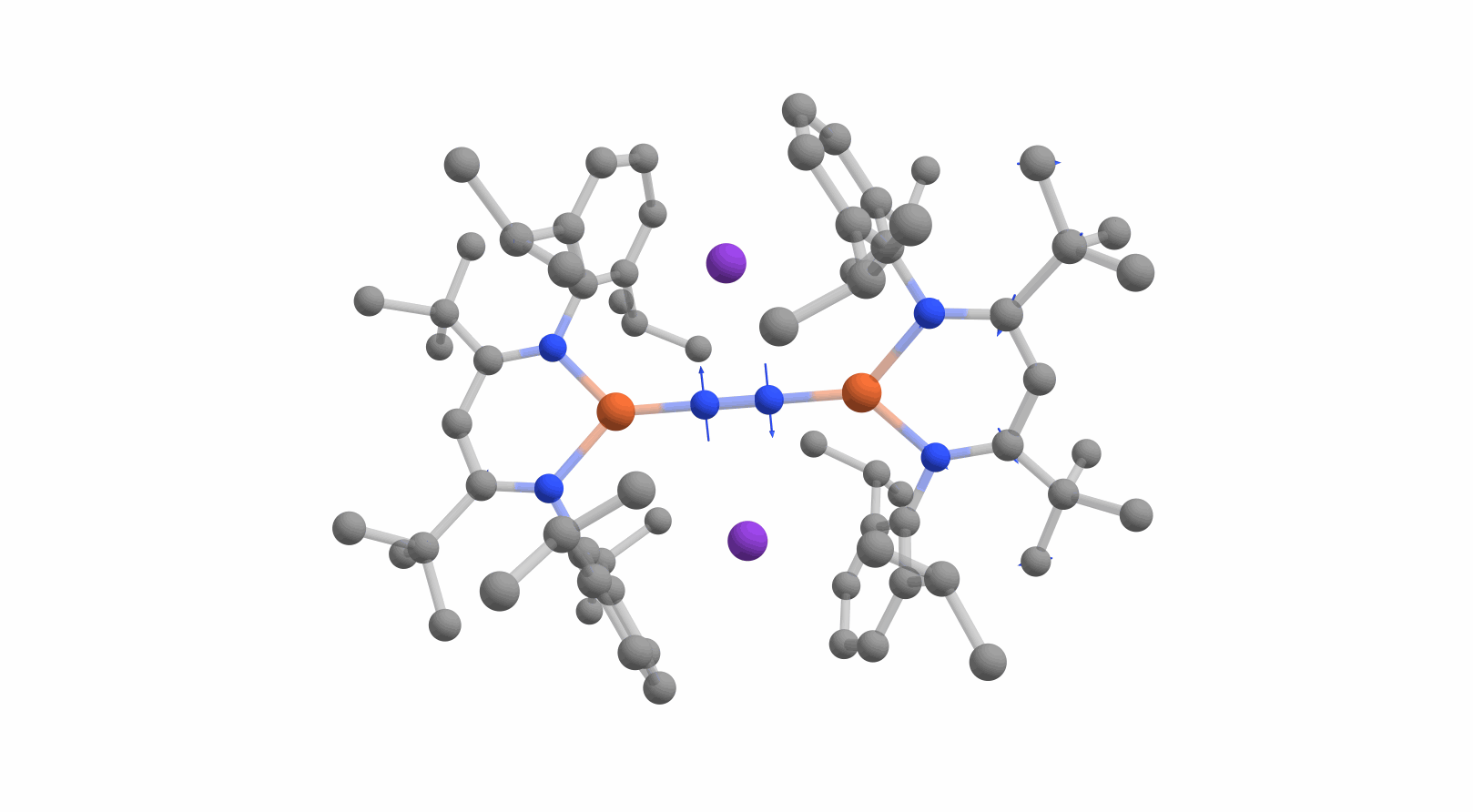

Supplement: Supplementary file 2 — ic3c02594_si_002.zip [file ic3c02594_si_002.zip › animations/K2tBuFeNNFeL_57_2a.gif]

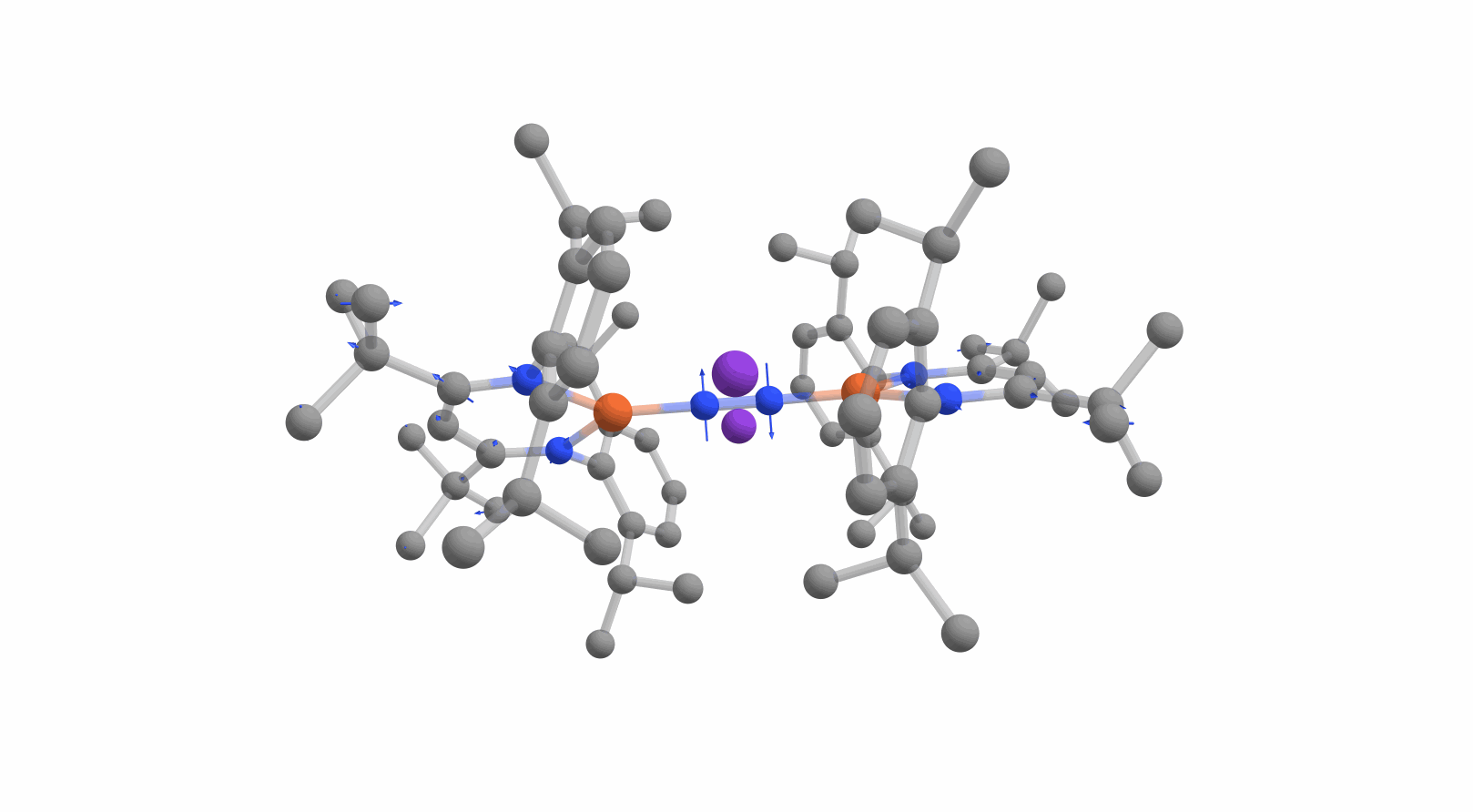

Supplement: Supplementary file 2 — ic3c02594_si_002.zip [file ic3c02594_si_002.zip › animations/K2tBuFeNNFeL_57_2b.gif]

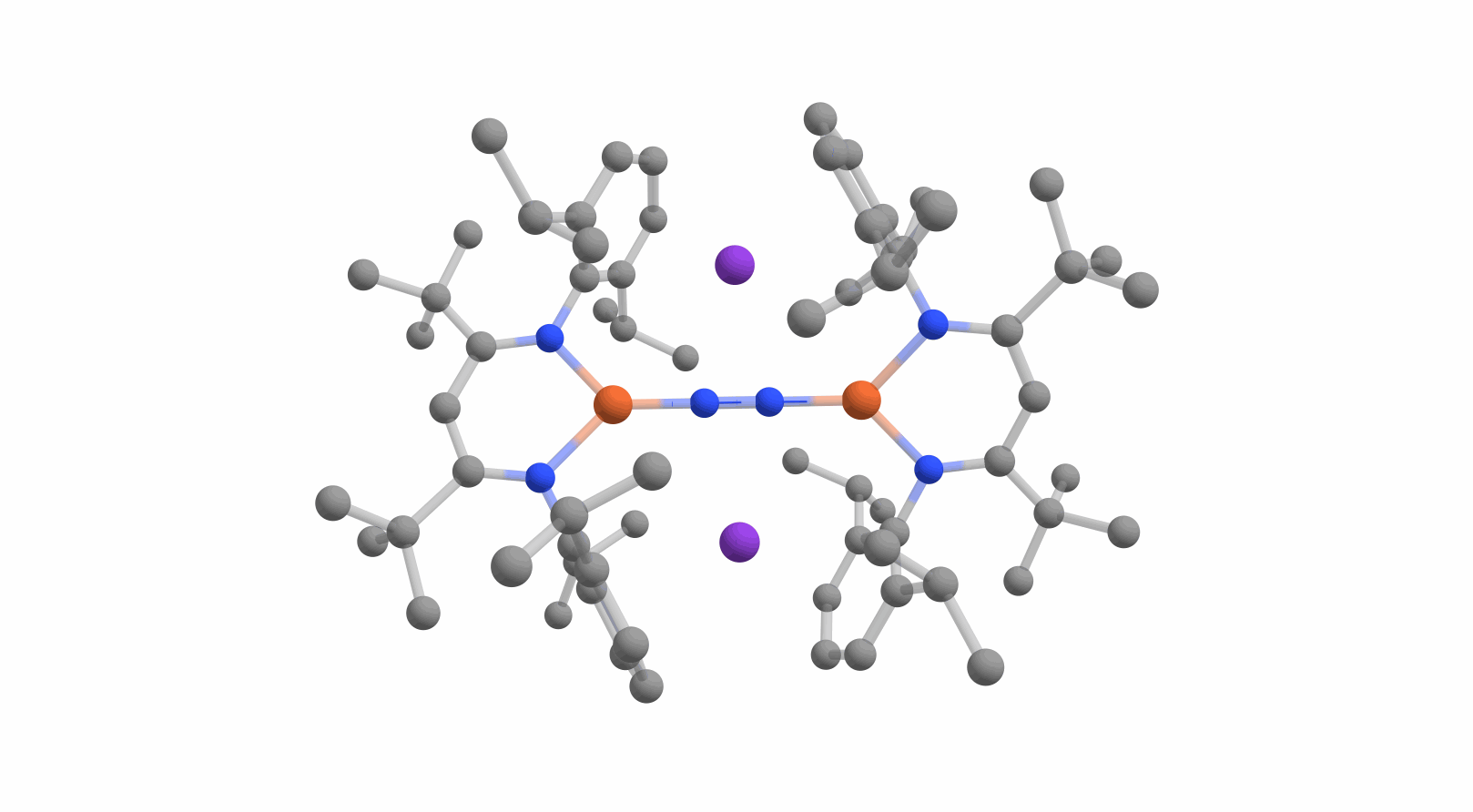

Supplement: Supplementary file 2 — ic3c02594_si_002.zip [file ic3c02594_si_002.zip › animations/K2tBuFeNNFeL_74_6.gif]

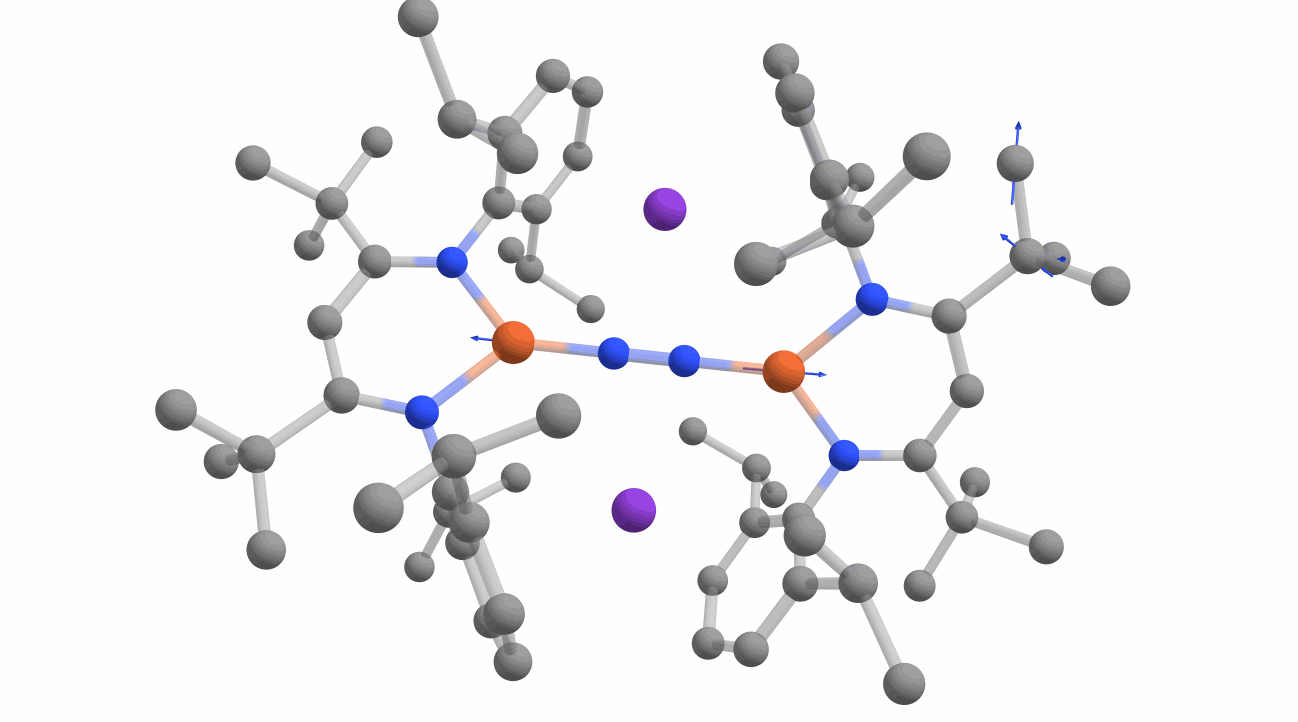

Supplement: Supplementary file 2 — ic3c02594_si_002.zip [file ic3c02594_si_002.zip › animations/K2tBuFeNNFeL_47_9.gif]

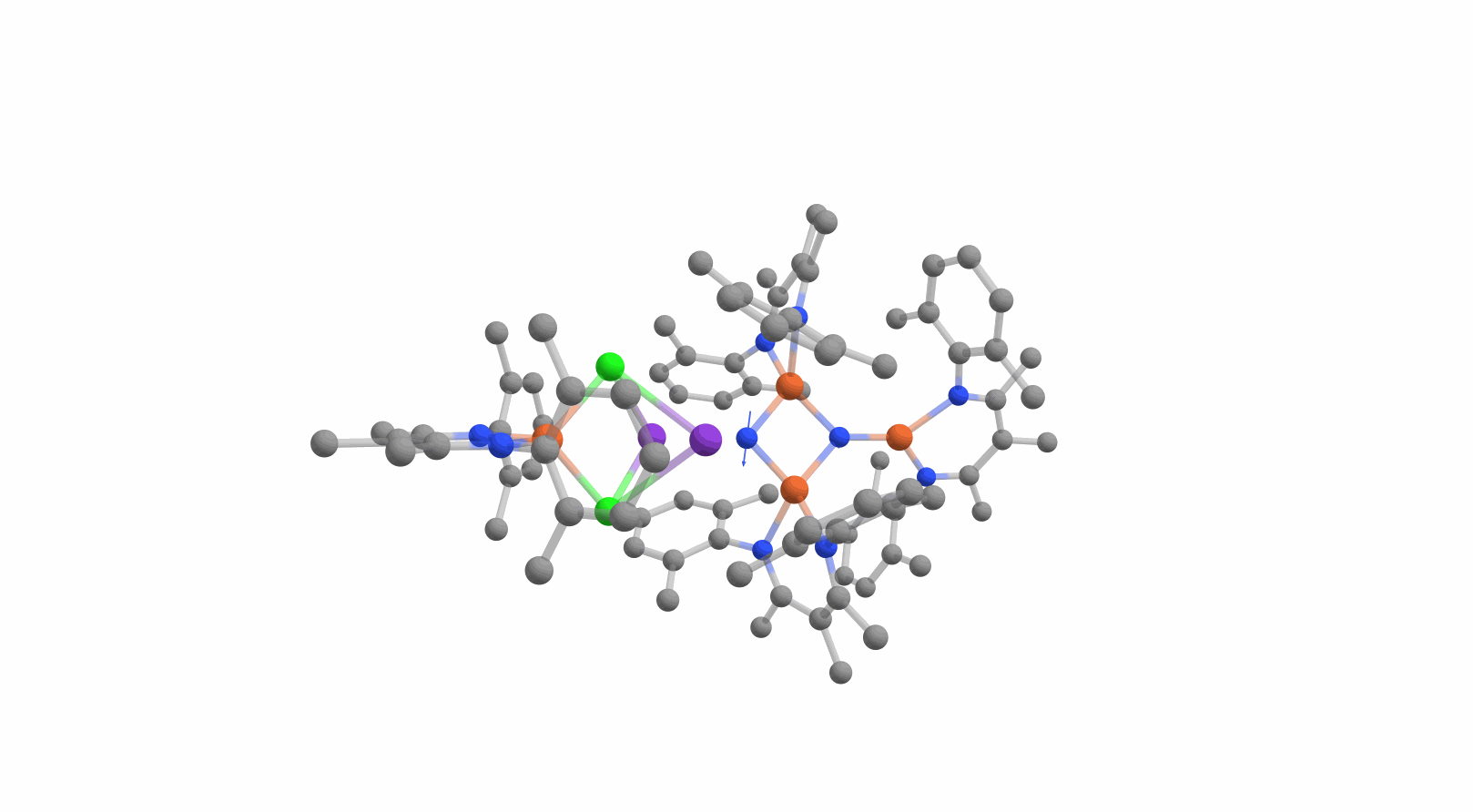

Supplement: Supplementary file 2 — ic3c02594_si_002.zip [file ic3c02594_si_002.zip › animations/KBisN_73_4a.gif]

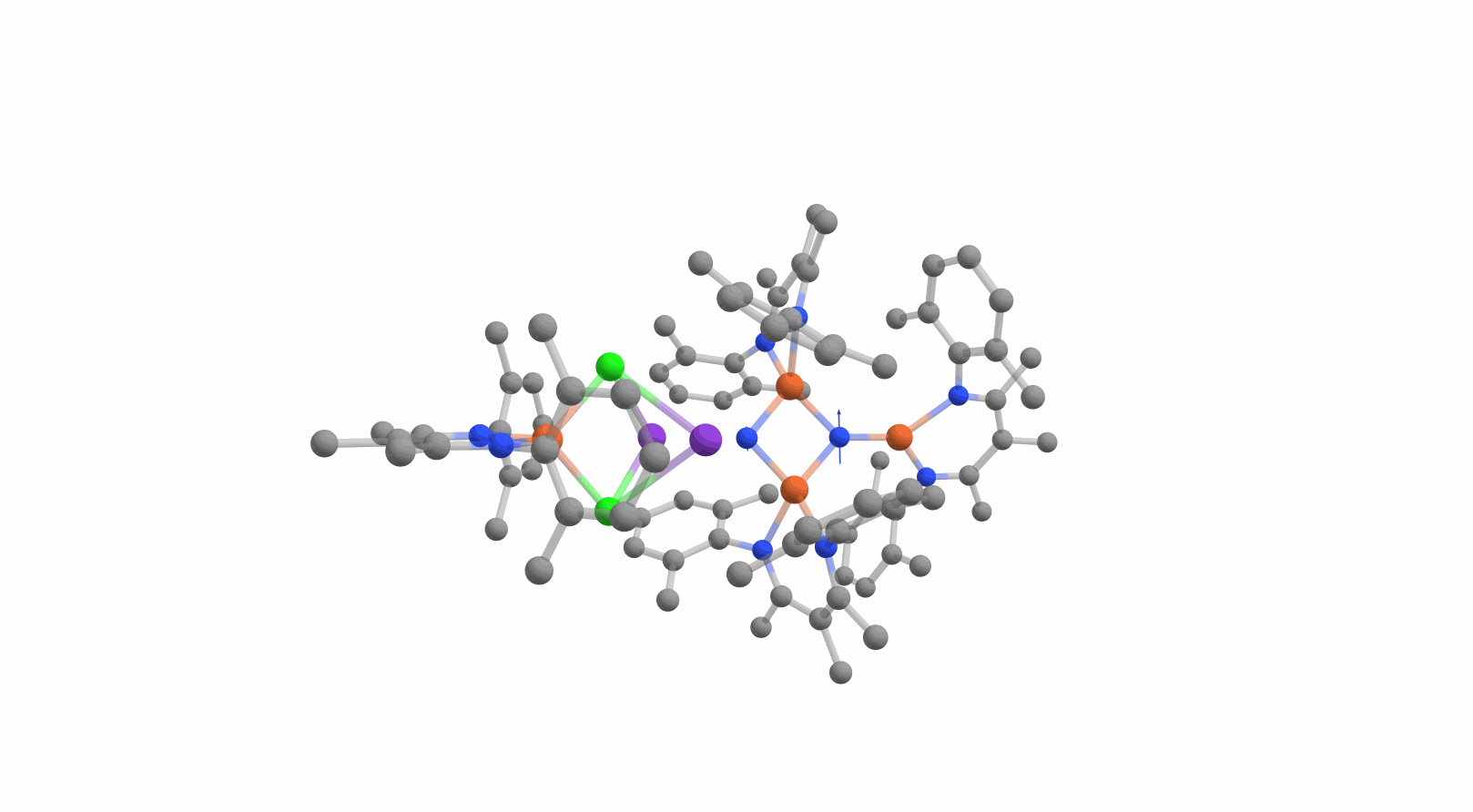

Supplement: Supplementary file 2 — ic3c02594_si_002.zip [file ic3c02594_si_002.zip › animations/KBisN_47_8.gif]

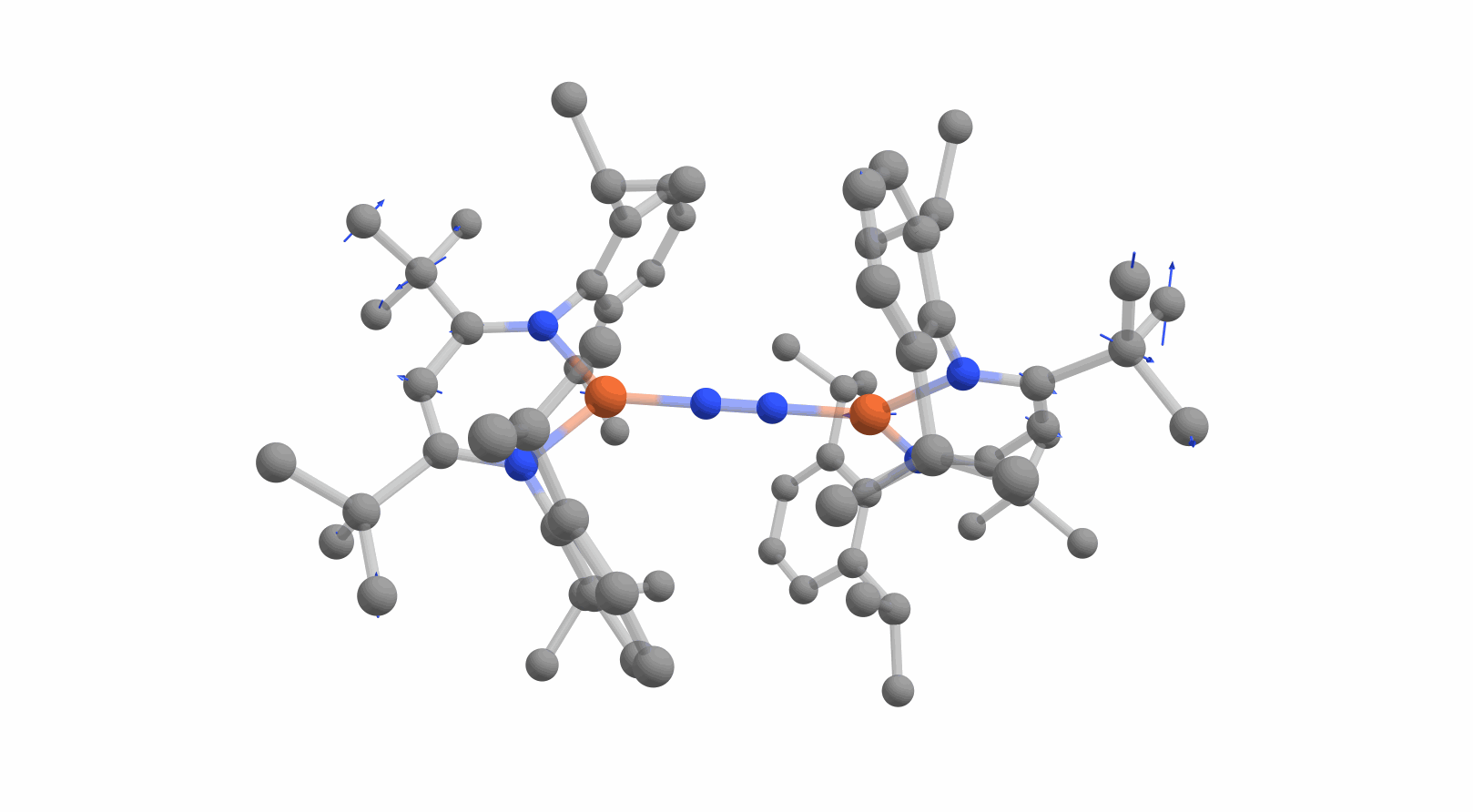

Supplement: Supplementary file 2 — ic3c02594_si_002.zip [file ic3c02594_si_002.zip › animations/tBuFeNNFeL_48_8.gif]

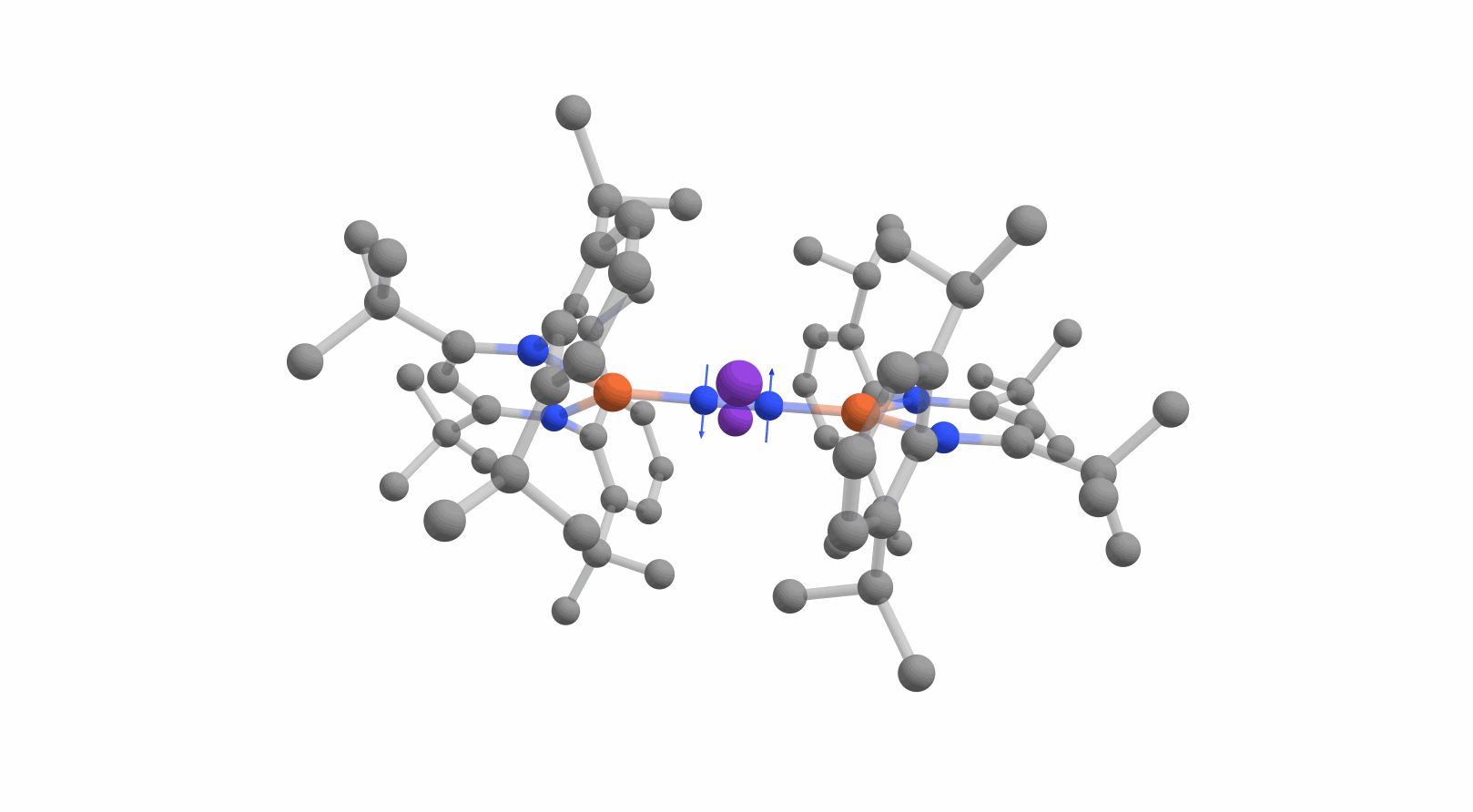

Supplement: Supplementary file 2 — ic3c02594_si_002.zip [file ic3c02594_si_002.zip › animations/K2tBuFeNNFeL_58_7.gif]

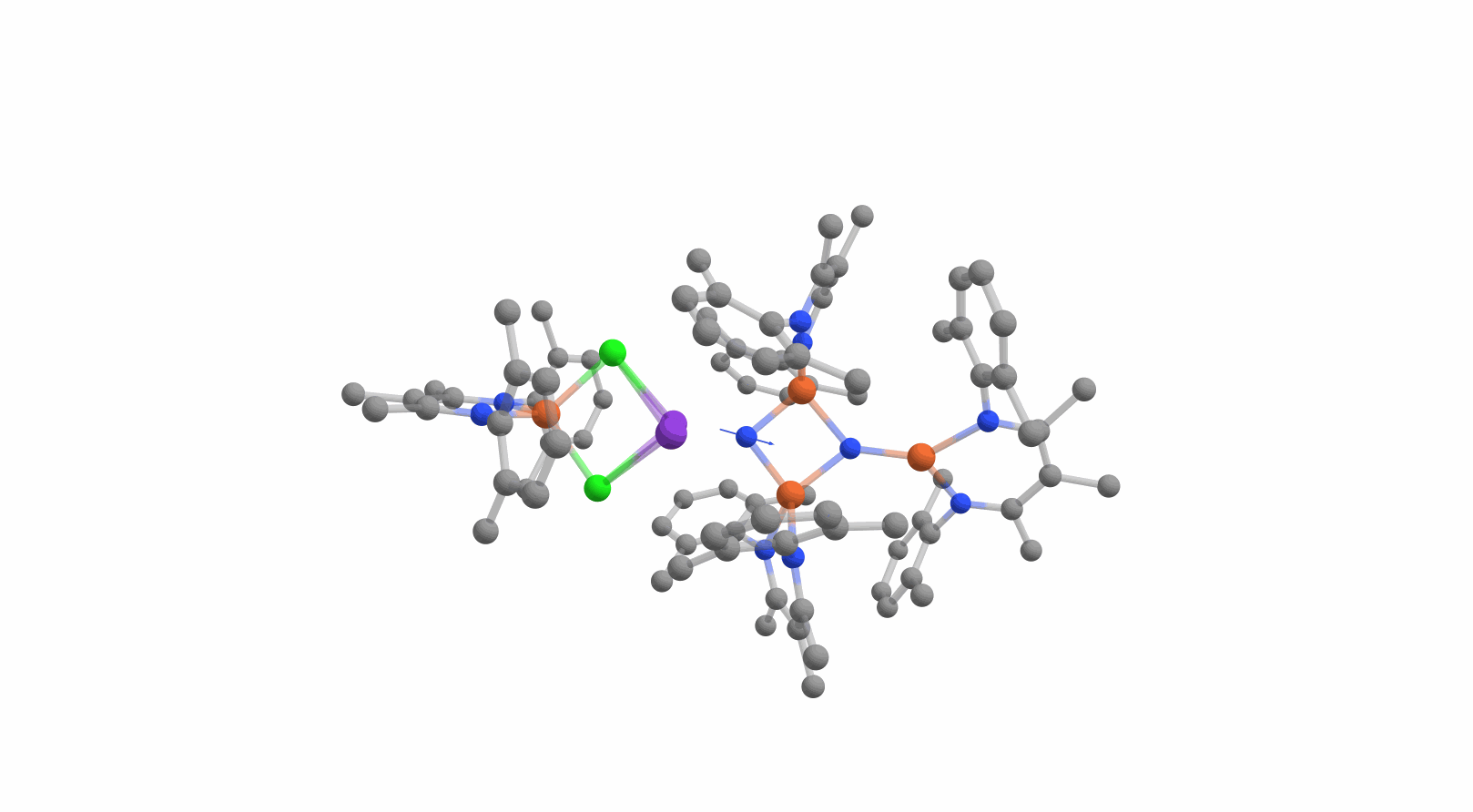

Supplement: Supplementary file 2 — ic3c02594_si_002.zip [file ic3c02594_si_002.zip › animations/KBisN_73_4b.gif]

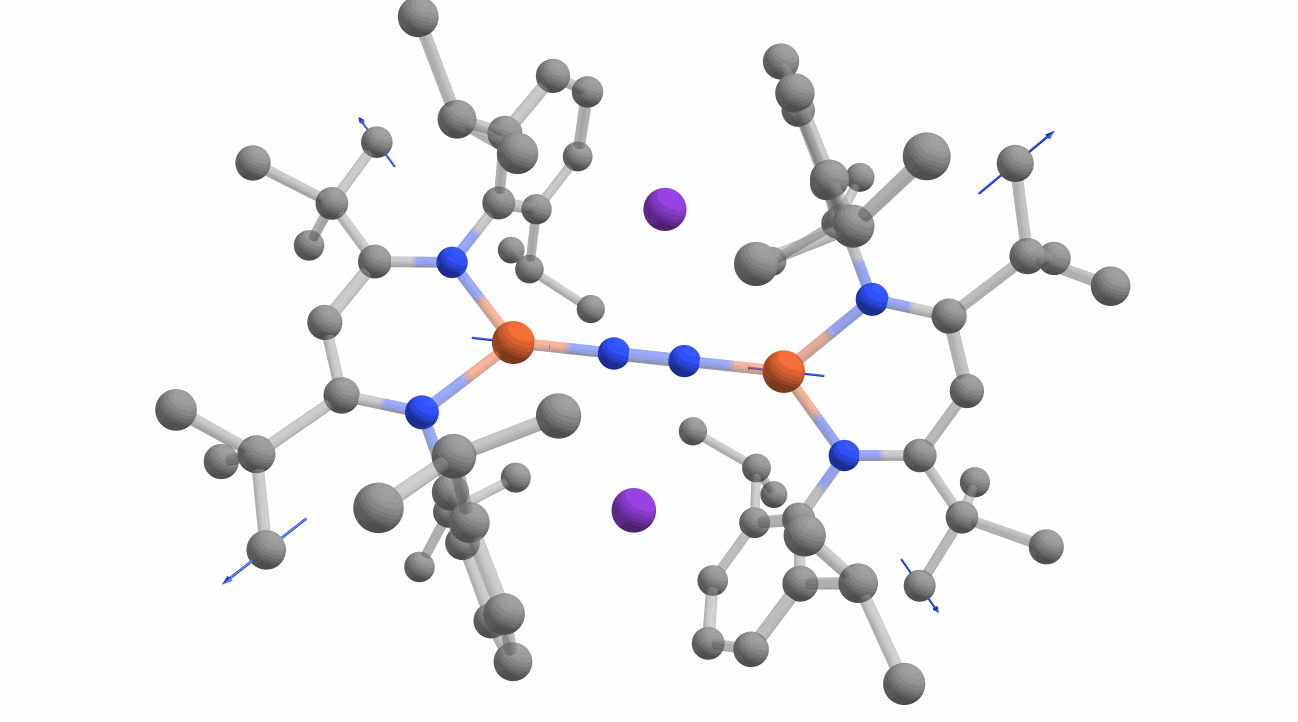

Supplement: Supplementary file 2 — ic3c02594_si_002.zip [file ic3c02594_si_002.zip › animations/K2tBuFeNNFeL_41_4.gif]

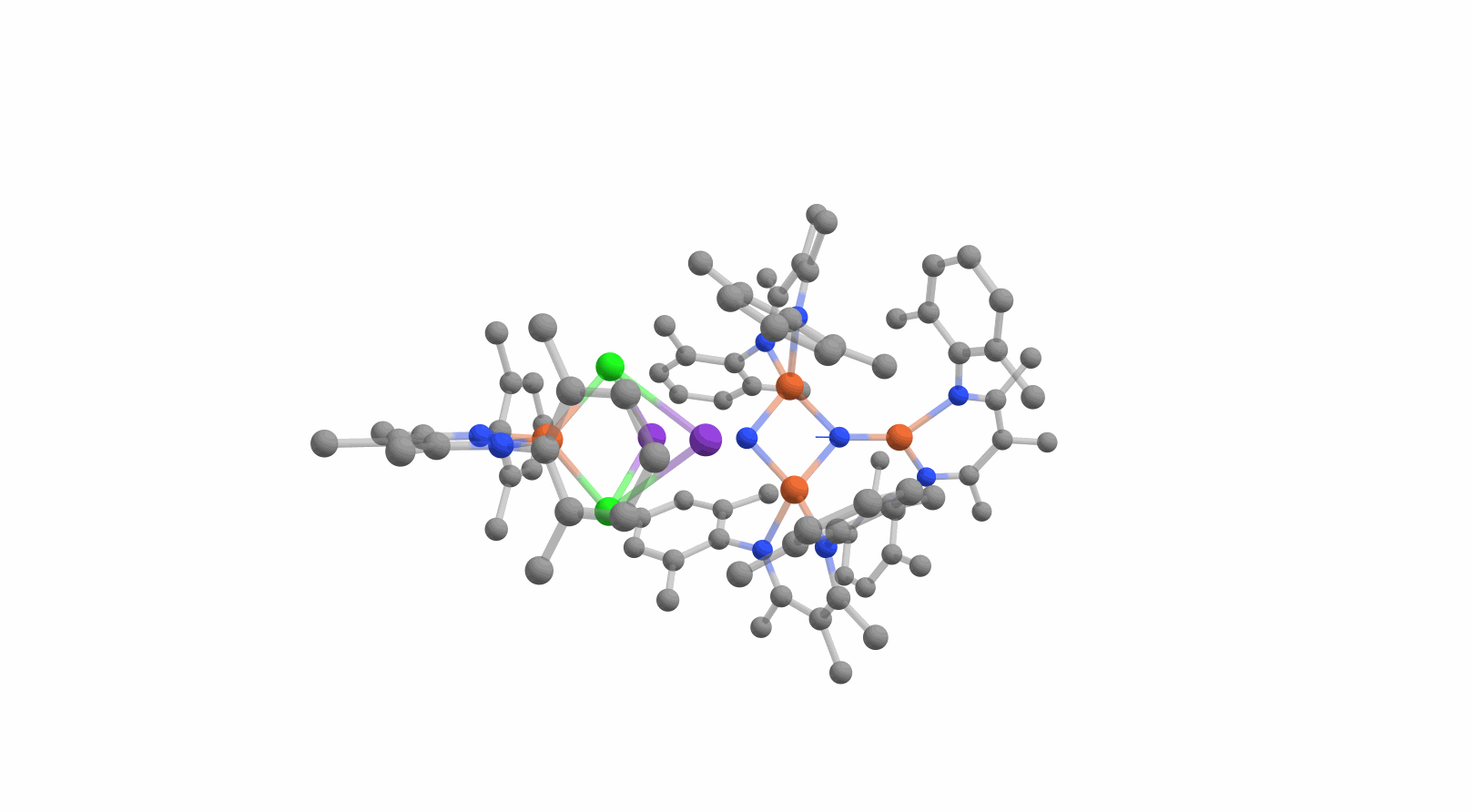

Supplement: Supplementary file 2 — ic3c02594_si_002.zip [file ic3c02594_si_002.zip › animations/KBisN_91_3.gif]

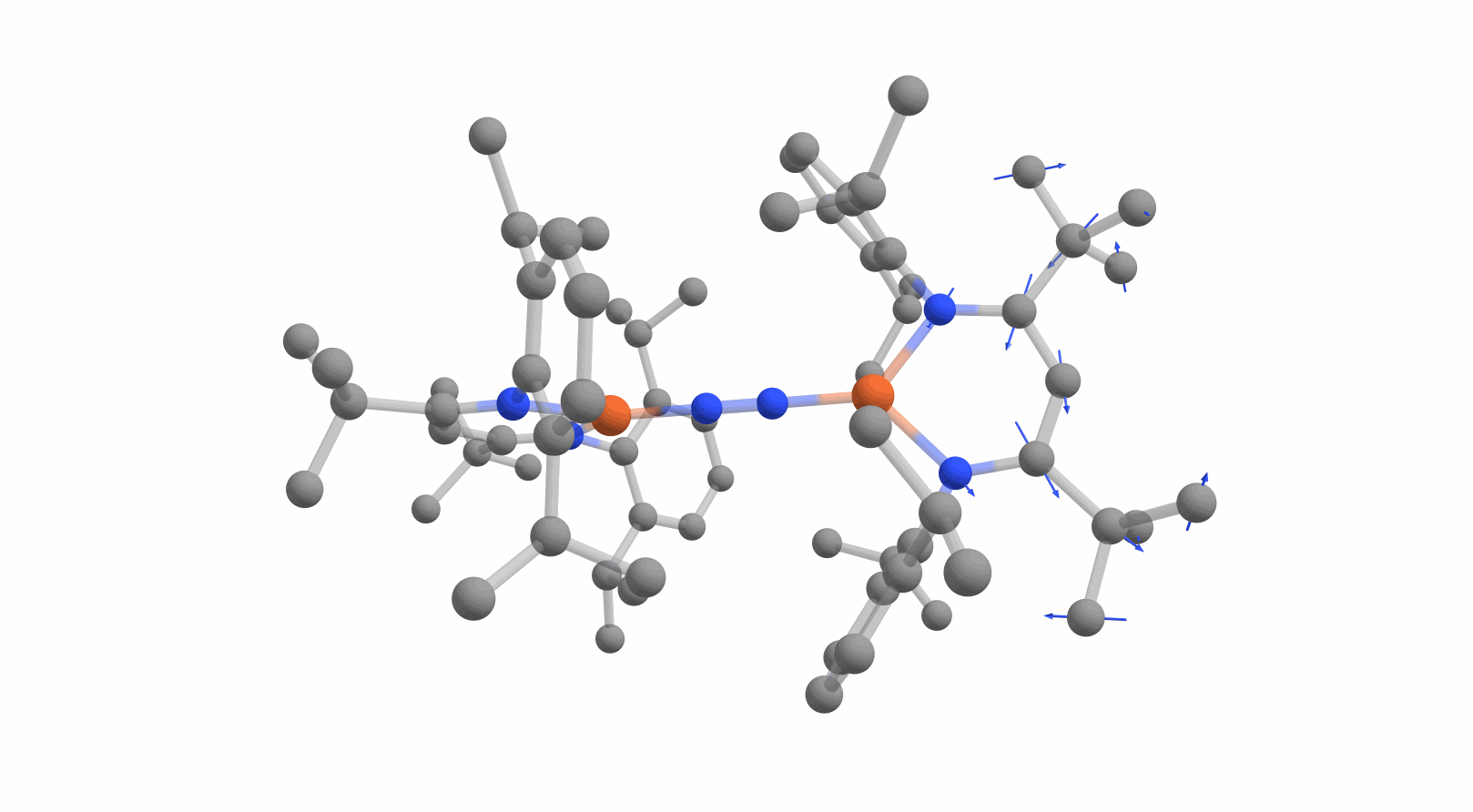

Supplement: Supplementary file 2 — ic3c02594_si_002.zip [file ic3c02594_si_002.zip › animations/tBuFeNNFeL_58_5.gif]

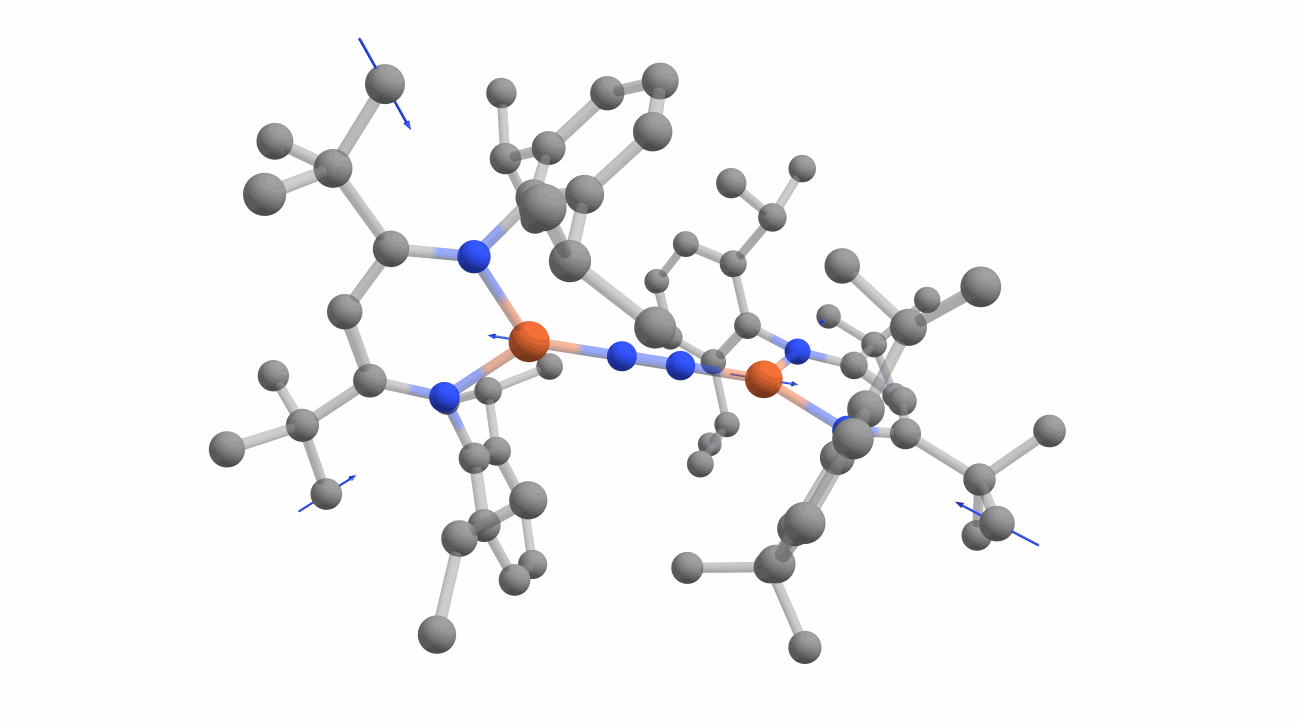

Supplement: Supplementary file 2 — ic3c02594_si_002.zip [file ic3c02594_si_002.zip › animations/tBuFeNNFeL_41_6.gif]

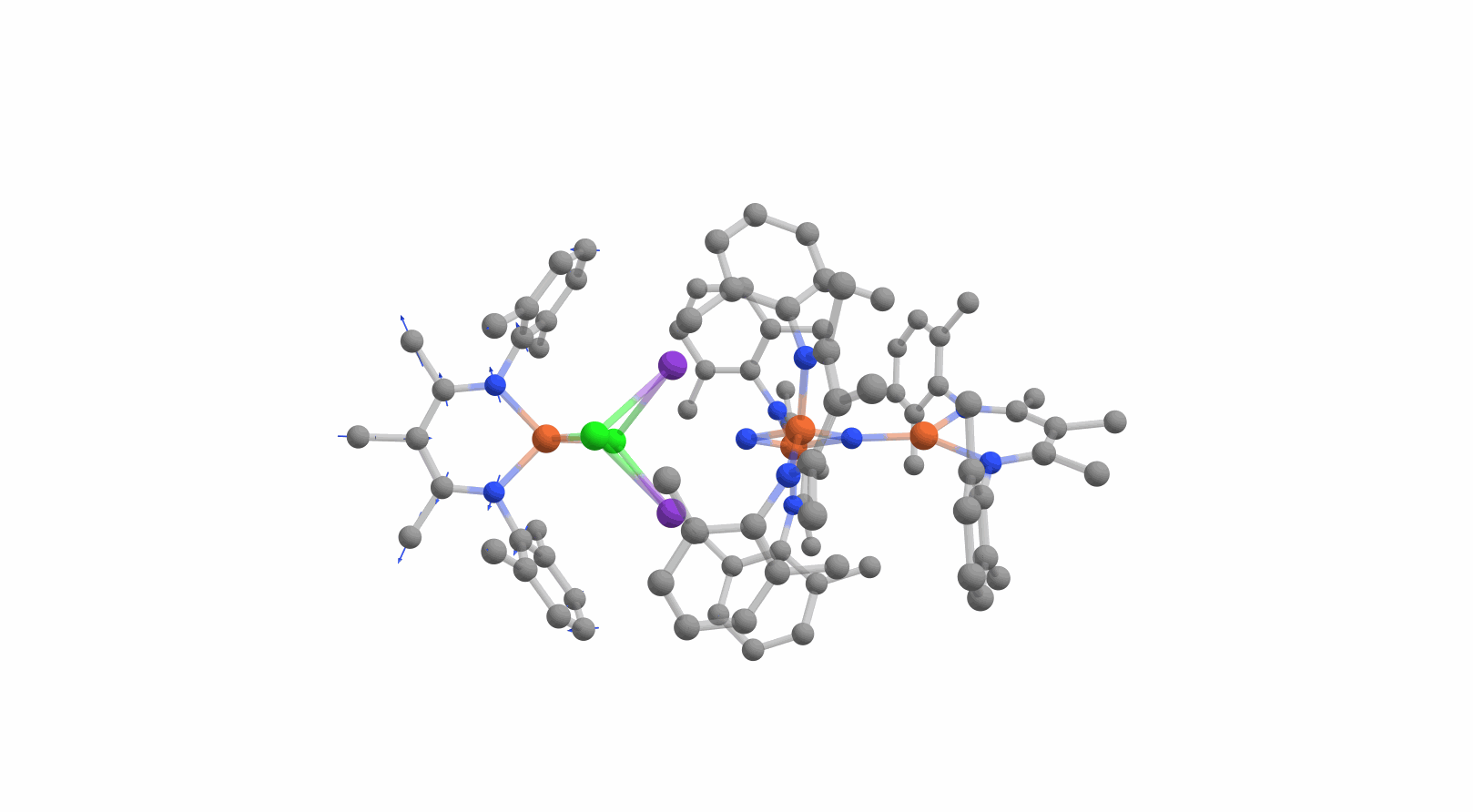

Supplement: Supplementary file 2 — ic3c02594_si_002.zip [file ic3c02594_si_002.zip › animations/KBisN_60_8.gif]

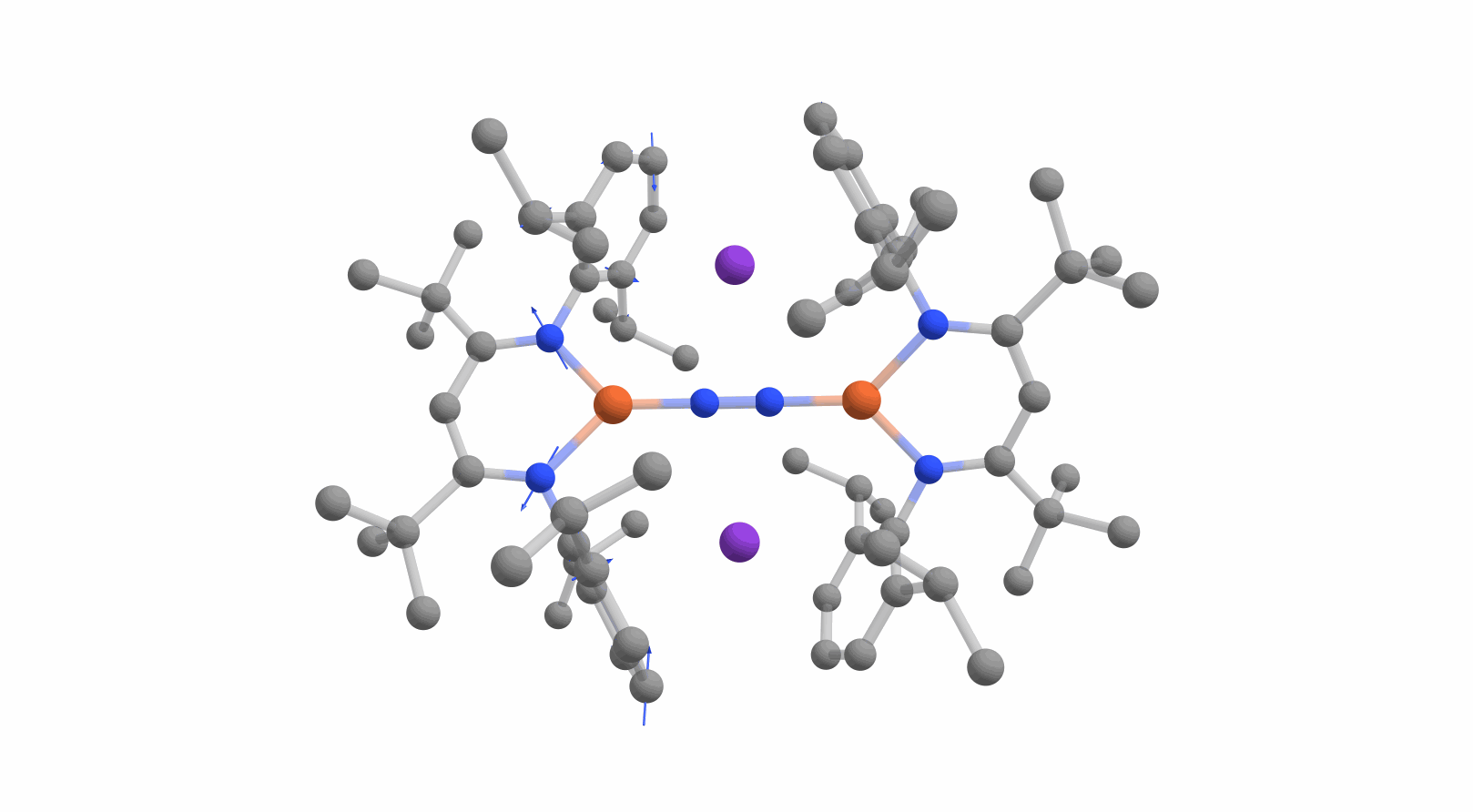

Supplement: Supplementary file 2 — ic3c02594_si_002.zip [file ic3c02594_si_002.zip › animations/K2tBuFeNNFeL_84_0.gif]

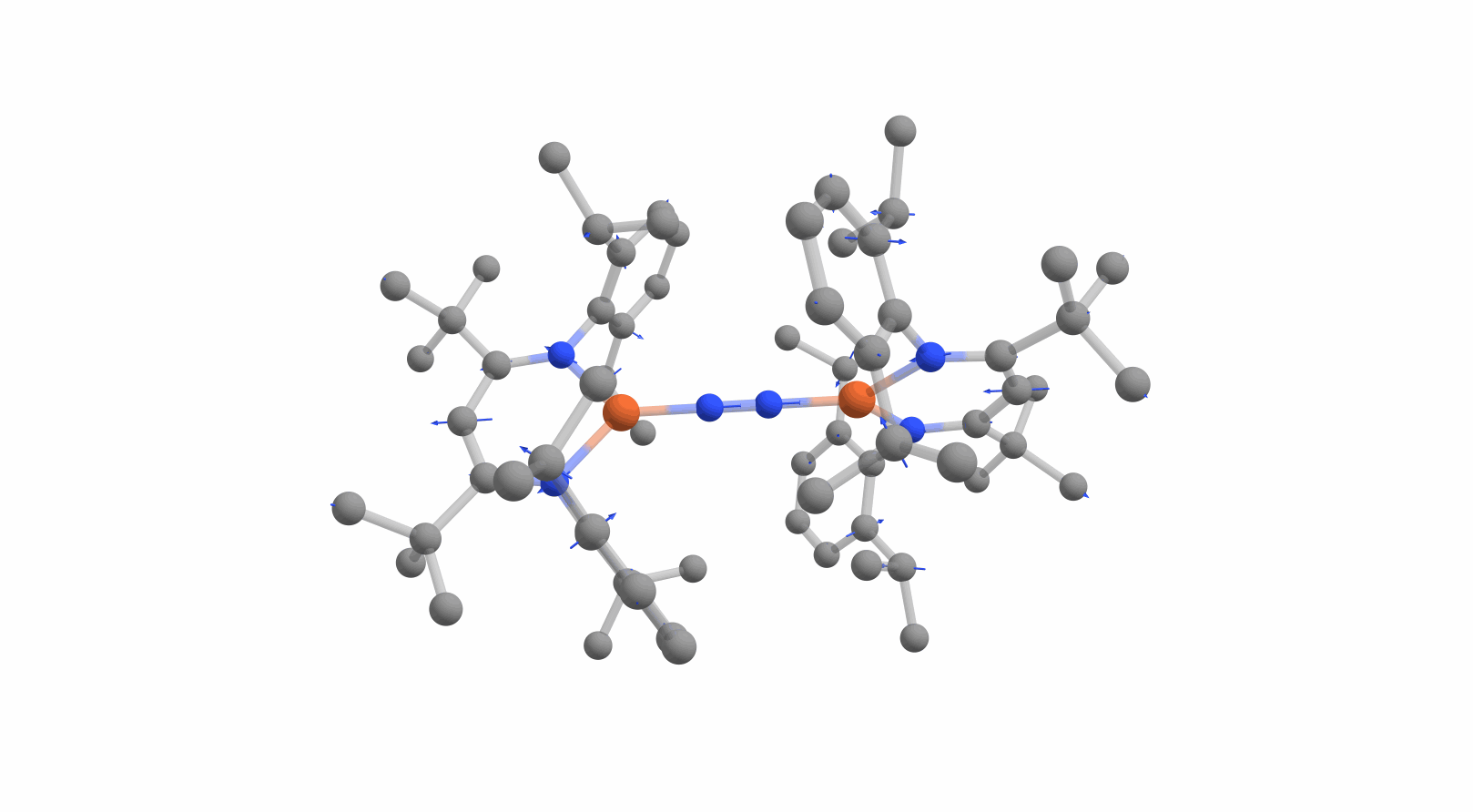

Supplement: Supplementary file 2 — ic3c02594_si_002.zip [file ic3c02594_si_002.zip › animations/tBuFeNNFeL_78_6.gif]

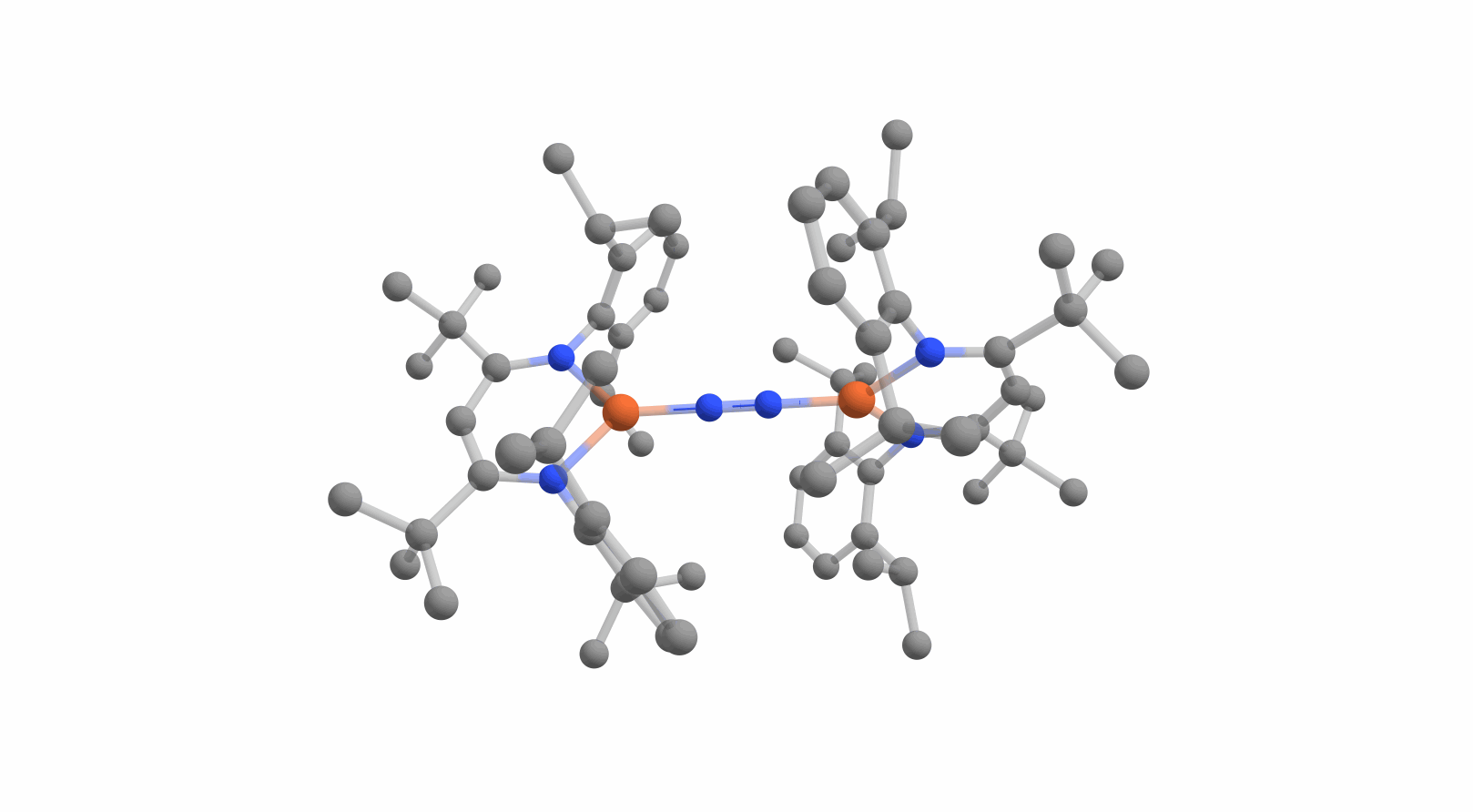

Supplement: Supplementary file 2 — ic3c02594_si_002.zip [file ic3c02594_si_002.zip › animations/tBuFeNNFeL_81_1.gif]
